# Supplementary material for: Effectiveness of Aedes-borne infectious disease control in Latin America and the Caribbean region: A scoping review
Source: PLoS One. 2022 Nov 2;17(11):e0277038. doi: 10.1371/journal.pone.0277038 (PMC9629598; doi:10.1371/journal.pone.0277038)
Supplement: S2 Table — (DOCX) [file pone.0277038.s002.docx]

**S2** Table Search strategy

| **Mosquito-borne disease** | **Location** | **Intervention** |
| --- | --- | --- |
| MeSH Vector Borne Diseases  MeSH Mosquito Vectors  MeSH Arboviruses  MeSH Dengue  MeSH Zika Virus  MeSH Chikungunya virus  MeSH Malaria  MeSH Encephalitis, arbovirus  Ti AB Vector Borne Disease  Ti AB Mosquito-Borne Disease  Ti AB Mosquito  Ti AB Arbovirus  Ti Ab Dengue  Ti Ab Zika  Ti Ab Chikungunya  Ti Ab Malaria  Ti Ab Encephalitis, arbovirus | MeSH Caribbean Region  MeSH Latin America  MeSH South America  Ti Ab [Caribbean]  Ti Ab [Anegada]  Ti Ab [Anguilla]  Ti Ab [Antigua]  Ti Ab [Aruba]  Ti Ab [Bahamas]  Ti Ab [Barbados]  Ti Ab [Barbuda]  Ti Ab [Bonaire]  Ti Ab [Cayman Islands]  Ti Ab [Cuba]  Ti Ab [Curaçao]  Ti Ab [Dominica]  Ti Ab [Dominican Republic]  Ti Ab [Grenada]  Ti Ab [Grenadines]  Ti Ab [Guadeloupe]  Ti Ab [Haiti]  Ti Ab [Hispaniola]  Ti Ab [Jamaica]  Ti Ab [Jost Van Dyke]  Ti Ab [Martinique]  Ti Ab [Montserrat]  Ti Ab [Nevis]  Ti Ab [Puerto Rico]  Ti Ab [Saba]  Ti Ab [Saint Croix]  Ti Ab [Saint Martin]  Ti Ab [Saint Kitts]  Ti Ab [Sint Eustatius]  Ti Ab [Saint Barthélemy]  Ti Ab [Saint John]  Ti Ab [Saint Lucia]  Ti Ab [Saint Martin]  Ti Ab [Saint Thomas]  Ti Ab [Saint Vincent]  Ti Ab [Tortola]  Ti Ab [Trinidad and Tobago]  Ti Ab [Turks and Caicos Islands]  Ti Ab [Virgin Gorda]  Ti Ab [Water Island]  Ti Ab [Latin America]  Ti Ab [Belize]  Ti Ab [Costa Rica]  Ti Ab [El Salvador]  Ti Ab [Guatemala]  Ti Ab [Honduras]  Ti Ab [Mexico]  Ti Ab [Nicaragua]  Ti Ab [Panama]  Ti Ab [Argentina]  Ti Ab [Bolivia]  Ti Ab [ Brazil]  Ti Ab [Chile]  Ti Ab [Colombia]  Ti Ab [Ecuador]  Ti Ab [French Guiana]  Ti Ab [Guyana]  Ti Ab [Paraguay]  Ti Ab [Peru]  Ti Ab [Suriname]  Ti Ab [Uruguay]  Ti Ab [Venezuela] | MeSH mosquito control  MeSH mosquito nets  Ti Ab mosquito control  Ti Ab mosquito net  Ti Ab vector control  Ti Ab insect repellent  Ti Ab residual spraying  Ti Ab integrated vector control  Ti mosquito  Ti control  Ti prevent  Ti impact  Ti evaluation  Ti management |

**Note:** Ti and Ab mean title and abstract, respectively. MeSH means Medical Subject Headings.

**Search log Pubmed and Scopus**

|  | **Database** | **Author responsible** | **Date of search** | **Search string** | **Hits** | **Search complete (Yes/ No)** | **RIS uploaded (Yes/ No)** | **Remarks (if any)** |
| --- | --- | --- | --- | --- | --- | --- | --- | --- |
| 1 | MEDLINE (via PubMed) | VJ | 27-05-21 | #1: ("vector borne diseases"[MeSH Terms] OR "mosquito vectors"[MeSH Terms] OR "arboviruses"[MeSH Terms] OR "Dengue"[MeSH Terms] OR "zika virus"[MeSH Terms] OR "chikungunya virus"[MeSH Terms] OR "Malaria"[MeSH Terms] OR "vector borne disease*"[Title/Abstract] OR "mosquito borne disease*"[Title/Abstract] OR "mosquito*"[Title/Abstract] OR "arbovirus*"[Title/Abstract] OR "Dengue"[Title/Abstract] OR "Zika"[Title/Abstract] OR "Chikungunya"[Title/Abstract] OR "Malaria"[Title/Abstract] OR "encephalitis, arbovirus"[MeSH Terms] OR "encephalitis arbovirus"[Title/Abstract]) AND 2000/01/01:2021/12/31[Date - Publication]  #2: (("caribbean region"[MeSH Terms] OR "latin america"[MeSH Terms] OR "south america"[MeSH Terms] OR "latin americ*"[Title/Abstract] OR "caribbean*"[Title/Abstract] OR "Caribbean"[Title/Abstract] OR "Anegada"[Title/Abstract] OR "Anguilla"[Title/Abstract] OR "Antigua"[Title/Abstract] OR "Aruba"[Title/Abstract] OR "Bahamas"[Title/Abstract] OR "Barbados"[Title/Abstract] OR "Barbuda"[Title/Abstract] OR "Bonaire"[Title/Abstract] OR "cayman islands"[Title/Abstract] OR "Cuba"[Title/Abstract] OR "Curacao"[Title/Abstract] OR "Dominica"[Title/Abstract] OR "dominican republic"[Title/Abstract] OR "Grenada"[Title/Abstract] OR "Grenadines"[Title/Abstract] OR "Guadeloupe"[Title/Abstract] OR "Haiti"[Title/Abstract] OR "Hispaniola"[Title/Abstract] OR "Jamaica"[Title/Abstract] OR ("Jost"[All Fields] AND "van dyke"[Title/Abstract]) OR "Martinique"[Title/Abstract] OR "Montserrat"[Title/Abstract] OR "Nevis"[Title/Abstract] OR "puerto rico"[Title/Abstract] OR "Saba"[Title/Abstract] OR "saint croix"[Title/Abstract] OR "saint martin"[Title/Abstract] OR "saint kitts"[Title/Abstract] OR "sint eustatius"[Title/Abstract] OR "saint barthelemy"[Title/Abstract] OR "saint john"[Title/Abstract] OR "saint lucia"[Title/Abstract] OR "saint thomas"[Title/Abstract] OR "saint vincent"[Title/Abstract] OR "Tortola"[Title/Abstract] OR "Trinidad"[Title/Abstract] OR "Tobago"[Title/Abstract] OR "Turks"[Title/Abstract] OR "caicos islands"[Title/Abstract] OR "virgin gorda"[Title/Abstract] OR "water island"[Title/Abstract] OR "latin america"[Title/Abstract] OR "Belize"[Title/Abstract] OR "costa rica"[Title/Abstract] OR "el salvador"[Title/Abstract] OR "Guatemala"[Title/Abstract] OR "Honduras"[Title/Abstract] OR "Mexico"[Title/Abstract] OR "Nicaragua"[Title/Abstract] OR "Panama"[Title/Abstract] OR "Argentina"[Title/Abstract] OR "Bolivia"[Title/Abstract] OR "Brazil"[Title/Abstract] OR "Chile"[Title/Abstract] OR "Colombia"[Title/Abstract] OR "Ecuador"[Title/Abstract] OR "french guiana"[Title/Abstract] OR "Guyana"[Title/Abstract] OR "Paraguay"[Title/Abstract] OR "Peru"[Title/Abstract] OR "Suriname"[Title/Abstract] OR "Uruguay"[Title/Abstract] OR "Venezuela"[Title/Abstract]) AND 2000/01/01:2021/12/31[Date - Publication])  #3:(("mosquito control"[MeSH Terms] OR "mosquito nets"[MeSH Terms] OR "mosquito control"[Title/Abstract] OR "mosquito net*"[Title/Abstract] OR "vector control"[Title/Abstract] OR "insect repellent*"[Title/Abstract] OR "mosquito*"[Title] OR "Control"[Title] OR "prevent*"[Title] OR "impact"[Title] OR "evaluat*"[Title] OR "manag*"[Title] OR "residual spraying"[Title/Abstract] OR "integrated vector control"[Title/Abstract]) AND 2000/01/01:2022/12/31[Date - Publication])) AND (humans[Filter])  #1 AND #2 AND #3 | 2038 | Yes | Yes | - |
| 2 | Scopus | VJ | 20-05-21 | ( ( TITLE-ABS-KEY ( vector  AND  borne  AND  disease* )  OR  TITLE-ABS-KEY ( mosquito  AND  vectors )  OR  TITLE-ABS-KEY ( arbovirus* )  OR  TITLE-ABS-KEY ( mosquito  AND  borne  AND  disease* ) ) )  AND  ( ( TITLE-ABS-KEY ( caribbean  AND  region )  OR  TITLE-ABS-KEY ( latin  AND  americ* )  OR  TITLE-ABS-KEY ( south  AND  america )  OR  TITLE-ABS-KEY ( caribbean* )  OR  TITLE-ABS-KEY ( anegada )  OR  TITLE-ABS-KEY ( anguilla )  OR  TITLE-ABS-KEY ( antigua )  OR  TITLE-ABS-KEY ( aruba )  OR  TITLE-ABS-KEY ( bahamas )  OR  TITLE-ABS-KEY ( barbados )  OR  TITLE-ABS-KEY ( barbuda )  OR  TITLE-ABS-KEY ( bonaire )  OR  TITLE-ABS-KEY ( cayman  AND  islands )  OR  TITLE-ABS-KEY ( cuba )  OR  TITLE-ABS-KEY ( curacao )  OR  TITLE-ABS-KEY ( dominica )  OR  TITLE-ABS-KEY ( dominican  AND  republic )  OR  TITLE-ABS-KEY ( grenada )  OR  TITLE-ABS-KEY ( grenadines )  OR  TITLE-ABS-KEY ( guadeloupe )  OR  TITLE-ABS-KEY ( haiti )  OR  TITLE-ABS-KEY ( hispaniola )  OR  TITLE-ABS-KEY ( jamaica )  OR  TITLE-ABS-KEY ( jost  AND  van  AND  dyke )  OR  TITLE-ABS-KEY ( martinique )  OR  TITLE-ABS-KEY ( montserrat )  OR  TITLE-ABS-KEY ( nevis )  OR  TITLE-ABS-KEY ( puerto  AND  rico )  OR  TITLE-ABS-KEY ( saba )  OR  TITLE-ABS-KEY ( saint  AND  croix )  OR  TITLE-ABS-KEY ( saint  AND  martin )  OR  TITLE-ABS-KEY ( saint  AND  kitts )  OR  TITLE-ABS-KEY ( sint  AND  eustatius )  OR  TITLE-ABS-KEY ( saint  AND  barthelemy )  OR  TITLE-ABS-KEY ( saint  AND  john )  OR  TITLE-ABS-KEY ( saint  AND  lucia )  OR  TITLE-ABS-KEY ( saint  AND  thomas )  OR  TITLE-ABS-KEY ( saint  AND  vincent )  OR  TITLE-ABS-KEY ( tortola )  OR  TITLE-ABS-KEY ( trinidad )  OR  TITLE-ABS-KEY ( tobago )  OR  TITLE-ABS-KEY ( turks )  OR  TITLE-ABS-KEY ( caicos  AND  islands )  OR  TITLE-ABS-KEY ( virgin  AND  gorda )  OR  TITLE-ABS-KEY ( water  AND  island )  OR  TITLE-ABS-KEY ( belize )  OR  TITLE-ABS-KEY ( costa  AND  rica )  OR  TITLE-ABS-KEY ( el  AND  salvador )  OR  TITLE-ABS-KEY ( guatemala )  OR  TITLE-ABS-KEY ( honduras )  OR  TITLE-ABS-KEY ( mexico )  OR  TITLE-ABS-KEY ( nicaragua )  OR  TITLE-ABS-KEY ( panama )  OR  TITLE-ABS-KEY ( argentina )  OR  TITLE-ABS-KEY ( bolivia )  OR  TITLE-ABS-KEY ( brazil )  OR  TITLE-ABS-KEY ( chile )  OR  TITLE-ABS-KEY ( colombia )  OR  TITLE-ABS-KEY ( ecuador )  OR  TITLE-ABS-KEY ( french  AND  guiana )  OR  TITLE-ABS-KEY ( guyana )  OR  TITLE-ABS-KEY ( paraguay )  OR  TITLE-ABS-KEY ( peru )  OR  TITLE-ABS-KEY ( suriname )  OR  TITLE-ABS-KEY ( uruguay )  OR  TITLE-ABS-KEY ( venezuela ) ) )  AND  ( ( TITLE-ABS-KEY ( mosquito  AND  control )  OR  TITLE-ABS-KEY ( mosquito  AND  nets )  OR  TITLE-ABS-KEY ( vector  AND  control )  OR  TITLE-ABS-KEY ( insect  AND  repellent* )  OR  TITLE-ABS-KEY ( prevent* )  OR  TITLE-ABS-KEY ( evaluat* )  OR  TITLE-ABS-KEY ( manag* )  OR  TITLE-ABS-KEY ( residual  AND  spraying )  OR  TITLE-ABS-KEY ( integrated  AND  vector  AND  control ) ) )  AND  ( humans )  AND  ( LIMIT-TO ( PUBYEAR ,  2021 )  OR  LIMIT-TO ( PUBYEAR ,  2020 )  OR  LIMIT-TO ( PUBYEAR ,  2019 )  OR  LIMIT-TO ( PUBYEAR ,  2018 )  OR  LIMIT-TO ( PUBYEAR ,  2017 )  OR  LIMIT-TO ( PUBYEAR ,  2016 )  OR  LIMIT-TO ( PUBYEAR ,  2015 )  OR  LIMIT-TO ( PUBYEAR ,  2014 )  OR  LIMIT-TO ( PUBYEAR ,  2013 )  OR  LIMIT-TO ( PUBYEAR ,  2012 )  OR  LIMIT-TO ( PUBYEAR ,  2011 )  OR  LIMIT-TO ( PUBYEAR ,  2010 )  OR  LIMIT-TO ( PUBYEAR ,  2009 )  OR  LIMIT-TO ( PUBYEAR ,  2008 )  OR  LIMIT-TO ( PUBYEAR ,  2007 )  OR  LIMIT-TO ( PUBYEAR ,  2006 )  OR  LIMIT-TO ( PUBYEAR ,  2005 )  OR  LIMIT-TO ( PUBYEAR ,  2004 )  OR  LIMIT-TO ( PUBYEAR ,  2003 )  OR  LIMIT-TO ( PUBYEAR ,  2002 )  OR  LIMIT-TO ( PUBYEAR ,  2001 )  OR  LIMIT-TO ( PUBYEAR ,  2000 ) ) | 2571 | Yes | Yes | - |
