# Supplementary material for: Effectiveness of Aedes-borne infectious disease control in Latin America and the Caribbean region: A scoping review
Source: PLoS One. 2022 Nov 2;17(11):e0277038. doi: 10.1371/journal.pone.0277038 (PMC9629598; doi:10.1371/journal.pone.0277038)
Supplement: S3 Table — (DOCX) [file pone.0277038.s003.docx]

S3 Table Characteristics of studies identified in the Latin America region about *Ae. aegypti* and *Ae. albopictus* prevention and control interventions

|  | **Country** | **Author, year** | **Objective (s)** | **Study design** | **Type of Intervention (s)** | **Outcome (s)** | **Main results** |
| --- | --- | --- | --- | --- | --- | --- | --- |
| 1 | Argentina | Masuh, 2003 | To evaluate the efficacy of the fumigant canister CIPEIN pF-7 for mosquito control under field conditions. | Pre-post (before-after) study | Application of insecticides. Fumigant canister CIPEIN pF-7 containing 120 g of fumigant mixture and 6 g beta-cypermethrin. | Breteau index, house index, and mosquitos’ density | There was 100% initial mortality in each case after treatment with the fumigant canister. House and Breteau indices before the treatment were 51% and 106, respectively, falling to 23% and 44 after treatment. |
| 2 | Argentina | Gürtler, 2009 | To determine the effect of a citywide intervention on *Ae. aegypti* larval indices and the reported incidence of dengue in Clorinda, north-eastern Argentina, over 2003–2007. | Pre-post (before-after) study | Removal of mosquito breeding sites, treat water containers (non-drinking water) with 1% temephos or Bti, and application of ultra-low volume insecticide. | Breteau index, house index, pupae per 100 houses index, and incidence of dengue | House indices declined from 13.7% at baseline to 3.7% at the second focal cycle, and the Breteau index fell from 19.0 to 4.8. House indices were significantly reduced by 13–54% relative to pre-intervention indices in cycles 2–3, 5–7, 9, and 14. The reported incidence of dengue declined from 10.4 per 10,000 in 2000 (by DEN-1) to 0 from 2001 to 2006 and then rose to 4.5 cases per 10,000 in 2007 (by DEN-3). |
| 3 | Argentina | Harburguer, 2011 | To evaluate the efficacy of an experimental nonprofessional fumigant formulation against *Ae. aegypti* in the field, developed in alaboratory, and the residents’ acceptance of this tool together with its role in community participation for indoor control activities. | ‘Non Randomised Controlled trial (NRCT) | Application of insecticides. Fumigant tablet containing 10% permethrin (3-phenoxyphenyl) methyl 3-(2,2 dichloroethenyl) -2,2-(dimethyl cyclopropane carboxylate), *cis:trans* relationship (45:55), and 2% pyriproxyfen (2-[1-methyl-2-(4-phenoxyphenoxy) ethoxy] pyridine). Ultra-low volume treatment, 10% permethrin plus 2% pyriproxyfen. | Breteau index, adult mosquito index, and community participation | The findings showed >90% adult emergence inhibition and 100% adult mortality with these treatments. More than 80% of the residents applied the fumigant tablet and preferred participating in a vector control program using a nonprofessional mosquito control tool instead of attending meetings and workshops promoting cultural changes. |
| 4 | Brazil | Camargo Donalisio, 2002 | To evaluate the efficacy of temephos for the control of the larval population of *Ae. aegypti* as a part of a control program’s  routine. | NRCT | Application of insecticide: temephos 1 ppm and source reduction. | Breteau index, and container index | The intervention area presented similar levels of *Ae. aegypti* larval infestation as the untreated area. |
| 5 | Brazil | Madeira, 2002 | To evaluate a teaching method concerning the vector and dengue. | NRCT | Health education campaign at school. | Knowledge | The students who received the intervention were more successful in identifying the cycle’s stages, biological characteristics of the adult insect, and the mosquito’s importance in health issues. |
| 6 | Brazil | Chiaravalloti Neto, 2003* | To identify changes in knowledge and practices learned to prevent dengue fever in two areas of Catanduva, São Paulo State, from 1999 to 2001. | NRCT | Health education campaign. | Knowledge attitude, practices (KAP) and Breteau index | Breeding sites were significantly reduced. The proportion of houses without breeding sites was significantly increased. There was an increase in the percentage of individuals who recognised the larval form of the vector in the study area. |
| 7 | Brazil | Perich, 2003 | To test the lethal ovitrap against the dengue vector populations in urban areas of Brazil. | Randomised Controlled trial (RCT) | Usage of lethal ovitrap | The total number of containers per house containing mosquito larvae and/or pupae, the mean number of mosquito pupae found per house, and adult mosquitoes | Post-intervention densities of *Ae. aegypti* were significantly reduced for most comparators (P < 0.01), as shown by fewer positive containers (4–5 vs 10–18) and pupae/house (0.3–0.7 vs 8–10) at intervention vs control group. Numbers of adult *Ae. aegypti* females indoors were consistently reduced in the intervention group at Areia Branca but not at Niloplis. |
| 8 | Brazil | Pamplona, 2004* | To evaluate the results of an intervention with the fish *Betta splendens* to control immature forms of *Ae. aegypti*, in cement tanks in the municipality of Canindé, Ceará. | Pre-post (before-after) study | Usage of the fish *Betta splendens* | Immature forms *Ae. aegypti* | In January 2001, 70.4% of the water tanks presented mosquitoes. Following the intervention, in January 2002, only 7.4% were positive, and by December 2002, the rate had dropped to 0.2%. |
| 9 | Brazil | Chiaravalloti Neto, 2006* | To assess if the introduction of dengue control in the Family Health Program would produce gains in knowledge about dengue, its vector, and control measures concerning the traditional program, and to assess whether they would occur changes in practices for vector control in the populations served. | NRCT | Combining the Family Health Program and the Dengue Control Program. House inspection, elimination of potential breeding sites of *Ae. aegypti,* and reorganise the work of the vector control team by carrying out visits to unregistered non-residential properties and vacant lots. | KAP and building index | There were significant changes concerning the increased knowledge about the disease and reduction in mosquito breeding sites. The building index measured in the intervention region were 6.9% (before) and (after) 4.4%, with a significant difference (p = 0.040). The findings show that integration between the programs is possible and could help optimise resources. |
| 10 | Brazil | Favier, 2006 | To determine the influence of climate and environmental vector control with or without insecticide on *Ae. aegypti* larval indices and pupae density. | NRCT | Application of insecticides. Four interventions with the combination of environmental management and insecticide/biological control treatment (Methoprene, Bti, or Temephos). | Percentage of premises with potential breeding sites, the mean number of potential breeding sites per premise, house index, container index, and Breteau index | Environmental vector control strategies dramatically decreased infestation in the five areas. No significant differences could be detected between control strategies with insecticide and without. |
| 11 | Brazil | Regis, 2008 | To evaluate a new approach to dengue vector surveillance based on permanent egg collection using a modified ovitrap and Bti. | Pre-post (before-after) study | Mass collection of *Ae.* Eggs with ovitraps with Bti. | *Ae.* eggs density | Massive egg collection carried out at one of the sites prevented such a population outbreak. Egg counts made it possible to identify spots where the vector population is consistently concentrated over time, pinpointing areas that should be considered a high priority for control activities. |
| 12 | Brazil | Santos, 2008* | To evaluate novel approaches to monitor and control *Ae. aegypti* in Recife Metropolitan municipalities, from 2001 to 2007. | NRCT | Mass collection of *Ae.* Eggs with ovitraps with Bti. | *Ae.* eggs density | The massive collection and destruction of *Ae. aegypti* integrated with the elimination or treatment of breeding sites can negatively affect the population density of this species. The studies demonstrated that the use of control ovitraps treated with Bti can be operationalised on a large scale, in the context of small or large municipalities, with the advantage of removing large amounts of eggs. |
| 13 | Brazil | Pessanha, 2009* | To describe and evaluate dengue patterns after the implementation of the National Dengue Control Plan. | Record-surveillance | National control plan: health education campaigns, epidemiological surveillance, vector reduction. | Incidence of dengue and larval infestation | The goal concerning dengue incidence reduction (50% reduction in dengue cases) was not achieved in 143 of 292 (49%) municipalities analysed. The cities participating in the larval research in October 2005 and October 2006, 54.9% (45/82) in 2005 and 64.6% ( 53/82) in 2006 did not reach the goal of reducing the larval infestation to less than 1%. |
| 14 | Brazil | Maciel-de-Freitas, 2011 | To evaluate the impact of eliminating the most productive container types on *Ae. aegypti* population density in Brazil’s dengue-endemic urban center. | Pre-post (before-after) study | Target the most productive container types: containers were covered using nylon net to prevent mosquito oviposition. | Breteau index, container index, house index, pupae/person index, and pupae per hectare | The most productive containers were: water tanks and metal drums. A short-term decrease in weekly adult female *Ae. aegypti* density after covering 733 water tanks with nylon net was observed. A long-term reduction in female adult population density was achieved only when both water tanks and metal drums were covered. Pupae per person, per hectare, and per house also decreased. |
| 15 | Brazil | Costa, 2011* | To evaluate the “vector control *(Ae. aegypti)*” component of the National Plan control of Dengue, regarding the infrastructure of equipment and human resources and the quality of vector control actions in the city Caruaru, from 2008 to 2009. | Record-surveillance | National Plan control of Dengue: (i) house visits, inspection of strategic points and vacant lots, (ii) chemical treatment of identified foci; usually the larvicide temephos at a rate of 1 ppm was used, (iii) spraying of insecticide at ultra-low volume. | Breteau index, property index, pending rate, and the number of resources including human resources for vector control | In 2008 and 2009, the pending rate and the level of infestation (mosquito) were above the percentage allowed by the Ministry of Health. The coverage of control in these years was lower (67.92 in 2008 and 67.35 in 2009) than recommended (90%) by the National Plan. Similarly, the number of vehicles, equipment, and personnel was insufficient in those years. |
| 16 | Brazil | Luz, 2011 | To calculate the health burden in terms of disability-adjusted life years lost to dengue and the cost-effectiveness of various interventions. | Economic modeling assessment | Application of adulticides and larvicides in the field. | Disability-adjusted life years and cost of interventions | In our model, one or more applications of high-efficacy larval control reduced dengue burden for up to 2 years, whereas three or more applications of high-efficacy adult vector control reduced dengue burden for up to 4 years. For the entire 5-year period, 6 applications of high-efficacy adult vector control reduced the dengue burden to the greatest extent, resulting in 248 disability adjusted life years (DALYs) lost per million individuals. |
| 17 | Brazil | da Silva, 2013* | To analyse the process of cooperation between health personnel and the school in potential dengue vector breeding sites in households. | Mixed method study | Health education campaign at school. | *Ae. aegypti* breeding sites | During the first visit, 83 (89.3%) HHs had some kind of container suitable for *Ae.* breeding. After the health education campaign, the number of HHs with potential breeding sites decreased to (second visits) 65 (70%) and (third visits) 63 (68%). |
| 18 | Brazil | Regis, 2013 | To evaluate and improve the applicability of an environmental friendly vector monitoring and control system to reduce dengue virus transmission. | Pre-post (before-after) study | National Program for Dengue Control: (i) application of temephos, (ii) campaign for source elimination (iii) application of (e.g., organophosporous or piretroids) adulticides through ultra-low volume, (iv) communication campaign, (v) usage of ovitraps loaded with Bti and (vi) indoor collections of adult mosquitoes using aspirators, targeting places considered as highly important for virus transmission. | The density of *Ae*. eggs and adult mosquito population | A 90% decrease in egg density was recorded in Santa Cruz do Capibaribe after two years of sustained control pressure imposed by suppression of >7,500,000 eggs and >3,200 adults, plus larval control by adding fishes to cisterns. In Ipojuca, 1.1 million mosquito eggs were suppressed, and a 77% reduction in egg density was achieved. Data from Ipojuca showed a sharp decrease in the mosquito population. |
| 19 | Brazil | Degener, 2014 | To assess the effectiveness of BG-Sentinel traps for mass trapping at the household level to control the dengue vector, *Ae. aegypti*, in Manaus (Brazil) by performing a cluster randomised controlled trial. | CRT (Cluster randomised trial) | BG-Sentinel traps. | The number of *Ae. aegypti* female mosquitoes and incidence of dengue | The results indicated that mass trapping with BGS traps significantly reduced the abundance of adult female *Ae. aegypti* during the first five rainy months. No effect of mass trapping was observed in the dry season. Fewer *Ae. aegypti* females were measured in the intervention arm during the next rainy period, but no significant difference between arms was observed. The serological survey revealed that recent dengue infections were less common in the intervention area than in control areas, although this effect was not statistically significant. |
| 20 | Brazil | Maciel-de-Freitas, 2014 | To determine the impact of the standard vector control measures. | Record-surveillance | Removal of potential breeding sites, water storage containers were treated with the larvicide diflubenzuron, and deltamethrin was sprayed for adult *Ae. aegypti* mosquitoes. | Breteau index and house index | 94 325 containers were removed from these houses or treated. A slight decrease in vector density was detected; the house index was reduced from 1.7 before interventions to 1.37 immediately after the second survey. |
| 21 | Brazil | Abad-Franch, 2015 | To investigate mosquito-driven dissemination of PPF dust-particles from 100 ‘dissemination stations’ deployed in a 7-ha sub-area to surveillance dwellings and sentinel breeding sites distributed over an urban neighbourhood of about 50 ha. | Pre-post (before-after) study | Dissemination of PPF dust-particles. | Juvenile mosquito mortality, adult mosquito emergence in each sentinel breeding site -month, and breeding-site coverage | There was evidence of PPF contamination in 75.5%, 80%, 100%, and 94.4% of surveillance dwellings in months 11, 12, 13, and 14, respectively. Juvenile mosquito mortality in sentinel breeding sites (about 4% at baseline) increased by over one order of magnitude during PPF dissemination (about 75%). This led to a >10-fold decrease of adult mosquito emergence from sentinel breeding sites, from approximately 1,000–3,000 adults/month before to about 100 adults/month during PPF dissemination. |
| 22 | Brazil | Caprara, 2015 | To control both productive container types and discarded containers through an Eco health approach and analyse its effectiveness in reducing *Ae. aegypti* vector density. | CRT | Health education campaign, focussed on community mobilisation, including clean-up campaigns, covering the elevated containers and in-house rubbish disposal without larvicide. | Breteau index, house index, container index, pupae per person index, community participation, and cost of the intervention | There was an important reduction in small water containers in the intervention clusters (100% elimination in all visited houses). Before and after the intervention differences were identified between the intervention and control areas. The house index, container index, Breteau index, and pupae per person index increased, as expected, from the dry season (before intervention) to the rainy season (after the intervention), but the increase was significantly higher in the control clusters (p-values: house index=0.029 container index=0.020, Breteau index=0.014, pupae per person index=0.023) demonstrating the protective efficacy of the intervention. |
| 23 | Brazil | Degener, 2015 | To evaluate the effectiveness of *Ae.* aegypti mass trapping using the sticky trap MosquiTRAP (MQT) by performing a cluster randomised controlled trial in Manaus, state of Amazonas, Brazil. | CRT | Usage of sticky trap MosquiTRAP (MQT). | The number of *Ae. aegypti* females, the frequency of dengue virus IgM seropositivity, perceived trap effectiveness, problems, improvements, user satisfaction and KAP | Entomological results indicated that MQT mass trapping did not reduce adult *Ae. aegypti* abundance. The serological survey indicated that recent dengue infections were equally frequent in the intervention and the control group. |
| 24 | Brazil | Alecrim, 2016* | To describe dengue control actions developed in Ipatinga in 2009 and 2010, signalling the impact of the measures  implemented (control plan and campaigns) that positively or negatively influenced the reduction of dengue cases in the city. | Record-surveillance | The dengue plan: (i) community mobilisation, (ii) identification of the predominant breeding sites and the infestation situation, (iii) prioritise the groups that have higher rates of infestation and cases notified, in order to intensify actions and contain the situation, and (iv) garbage collection in order to eliminate breeding sites for the *Ae. aegypt*i mosquito. | Incidence of dengue | Within 60 working days more than 70 thousand tons of garbage were collected across the city. All technical and educational means linked to the community mobilisation, were linked with the following finding, it was observed that the municipality obtained positive results in reducing the total number of dengue cases between the years 2009 and 2010. |
| 25 | Brazil | Abad-Franch, 2017 | To test if mosquito-disseminated pyriproxyfen can effectively reduce mosquito populations at the spatial scale relevant for vector control and disease prevention. | Pre-post (before-after) study | Dissemination of pyriproxyfen. | Juvenile and adult mosquito density | Following pyriproxyfen dissemination, *Ae.* juvenile catch decreased by 79%–92% and juvenile mortality increased from 2%–7% to 80%–90%. Mean adult *Ae.* emergence fell from 1,077 per month (range 653–1,635) at baseline to 50.4 per month during pyriproxyfen dissemination (range 2–117). |
| 26 | Brazil | Garziera, 2017 | To assess the related changes in the distribution of infestation and abundance of vector *Ae. aegypti* populations 6 and 18 months after releasing transgenic mosquitoes in two areas in Brazil. | Pre-post (before-after) study | Release of transgenic male *Ae. aegypti* mosquitoes with the OX513A line. | The number of eggs of *Ae. aegypti* (wild) per trap | In both trials, there was an average suppression of ca. 70% of the wild population due to the release of transgenic males compared to the pre-release period. In Juazeiro, in the post-intervention phase, the number of eggs per trap ranged between 0.06 and 14.41 (mean ± SE = 4.44 ± 0.44), and the ovitrap index (OI = number of ovitraps with eggs/total number of ovitraps recovered) ranged from 0.01 to 0.43 (0.13 ± 0.01). In Jacobina, during the post-intervention phase, the number of eggs per trap ranged between 1 and 7.2 (1.72 ± 0.72), and the OI ranged from 1 to 0.83 (0.095 ± 0.032). The mosquito population in Juazeiro remained suppressed for 17 weeks, whereas in Jacobina suppression lasted 32 weeks. |
| 27 | Brazil | Piovezan, 2017 | To compare the application of the insecticide malathion GT 96% by the method of nebulisation with portable equipment vs heavy equipment. | NRCT | Application of insecticide (ultra-low volume) with malathion GT 96% using heavy equipment coupled to a vehicle. | Incidence of dengue and *Ae*. eggs | After the applications with heavy equipment, the number of cases of the disease reported in the month of April for the intervention group was less than half that of the control group. |
| 28 | Brazil | Souza, 2017 | To assess the impact of placing concrete at the bottom of the storm drains to elevate their base to the level of the outflow tube, avoiding water accumulation, and placement of a metal mesh covering the outflow tube to avoid its clogging with debris, on mosquito and water retainment. | Pre-post (before-after) study | The intervention consisted of placing concrete in the bottom of the storm drains, aiming to raise the bottom level equal to that of the outflow draining tube, and thus prevent standing water. In addition, a metal mesh was installed over the outflow drain tube to trap debris and prevent it from entering the drain and restricting water flow. | Accumulated water volume, adult and immature mosquitoes in storm drains | Before the intervention, water accumulated in 48 (92.3%) of the storm drains, and immature *Ae. aegypti* were found in 11 (21.2%) and adults in 10 (19.2%). After the intervention, water accumulated in 5 (9.6%) of the storm drains (P < 0.001), none (0.0%) had immatures (P < 0.001), and 3 (5.8%) contained adults (P = 0.039). |
| 29 | Brazil | Santos, 2018 | To assess if a conjugate of 2 larvicides for *C.quinquefasciatus* and *Ae. aegypti* populations in two contiguous urban subareas in the neighbourhood of Água Fria in Recife City would be more effective than a single one. | NRCT | Application of lysinibacillus sphaericus (Lsp) and Bti. | *Ae.* eggs and adult population | A reduction in the *Ae. Aegypti* adult population was not proven in the area treated with the conjugate mixture, but a significant decrease in egg density was detected in year-2, compared to year-1. |
| 30 | Brazil | Abel Mangueira, 2019 | To evaluate and compare KAP related to the prevention of arboviruses before and after a two-month educational intervention using a learning platform on mobile devices (m-learning and m-health). | NRCT | Health education campaign. Learning platform on mobile devices. | KAP | The students changed their attitudes and behaviour (P = 0.032) concerning their engagements in actions for the prevention of arboviral diseases and several other activities related to house inspections and precautions with water tanks (P < 0.01). |
| 31 | Brazil | Marini, 2019 | To estimate the proportion of cases avoided by ultra-low volume insecticide spraying in Porto Alegre, a Brazilian metropolis characterised by a subtropical climate, low dengue virus incidence, and negligible pre-existing immunity. To estimate the effectiveness of insecticide spraying on dengue virus containment. | NRCT | Ultra-low volume insecticide spraying. | Mortality of Ae. aegypti mosquitoes and symptomatic dengue cases | We estimated induced mortality of 40% for mosquitoes and found that the implemented control protocol avoided about 24% of symptomatic cases in the area throughout the 2015–2016 epidemic period. |
| 32 | Brazil | González, 2020 | To assess the impact of two sand fly insecticide interventions (insecticide spraying and insecticide-impregnated dog collars) on the peridomestic abundance and distribution of mosquitoes (Culicidae) and biting midges (Ceratopogonidae). | RCT | Application of insecticide: (i) pheromone + insecticide (PI group), and (ii) deltamethrin. | Adult mosquitoes | Analysis of mosquito abundances revealed a significant reduction (56%) in the PI group (IRR = 0.54, 95% CI: 0.30–0.97, *P* = 0.04). The PI intervention significantly reduced the abundance of mosquitoes inside houses (41%) and at chicken roosting sites (48%). |
| 33 | Brazil | Gesto, 2021 | To evaluate Wolbachia’s ability to invade mosquito populations and investigate the bacterium density level and the vector competence for zika and dengue virus in post-release field samples, contributing to a better characterisation of targeted populations in Southeastern Brazil. | NRCT | Release of *Wolbachia*-infected eggs. | Saliva samples from orally infected (Wolbachia+) *Ae.* mosquitoes | After the release of *Wolbachia*-infected eggs, a successful invasion and long-term establishment of the bacterium across the territory was observed. The refractoriness to dengue and Zika viruses, either thorough oral-feeding or intra-thoracic saliva challenging assays, was maintained over the adaptation to the natural environment. |
| 34 | Colombia | Romero-Vivas, 2002 | To study the effectiveness of simple netted lids to prevent oviposition. | NRCT | Cover of the most productive breeding sites of *Ae. aegypti*. | The number of larvae and pupae of *Ae. aegypti* | During the trial, 56% of inspected containers had netted lids correctly in place. Of these, 78% had no mosquito larvae. Only 37% of uncovered containers were free of mosquito larvae, a significant difference was demonstrated when these inexpensive mechanical barriers were used (P < 0.001). |
| 35 | Colombia | Luna, 2004 | To explain how qualitative and quantitative research, including formative research, and data analysis based on the Stages of Change Model, was used as the basis for planning of an integrated social mobilisation and communication approach. | Mixed method study | Health education campaign focussed on community mobilisation. | KAP, and house index | Twenty-seven percent of the people knew about and practiced specific actions to look for and control *Ae. aegypti* breeding sites. Immature forms of *Ae. aegypti* was fewer in the post-intervention evaluation compared to the pre-intervention survey. The house index decreased from 18% in 1998 to 5% in 2003. |
| 36 | Colombia | Ocampo, 2009 | To evaluate two control methods for *Ae. aegypti* that can be used by the community: Lethal ovitraps and Bti briquettes. | NRCT | The interventions were performed (i) lethal ovitraps, (ii) Bti, and (iii) lethal ovitraps in combination with Bti and education. | House index, pupal index, adult index, and knowledge | The interventions did not achieve significant differences in vector abundance among the treatments. The interventions achieved a significant reduction in entomological indices compared with those observed during the pre-intervention survey: house index 15.1% vs 8.5%, mean pupae per house 1.15 vs 0.073, and adult index 56.3% vs 34.8% (p < 0.05). |
| 37 | Colombia | Cáceres-Manrique, 2010** | To determine the efficacy of social mobilisation for its role in public empowerment in the improvement of dengue control measures. | NRCT | Health education focusing on community mobilisation. | KAP, *Ae. aegypti* infestation levels and cases of dengue | Significant differences between the control and the intervention group were observed in aspects relevant to control, such as knowledge about the disease and the characteristics of the vector, prevention, and control practices. The difference in the prevalence of dengue one year after initiation of the intervention was not significant 4.8% in the intervention group and 6.7% in control (χ2=3.4, p=0.065). |
| 38 | Colombia | Pacheco-Coral, 2010 | To estimate the impact of the information, education, and communication strategy on *Ae. aegypti* infestation in homes in La Dorada, Colombia. | Cross-sectional study | Health education campaign, with mass media communication. | The presence of immature forms of *Ae*. and adult mosquitoes | Almost 80% of the interviewees acquired knowledge about dengue transmission through the strategy. No immature forms were found in houses where somebody washed the water tank at least once a week. There were also no larvae in homes where people had knowledge about larvae and dengue transmission. |
| 39 | Colombia | Vesga-Gómez, 2010** | To evaluate the effectiveness of primary-school ’children’s play-based education for improving knowledge about dengue prevention, control and practice. | Pre-post (before-after) study | Health education campaign at school. | KAP | Significant increases in knowledge about dengue were observed. The children fulfilled their commitment and creatively and inventively engaged in more activities. |
| 40 | Colombia | Carabalí, 2013** | To evaluate the coverage and reach of an intervention based on mass-media communication of dengue surveillance reports and its effect on the presence of intra-domiciliary breeding sites for *Ae.* in Guadalajara de Buga, Colombia. | Cross-sectional study | Mass dissemination of surveillance results. | House index, the coverage, and reach of the intervention | The house index was 2.5%; coverage was 59.4% and reach was 22.3%. There was no association between the intervention and the presence of intra-domiciliary mosquito breeding sites. |
| 41 | Colombia | Alarcón, 2014** | To evaluate the impact of ovitraps loaded with Bti on traditional indexes as strategies for surveillance and control of *Ae. aegypti.* | NRCT | Usage of ovitraps with Bti. | Breteau index, house index, and container index | A total of 501,425 eggs were collected. In Apartadó, significant differences were observed in the house, container and Breteau indexes. |
| 42 | Colombia | Quimbayo, 2014** | To determine the most efficient type of lethal ovitrap for *Ae. aegypti* vector control by combining different types of insecticides, oviposition substrates, and attractant infusions. | NRCT | The usage of lethal ovitraps and insecticides (deltamethrin and permethrin). | The number of *Ae.* eggs and larvae | In the field conditions, the ovitraps with the highest vector reduction were those combining deltamethrin/towel/10% hay infusion. |
| 43 | Colombia | Ocampo, 2014 | To identify and continuously control the most productive *Ae.* (Stegomyia) breeding site in an endemic urban area in Colombia and followed the subsequent incidence of dengue. | NRCT | The intervention targeted only the approximately 4800 catch basins. Each catch basin was treated with 2 g of pyriproxyfen (±0.05 mg/mL). | Percentage of positive catch basins, house index, container index, Breteau index, pupae per person index, and incidence of dengue | Street catch basins were the potential breeding site most frequently found containing *Ae.* immature stages. Due to the high resistance to temephos, the intervention consisted of monthly application of pyriproxyfen in street catch basins. A significant decrease in catch basins positivity for *Ae.* larvae was observed after each monthly treatment (p < 0.001). Over the intervention period, a reduction in the dengue incidence was observed (rate ratio 0.19, 95% CI 0.12–0.30, p < 0.0001) after adjusting for autocorrelation and controlling with a neighbouring town. |
| 44 | Colombia | Escudero-Támara, 2015** | To evaluate the effectiveness of an educational intervention to induce changes in behaviour to eliminate breeding places for the dengue vector in families from a community in the Municipality of Sincelejo, Colombia. | Pre-post (before-after) study | Health education. | KAP and presence mosquito breeding sites | After the intervention changes were made in the levels of inadequate knowledge about dengue and behaviour of the vector from 14.8% to 3.7% (p=0.109), in their inadequate beliefs from 20.4% to 5.6% (p=0.008) and in adequate practices from 24 to 87% (p=0.001). Of the participant groups, 64.8% were classified in the action stage. There was a reduction of the number of intradomicile *Ae.* breeding places from 92.6% to 35.2% (p=0.001). |
| 45 | Colombia | Quintero, 2015 | To determine the effectiveness of long-lasting insecticide-treated net window and door curtains alone or in combination with long-lasting insecticide-treated net water container covers in reducing dengue vector density, and feasibility of the intervention. | CRT | The intervention delivered first, long-lasting insecticide-treated net curtains and secondly, water container covers to the intervention cluster. | Pupae per person index, Breteau index and container index | The Breteau index fell from 14 to 6 in the intervention group and from 8 to 5 in the control group. The additional intervention with long-lasting insecticide-treated net covers for water containers showed a significant reduction in pupae per person index (p=0.01). In the intervention group, the pupae per person index showed a clear decline of 71% compared with 25% in the control group. |
| 46 | Colombia | Vargas, 2015** | To determine if a set of integrated interventions, implemented in rural educational institutions in the municipality of Apulo, Cundinamarca, improved the quality of water for consumption and reduced mosquito vector infestation, reducing risk factors for diarrhoea and dengue diseases. | NRCT | Health education campaign at school, focussed on covering mosquito breeding sites and management of solid waste. | Breteau index, school index, container index, the pupa per person index, adult mosquito density and number of episodes and days missed from school due to dengue | Infestation rates in schools by immature forms of *Ae. aegypti* and of pupa/person were less in the post-intervention stage, being this last one statistically significant. No impact was achieved on the adult population of *Ae. aegypti.* |
| 47 | Colombia | Criado Morales, 2016** | To implement community participation as a methodology for preventing dengue. | Pre-post (before-after) study | Health education campaign. | KAP, Breteau index, house index, and container index | Knowledge related to dengue increased by 4,5 %. Breteau index was zero in two of the six districts. Three districts did not exceed 5%. |
| 48 | Colombia | Overgaard,  2016 | To investigate whether interventions targeting diarrhoea and dengue risk factors would significantly reduce absence due to diarrheal disease and dengue entomological risk factors in schools. | CRT | Health education campaign at schools. | Adult female *Ae. aegypti* density, Breteau index, school index, container index, pupae per person, the proportion of schools with adult female Ae. aegypti (%) | Interventions had no apparent effect on adult female *Ae. aegypti* density (p = 0.32). However, the dengue interventions reduced the Breteau index on average by 78% (p = 0.029), with Breteau indices of 10.8 and 6.2 in the “dengue” education group and “dengue and diarrheal” education group, respectively compared to 37.5 and 46.9 in the “diarrheal” education group and control group respectively. |
| 49 | Colombia | Jaramillo, 2018** | To evaluate the sustainability of a set of interventions to prevent dengue and diarrhea in 33 rural schools in the municipalities (counties) of Anapoima and La Mesa, Colombia, two years post-project. | NRCT | Health education campaign at schools. | KAP, mosquito breeding sites, and sustainability of the intervention | The total sustainability score for dengue prevention was unsustainable in all arms except for the health education focussed on dengue only, which had a moderate level. Maintenance of benefits and interventions was moderately sustainable, while capacity development and institutionalisation were not sustainable. The differences between the four arms were not statistically significant. The KAP of the students showed greater sustainability compared to the absence of potential breeding sites for *Ae. aegypti* in schools, with no statistically significant differences between the intervention arms. |
| 50 | Colombia | Ceron-Hernandez, 2020 | To evaluate the physical modification of storm drains as a control measure of the dengue vector *Ae. Aegypti.* | RCT | Installation of a filter bed that prevents water retention after a rain event (> 100 mm). | The presence of standing water in the storm drains and the number of *Ae. aegypti* larvae | Of the 21 modified storm drains, 5 contained standing water, and 19 of the 21 unmodified storm drains contained standing water. The average number of larvae per storm drain, was 3.2 in the modified ones and 31.9 in the unmodified ones (ratio of 0.10). Using negative binomial regression, the 95 % confidence interval of the 0.10 ratio is 0.014 to 0.74 (a reduction between 98.6 and 26 %), with a *p-value* of 0.016. |
| 51 | Colombia | Quintero, 2020 | To present the impact of an *Ae.* vector control intervention “Girardot Aedes-free” in reducing the number of reported dengue cases in Girardot, Colombia, between 2015 and 2017 | NRCT | Health education campaign based on an eco health approach, focusing on community mobilisation. House inspection, covering water containers with insecticide-treated aluminium covers, focussing on the most productive containers. | Incidence of dengue | The analysis indicates that the intervention resulted in a decrease of an average of between 0.12 (-0.25,0.01) and 0.26 (-0.42, -0.10) cases of dengue daily (1.82 cases per week or 7.8 cases per month or 95 cases per year) in Girardo |
| 52 | Costa Rica | Perich, 2003 | To evaluate the efficacy of the insecticide formulation applied as either an ultralow volume or thermal fog spray application.  To evaluate the effectiveness of low volume application of lambda-cyhalothrin in suppressing *Ae. aegypti* populations in Costa Rica. | NRCT | Application of insecticides: (i) ultra-low volume at the front door, (ii) ultra-low volume in each room, (iii) thermal fog at the front door, (iv) thermal fog in each room, (v) low volume at the front door, or (vi) low volume in each room. | Mortality of adult mosquitoes | Sentinel caged mosquito mortality in open and sequestered locations was 97-100% for the ultra-low volume and thermal fog spray treatments, with control mortality less than 2%. Both ultra-low volume applications (front door and each room) provided 3 weeks of significant control (P < 0.05) based on adult *Ae. aegypti* house collections. |
| 53 | Costa Rica | Rodríguez, 2009** | To analyse the behaviour of the entomological indexes before and after the control interventions on the vector and identify the breeding sites of *Ae. aegypti.* | Pre-post (before-after) study | Garbage collection, source reduction, usage of abate as larvicide and heat treatment for adult vectors inside and outside the house. | Breteau index, house index and container index | The first survey showed house index values over 5 in 12 areas, whereas 5 exhibited increased values after the interventions during the second survey. Positivity percentages in swampy places were low, 3.6 and 2.9% before and after the interventions, respectively. |
| 54 | Ecuador | Mitchell-Foster, 2015 | To investigate the effectiveness and feasibility of scaling-up an eco-bio-social approach for implementing an integrated community-based approach for dengue prevention in comparison with existing insecticide-based and emerging biolarvicide-based programs in an endemic setting in Machala, Ecuador. | CRT | An integrated intervention strategy for dengue prevention, including a health education campaign at school and a clean patio and safe container program | Pupae per person index, house index, Breateu index, and KAP | The integrated intervention strategy was successful in reducing pupae per person index levels in intervention clusters versus control clusters, with the six paired clusters that followed the study design experiencing a greater reduction of pupae per person index compared to controls (2.2 OR, 95% CI: 1.2 to 4.7). A reduction in both house index and Breateu index at the houses of children that participated in the school program was observed. |
| 55 | Guatemala | Rizzo, 2012 | To test the efficacy, cost and feasibility of a combined approach of insecticide treated materials alone and in combination with appropriate targeted interventions of the most productive vector breeding-sites. | CRT | Two interventions were performed. The first intervention (coverage of window and exterior doorways made of PermaNet 2.0 netting, factory treated with deltamethrin at 55 mg/m^2^, and of 200 L drums with similar treated material). The second intervention (combination of treated materials and other interventions targeting productive breeding-sites i.e larviciding with temephos, elimination etc.) | Total production of *Ae.* pupae, pupae per person index, house index, container, Breateu index, the coverage, people’s acceptance, and cost of the intervention | After covering 100% of windows and exterior doorways and a small number of drums in 970 households, tropical rains occurred in the area and lead to an increase of the vector population, more pronounced (but statistically not significant) in the control arm than in the intervention arm. In the second intervention the combined approach of insecticide treated materials and interventions against productive containers lead to significant differences on reductions of the total number of pupae (P = 0.04) and the house index (P = 0.01) between intervention and control clusters, and to borderline differences on reductions of the pupae per person and Breteau indices (P = 0.05). The acceptance of the intervention was generally high, particularly in families who had experienced dengue. |
| 56 | Guatemala | Ulibarri, 2016 | To study the effectiveness of an integrated intervention of health worker training, a low-cost ecological mosquito ovitrap, and community engagement on *Ae.* spp. mosquito control over 10 months in 2015 in an urban remote community in Guatemala at risk of dengue, chikungunya and Zika virus transmission. | CRT | A three-component integrated intervention consisted of: (i) web-based training of local health personnel in vector control, (ii) cluster-randomised assignment of ecological ovillantas or standard ovitraps to capture *Ae aegypti* mosquitoes, and (iii) community engagement to promote participation of community members and health personnel in the understanding and maintenance of ovitraps for mosquito control. | Ae. mosquito eggs, KAP of the community and the health workers | When ovillantas were used, significantly more eggs were trapped by ecological ovillantas than standard ovitraps over the 10-month study period (t=5.2577; p<0.05). The mean weekly egg count was higher in neighbourhoods with ovillantas with a mean of 19.26334 (SE 0.4707; 95% CI: 18.34056, 20.18613) than at the control sites using standard ovitraps, with a mean of 13.2787 (SE 0.8249; 95% CI: 11.66214, 14.89748). The difference was statistically significant (t= 5.2577; p< 0.05). Among both community members and health workers, the levels of KAP increased. |
| 57 | Honduras | Avila Montes, 2004** | To evaluate the effectiveness of a special course on environmental health and dengue, given to primary school students and intended to promote behavioural changes in the mothers of those students that would lead to the safe handling of water, adequate disposal of trash, and control of household breeding sites of *Ae. aegypti*, the mosquito vector for dengue. | NRCT | Health education campaign at school | KAP, house index, Breteau index, and container index | In the two intervention schools, there was a significant increase in the students’ knowledge of the following three variables: dengue caused by a virus, life cycle of the vector, and reduction of breeding sites, (P < 0.0001 for all those increases in knowledge). There were also significant increases in the knowledge of the same variables among the teachers in the intervention schools. There was a statistically significant difference in the Breteau index values between the two control group schools and the one intervention school where the education course was implemented more adequately. |
| 58 | Honduras | Avila Montes, 2012** | To determine and assess the results obtained and the lessons learned from a health education program at schools during the 2005–2010 period. | Pre-post (before-after) study | Health education campaign at school | House index, reservoir index, and Breteau index | A reduction of the larval index was observed. The intervention promoted community participation in environmental issues, emphasising *Ae*. control. |
| 59 | Mexico | Espinoza-Gómez, 2002 | To evaluate the effect of an educational campaign for reducing the breeding places of *Ae. aegypti*, the principal vector of dengue; and to compare its effects with the ones obtained by spraying of Malathion at ultra-low volume. | CRT | The interventions were (i) an educational campaign, (ii) malathion spraying at ultra-low volume, (iii) both treatments simultaneously | The number of positive containers with larva by house | The average of the positive containers by house was reduced from 0.97 to 0.77. A two-way analysis of variance showed that this reduction was more apparent in the houses that received educational campaign (*F*=8.4, p<0.005) with relation to the ones that received malathion spraying (*F*=0.38, p>0.5), while the combination of both treatments demonstrated a discrete negative interaction (*F*=6.52, p<0.05). |
| 60 | Mexico | Galván, 2004 | To reflects on the use of ongoing formative research to identify and test appropriate household-based control methods for key *Ae. aegypti*-producing containers and the  creation of an education/communication strategy for the dissemination of highly specific messages for the key containers. | Pre-post (before-after) study | Health education campaign | KAP, house index, container index, and Breteau index | In general, a decline was seen post-intervention across the house index, container index, and Breteau index. |
| 61 | Mexico | Marina, 2012 | To evaluate the efficacy of Spinosad as a larvicide in car tire habitats. | NRCT | Application of spinosad (1 ppm), spinosad (5 ppm), 0.4 g 1% temephos granules, and 50 μl Vectobac AS12 (Bti) | Numbers of *Ae.* spp. eggs, larvae, and pupae | Spinosad treatments at 1 or 5 ppm provided 6–8 weeks of effective control of *Ae. aegypti,* and *Ae. albopictus* both in the dry season and the rainy season. The larvicidal performance of VectoBac 12AS was relatively poor with one week of complete control of *Ae.* spp. larvae. The duration of larvicidal activity of 1% temephos granules was intermediate between those of VectoBac and Spinosad treatments. |
| 62 | Mexico | Martínez-Ibarra, 2012 | To evaluate the effects of a program aimed at very young children to control domiciliary *Ae. aegypti* populations in four neighbourhood districts of a medium sized city in Jalisco, western Mexico | NRCT | Health education at school | KAP, container index, and house index | The entomological indices decreased significantly (P<0.05) in houses in the intervention area, apparently because parents acted on the comments and suggestions of the children and eliminated or monitored mosquito breeding sites. |
| 63 | Mexico | Anguiano- Moreno, 2013** | To contribute to the control of dengue in the western central region of Mexico through risk reduction, maintain epidemiological control of dengue and prevent deaths from this cause in the state of Colima, through innovative strategies and efficient: social participation, prevention measures, and control, social communication, operational research, monitoring, and evaluation. | Record-surveillance | The interventions: (i) social participation, (ii) three cycles of space spraying with phenothrin with heavy utra-low volume machines, (iii) house visit and treatment with temephos, (iv) chemical and physical control in abandoned houses, (v) a communication strategy “patio and the roof clean”, (vi) dengue cases derived from high migration to the municipalities of Armería, Manzanillo, and Tecomán were monitored, and (vii) the kiosks participated in the intervention by selling Abate to the community | Incidence of dengue, house index, container index, and Breteau index | Dengue incidence decreased  from 81.4% in 2010 to 79.1% in 2011. The positive house index was at 0.5% in 2010 and 0.6% in 2011, the positive container 0.2% in both years and Breteau by 0.7% in 2010 and 0.8% in 2011. The interventions reduced the peak of cases that had been recorded in the rainy season resulting from the transmission of dengue. |
| 64 | Mexico | Loroño-Pino, 2013 | To determine the potential to reduce dengue virus transmission through insecticide-treated curtains use in individual homes. | CRT | Usage of insecticide-treated (deltamethrin) curtains | Mosquito abundance, incidence of dengue in humans and *Ae. aegypti,* mosquito | Overall, insecticide-treated curtains reduced intradomicile dengue virus transmission. Homes with insecticide-treated curtains homes were significantly less likely to experience multiple dengue virus infections in humans than homes without insecticide-treated curtains. Dengue virus–infected *Ae. aegypti* females were reduced within the homes with insecticide-treated curtains. |
| 65 | Mexico | Torres, 2014** | To identify dengue-related knowledge, attitudes, and practices among primary school students in Tapachula, Chiapas, Mexico, before and after an educational intervention. | Pre-post (before-after) study | Health education campaign at school | KAP | The students’ level of knowledge was significantly higher after the implementation of the health education campaign. In comparison with the fifth-graders, the sixth-grade students both already had and also acquired significantly more knowledge of several aspects of the disease and the vector. |
| 66 | Mexico | Che-Mendoza, 2015 | To assess the long-term (> 2 years) impact of long-lasting insecticide-treated house screens  and targeted treatment  in controlling domestic *Ae. aegypti* infestations, when deployed simultaneously, in an urban environment with high dengue transmission in Mexico. | CRT | Long-lasting insecticidal net screens fitted to domestic windows and doors in combination with targeted treatment of the most productive *Ae. aegypti* breeding sites | Breteau index, container index, house index, and pupae per person index | Long-lasting insecticidal net screens clusters had significantly lower infestations compared to control clusters at 5 and 12 months after installation, as measured by adult and pupal-based vector indices. After the addition of targeted treatment to the intervention houses in intervention clusters, indices remained significantly lower in the treated clusters until 18 (immature and adult stage indices) and 24 months (adult indices only) after the intervention. |
| 67 | Mexico | Manrique-Saide, 2015 | To investigate the use of insecticide-treated screens permanently affixed to windows and doors in Mexico. | CRT | Usage of screens (Duranet, Clarke Mosquito Control) | House infestation and infestation density for *Ae. aegypti* mosquitoes | At 5 months post intervention, significantly fewer treated than control houses were infested with *Ae. aegypti* adult female mosquitoes (OR 0.38, 95% CI 0.21–0.69), blood-fed females (OR 0.36, 95% CI 0.21–0.60), and males (OR 0.39, 95% CI 0.19–0.77). A significant effect was still seen at 12 months for adult females and males but not for blood-fed females. Analyses of infestation density showed similar trends, with significantly fewer *Ae. aegypti* mosquitoes found in treated than in control houses. |
| 68 | Mexico | Tavor-Zamora, 2015 | To evaluate the effectiveness of chemical control of larvae of *Ae. aegypti* using different indices before and after application at three locations in Baja California Sur, Mexico. | Record-surveillance | The intervention: epidemiological surveillance, medical care for ill patients, and environmental sanitation through social mobilisation, which involves physical management (elimination, removal, and protection) of artificial containers and chemical control (application of granular temephos larvicide in water containers or spraying insecticide in the environment) | House index, container index, and Breteau index | Significant statistical differences (p < 0.001) were observed in the relative abundance of *Ae. aegypti* larvae before and after applying larvicide temephos and by removing water containers where mosquitoes reproduce. In La Paz, the average of the house index was reduced from 16- 83% to 0-5% after control during the 3 years. In Cabo San Lucas, the value was reduced from 2-40% to <1% and in San Jose del Cabo such variation was 4-46% to 0-7%. The index of positive containers before and after management varied in La Paz from 6-34% to 0-4.7%; in Cabo San Lucas from 0.2-23% to <1%, and in San Jose del Cabo from 0.6-11% to <1.2%. The Breteau index in La Paz varied from 48-358% to 0-12.5%, in Cabo San Lucas, values decreased from 3-67% to <1%, and in San Jose del Cabo from 3-174% to 0-16% after the intervention. |
| 69 | Mexico | Hernandez-Suarez, 2016 | To analyse the effect of reducing transmission in elementary schools (grades 1–9) on the dynamics of the epidemic at a regional level. | Record-surveillance | Health education campaign focused on training janitors to locate and avoid mosquitoes’ breeding places, to maintain elementary schools free of mosquitoes | Incidence of dengue | By the end of 2007, a reduction in dengue infection rates was observed right at the beginning of the school year compared to the previous year. By week 35, 2 weeks after the beginning of classes, the infection rate (per capita) in 2007 was 0.03, compared to 0.26 in 2006. |
| 70 | Mexico | Jiménez-Alejo, 2017 | To assess the impact of the community action on pupal production of the dengue vector *Ae. aegypti* in both rainy and dry seasons. | CRT | Health education campaign focused on community mobilisation for control of *Ae. aegypti* breeding sites and in addition to continuing normal prevention efforts, such as application of temephos to HHs water containers | Pupae per person, pupae per household, container index, household index, and Breteau index | All entomological indices were lower in the intervention clusters than in control clusters in both the rainy season and the dry season. Pupae per household 0.46 (intervention group) and 0.82 (control group) in rainy season, and 0.41 (intervention group) and 0.83 (control group) in dry season. Household index 16% in the intervention group and 21% in the control group in the rainy season, and 12.1% in the intervention group and 17.9% in the control group in the dry season. Breteau index 27% (intervention group) and 36% (control group) in rainy season, and 19% (intervention group) and 29% (control group) in dry season. All differences between the intervention and control clusters were statistically significant. |
| 71 | Mexico | Morales-Pérez, 2017 | To assess whether the use of fish is associated with a reduction in dengue virus infection. | Cross-sectional study | The usage of fish in water containers | Container index and incidence of dengue | The presence of fish was associated with lower levels of recent dengue virus infection in children aged 3–9 years (OR 0.64; 95% CI 0.45–0.91). |
| 72 | Mexico | Che-Mendoza, 2018 | To evaluate the entomological impact of the insecticide-treated screening intervention on indoor  *Ae. aegypti* populations in the city of Merida, Mexico. | CRT | The usage of pyrethroid-impregnated long-lasting insecticide-treated netting | House positivity for adult female Ae. aegypti, house positivity for any Ae. aegypti adults, number of female Ae. aegypti per house, and the number of total Ae. aegyptiper house | Significant reductions in the indoor presence and abundance of *Ae. aegypti* adults (OR = 0.48 and IRR = 0.45, P<0.05 respectively) and the indoor presence and abundance of Ae. aegypti female mosquitoes (OR = 0.47 and IRR = 0.44, P<0.05 respectively) were detected in intervention clusters compared to controls. |
| 73 | Mexico | Loroño-Pino, 2018 | To investigate the potential for a Casa Segura approach to control *Ae. aegypti* and dengue virus transmission in fraccionamiento style housing in urban/suburban and rural sites in Merida and surrounding towns in Yucat State, Mexico. | CRT | The usage of insecticide-treated curtains | Adult mosquitoes density, dengue virus infections in*Ae. Aegypti* and humans | Insecticide-treated curtains reduced the indoor abundance of*Ae. aegypti* and the number of dengue virus-infected mosquitoes in homes in rural but not in urban/suburban study areas*.*The presence of non-treated screens also was associated with reduced numbers of mosquitoes in homes. |
| 74 | Mexico | Newton-Sánchez, 2020 | To analyse the effect of a community participation programme based on the ecosystem model on the incidence of dengue in urban communities. | RCT | Health education campaigns: community participation focusing on the ecosystem | The incidence of dengue and Breteau index | The incidence of dengue in the intervention group was 2.58%/month (n = 818) and in control group 2.26%/month (n = 994), with a risk ratio of 1.14 (95% CI 0.89-1.45) and the population attributable fraction of 0.06 (95% CI - 0.056 to 0.16). The Breteau Index was reduced in the intervention group. |
| 75 | Mexico | Devine, 2021 | To report the findings of a randomised field trial evaluating the entomological impact of passive emanators containing the volatile pyrethroid metofluthrin against urban *Ae. aegypti* in the state of Yucatan, Mexico | RCT | Usage of the metofluthrin emanators | Indoor *Ae. aegypti* adult abundance, female abundance, blood-fed abundance, and estimates of *Ae. aegypti* landing behaviour | After metofluthrin emanators installation, the entomological indices between the trial arms diverged. There were significant reductions in Abundance Rate Ratios for total *Ae. aegypti*, female abundance, and females that contained blood meals (2.5, 2.4, and 2.3-times fewer mosquitoes respectively; P<0.001). |
| 76 | Mexico | Manrique-Saide, 2021 | To evaluate the efficacy of protecting houses with the insecticide-treated screening on Ae. aegypti infestation and arbovirus infection during a Zika outbreak in Merida, Yucatan, Mexico. | CRT | Usage of insecticide-treated screening. Both the intervention and the control clusters received routine control | Indoor adult mosquito infestation and arbovirus infection among mosquitoes | Houses with insecticide-treated screening were 79–85% less infested with Ae. Females mosquito than control houses for up to one year. Houses with insecticide-treated screening had significantly fewer infected female Ae. aegypti than controls during the peak of the epidemic (OR = 0.15, 95%CI: 0.08–0.29). |
| 77 | Mexico and Venezuela | Kroeger, 2006 | To measure the impact on the dengue vector population (*Ae. aegypti*) and disease transmission (dengue) of window curtains and water container covers treated with insecticide. | CRT | Insecticide-treated (lambdacyhalothrin or deltamethrin) curtains and water treated with pyriproxyfen chips or covered | Breteau index, house index, pupae per person index, container index, and IgM serology | In both study sites, entomological indices at the end of the trial were significantly lower than those at baseline, though with no significant differences between control and intervention arms. Prevalence of IgM measured at baseline was 16% and 21% in intervention and control clusters, respectively. After eight months, this had dropped to 8% in the intervention clusters but had not changed significantly in the control groups 18%. |
| 78 | Nicaragua and Mexico | Andersson, 2015 | To test whether community mobilisation adds effectiveness to conventional dengue control. | CRT | Health education campaign focussed on community mobilisation | Incidence of dengue, house index, container index, Breteau index and pupae per person | Serological evidence from intervention sites showed a lower risk of infection with dengue virus in children (relative risk reduction 29.5%, 95% confidence interval 3.8% to 55.3%), fewer reports of dengue illness (24.7%, 1.8% to 51.2%), fewer houses with larvae or pupae among houses visited (house index) (44.1%, 13.6% to 74.7%), fewer containers with larvae or pupae among containers examined (container index) (36.7%, 24.5% to 44.8%), fewer containers with larvae or pupae among houses visited (Breteau index) (35.1%, 16.7% to 55.5%), and fewer pupae per person (51.7%, 36.2% to 76.1%). Temephos in household water containers was associated with higher levels of serological evidence of dengue infection. |
| 79 | Nicaragua | Arostegui, 2017 | To examine potentially protective effects of temephos on entomological indices under everyday conditions of the national vector control program. | Record-surveillance | The dengue control program of the Ministry of Health carries out 4–6 cycles of temephos abatement annually in all municipalities of Managua. In addition, the government program conducts spatial fumigation and educational activities about the elimination of *Ae.* breeding sites | Household Index, households with pupae, and pupae per person | Between 2005 and 2013, Temephos exposure was not significantly associated with a reduction in any of the three mentioned entomological indices. In six of 18 multivariate models at the six time points, temephos exposure was associated with higher entomological indices. |
| 80 | Paraguay | Parra, 2020 | To test whether community mobilisation reduces infestation levels. | NRCT | Health education campaign focusing on community mobilisation | House index | In July 2018, the most significant decrease was observed in the intervention group, with indices at 2.61% (intervention,-19.91%) and 9.87% (control, -5.73%). In April 2019, with half-flooded territories, the house index of both the intervention and control groups increased, but the increase was lessened in the intervention group. |
| 81 | Peru | Machaca, 2002** | To show the results and impact achieved in the control and/ or possible eradication of dengue from the jurisdiction of Sechura. | Record-surveillance | Scheduled and periodic container washing campaigns | *Ae.*,container and Breteau index | The *Ae.* (larvae) index found at the beginning of the study (46%) decreased to 3.3% in 20 days. After the second data collection method till 1-12-2001, the larvae, container, and Breteau remained close to 0. |
| 82 | Peru | Dammert, 2014 | To determine the effectiveness of mobile phone technology in improving households’ health preventive behaviour in dengue-endemic area. | CRT | Health education campaign based on mass media communication | Health-preventive behaviour, house index, container index and Breteau index | The results suggest that repeated exposure to health information encourages HHs’ uptake of preventive measures against dengue. With regard to framing effects, we do not find statistically significant differences between interventions, except for one outcome (the non-monetary message compared to the control group). The results imply that HHs in the intervention group experienced a decreased in the percentage of water-holding containers testing positive for dengue larvae (1.44% vs. 2.47% in the intervention and control groups) as well as the number of positive water-holding containers per household (10.66 vs. 18.91 in the intervention and control groups). |
| 83 | Peru | Paredes-Esquivel, 2016 | To investigate the efficacy of deltamethrin S.C. applied through indoor residual spraying of dwellings in reducing *Ae. aegypti* populations. | NRCT | Application of insecticide: deltamethrin applied through residual indoor spraying | Breteau index, house index, and container index | The results showed that in an area with moderate levels of *Ae. aegypti* infestation, residual indoor spraying dramatically reduced all immature indices the first week after treatment and the adult index from 18.5 to 3.1, four weeks after treatment (p < 0.05). Even though housing conditions facilitated reinfestation with *Ae. aegypti* indices remained low compared to baseline and 16 weeks after insecticide application. |
| 84 | Peru | Gunning, 2018 | To evaluate the impact of 6 cycles of indoor ultra-low volume pyrethroid spray applications on *Ae. aegypti* populations. | NRCT | Application of ultra-low volume pyrethroid spraying | *Ae. aegypti* populatio density | Trail 1: Spraying reduced the mean number of adults captured per house by ±83 percent relative to the pre-spray baseline survey.  Trail 2: The number of adults per house in the intervention area was reduced ±64 percent relative to baseline. |
| 85 | Peru | Lenhart, 2020 | To quantify the impact of insecticide-treated curtains  on dengue virus seroconversion as measured through plaque-reduction neutralisation tests. | CRT | Usage of insecticide-treated curtains | Dengue seroconversion, adult and immature Ae. aegypti abundance | Seroconversion data showed that individuals living in the intervention cluster were at greater risk of seroconverting to DENV, with an average seroconversion rate of 50.6 per 100 person-years (PY) (CI: 29.9–71.9), while those in the control arm had an average seroconversion rate of 37.4 per 100 PY (CI: 15.2–51.7). Entomological indicators did not show statistically significant differences between intervention and control clusters. |
| 86 | Uruguay | Basso, 2015 | To implement and evaluate innovative interventions that increase the effectiveness of institutions working on dengue prevention (Ministry of Public Health), and to encourage participation and empowerment of citizens to generate appropriate, sustainable recommendations. | CRT | Ecosystem management measures consisted of promoting and organising a campaign together with community members and public health institutions for the physical or functional removal of containers in and around their homes. The HHs received a plastic bag for collecting small unused water containers. Large tanks were mapped and covered. | Breteau index, container index, house index, pupae per person index, pupae/ hectare index, and cost analysis | The number of containers accounted for in the HHs after the intervention diminished 47.4% when compared with the number of containers registered in the baseline survey. When comparing the increase from spring to autumn the vector densities in intervention clusters on average increased less than those in the control clusters, although the difference was statistically not significant. |
| 87 | Uruguay | Basso, 2017 | To test if the distributions of plastic bags for all HHs to collect all discarded water containers reduce the number of pupae per person index. | CRT | Distribution of plastic bags for collecting unused small containers. Health education focussed on community mobilisation. | Pupae per person index, house index, container index, Breteau index, and community involvement | The average pupae per person index, decreased in the intervention clusters 11 times and in the control clusters only four times (P < 0.05). The container index, house index, and Breteau index decreased in the intervention clusters more than those in the control clusters, although the difference was statistically not significant. |
| 88 | Venezuela | Vivas, 2003** | To assess the value of a game known as “Jugando en salud: dengue” as a teaching/ learning tool for schoolchildren to control of *Ae aegypti* and prevent dengue in the municipality of Girardot, Aragua state, Venezuela. | NRCT | Health education campaign at school | Knowledge and skills to control *Ae.* mosquito | The knowledge about dengue and the skills that were measured prior to following the prescribed program were lower (6.5 and 18.4 points, respectively) than those displayed in the final test (8.25 and 22.9 points, respectively; P < 0,05). Students that received the intervention also appeared to acquire more skills than those in the control group (P < 0,05). |
| 89 | Venezuela, Mexico, Peru and other countries. | Tun-Lin, 2009 | To test the non-inferiority hypothesis that a vector control approach targeting only the most productive water container types gives the same or greater reduction of the vector population as a non-targeted approach in different ecological settings and to analyse whether the targeted intervention is less costly. | CRT | Venezuela: cover drums with insecticide treated nets.  Mexico: buckets and pot management.  Peru: source reduction and treat water with pyriproxyfen. | Breteau index and pupae per person index | Difference in reduction for intervention group vs. control group was calculated as Breteau index and pupae per person from baseline to 5-months follow up: Peru (Breteau index: −1.05 with a 95% CI: −12.64–10.53 and pupae per person: 0.365 with a CI: −0.030–0.760). Mexico (Breteau index: −12.65 with a 95% CI: −28.77–3.47 and pupae per person −0.529 with a CI: −1.034–−0.024). Venezuela (Breteau index: 0.84 with a 95% CI: −8.94–10.62 and pupae per person: −0.023 and a 95% CI: −0.749–0.703). |
| 90 | Venezuela | Vanlerberghe, 2011 | To assess the operational effectiveness of long-lasting insecticide treated materials, when used at household level, for the control of *Ae. aegypti* in moderately infested urban and suburban areas. | CRT | Usage of insecticide treated materials, consisting of curtains and water jar-covers | Breteau index and pupae per person index | In both urban and suburban clusters, the Breteau index showed a sustained 55% decrease, while no pattern was observed at the municipal level. After controlling for confounding factors, the percentage insecticide treated curtain coverage, but not insecticide treated jar-cover coverage, was significantly associated with both entomological indices (incidence rate ratio = 0.98; 95%CI 0.97–0.99). |

**Note:**

*****Portuguese

****** Spanish

CRT: Cluster randomised trial

RCT: Randomised Control Trail

HHs: Households

KAP: Knowledge, Attitude and Practices

NRCT: Non-randomised control trail

Bti: *Bacillus thuringiensis var israeliensis*

PPM: One part per million, equivalent to one gram of active ingredient in 1 million milliliters of water.
