# Supplementary material for: Effectiveness of Aedes-borne infectious disease control in Latin America and the Caribbean region: A scoping review
Source: PLoS One. 2022 Nov 2;17(11):e0277038. doi: 10.1371/journal.pone.0277038 (PMC9629598; doi:10.1371/journal.pone.0277038)
Supplement: S4 Table — (DOCX) [file pone.0277038.s004.docx]

S4 Table Characteristics of studies identified in the Caribbean region about *Ae. aegypti* and *Ae. albopictus* prevention and control interventions

|  | **Country** | **Author, year** | **Objective (s)** | **Study design** | **Type of intervention (s)** | **Outcome (s)** | **Main results** |
| --- | --- | --- | --- | --- | --- | --- | --- |
| 1 | Cuba | Sanchez, 2005 | To document the effectiveness of a local-level intersectoral approach. | NRCT (Non-randomised controlled trial) | Communication and social mobilisation strategy to *Ae. aegypti* control. The control methods included eliminating containers, covering tanks, and cleaning public and inhabited areas. | KAP (Knowledge, attitude, practices), house index and container index | Good knowledge about breeding sites and disease symptoms increased significantly (by 49.7% and 17.1%, respectively) in the intervention area and the proportion of respondents eliminating containers in and around their houses (by 44%). The house index in the intervention area was 3.72% at baseline and decreased to 0.61% after one year. |
| 2 | Cuba | Toledo, 2007 | To achieve social mobilisation and wide community participation in *Ae. aegypti* control, we designed an intervention that built an alliance  between primary healthcare staff and the communities they serve. | NRCT | Health education campaigns focusing on community mobilisation/ participation. | Community participation, behavioural changes and entomological indicators | At the end of the study, the number of uncovered water containers had decreased by 46.7% (P < 0.01), and the number of houses with unprotected artificial containers had decreased by 55.5% (P < 0.01). There was a significant reduction in the median container indices between 2000 and 2002. A significant reduction (P < 0.01) in the median house indices per block of houses, from 1.23% to 0.35% (72% reduction) in the intervention area and from 2.08% to 0.52% (75% reduction) in the control area. |
| 3 | Cuba | Toledo Romani, 2007 | To evaluate the sustainability of the intervention strategy over a period of 2 years after the withdrawal of external support. | NRCT | Health education campaigns focusing on community mobilisation/ participation. | Maintenance of effects (entomological indices and behavioural changes), institutionalisation and maintenance of activities | In the intervention area, 87.5% of the water containers remained well covered in 2004, and 90.5% of the families continued to correctly use a larvicide, against 21.5% and 63.5%, respectively, in the control area. The house index declined from 0.35% in 2002 to 0.17% in 2004 in the intervention area, while in the control area, it increased from 0.52% to 2.25%. |
| 4 | Cuba | Sánchez, 2008** | To document the process and analyse the results of implementing a strategy aimed at increasing community participation in the fight against the dengue  mosquito vector. | Pre-post (before-after) study | Health education campaigns focusing on community mobilisation/ participation. | Community participation, larvae/ pupae index, and incidence of dengue | An increase in community participation was observed. At the end of two years of intervention, the rate of *Ae. aegypti* larvae and pupae deposits found per 100 households had declined 79%, and cases of dengue were not detected in any of the districts |
| 5 | Cuba | Toledo, 2008 | To identify key elements that should provide an added value and assure sustainable effects of the deployment of technical tools for *Ae. aegypti* control. | NRCT | The intervention combined two complementary technical interventions: (i) the distribution of new ground-level water tanks and (ii) the intensive use of an insecticide. | Community perceptions, household risk behaviour, positive containers, house index, and container index | Perceived self-efficacy to solve *Ae. aegypti* infestation and prevent dengue was not modified. No changes in behaviour were observed. The container indices decreased significantly from 0.7% before to 0.1% one month after the intervention in the study area. Six months later, they had increased to 2.7% and uncovered new water tanks constituted 75.9% of all breeding sites. Over the nine months after the intervention, the average monthly house indices were similar in the intervention and control areas. |
| 6 | Cuba | Díaz, 2009** | To describe the design, implementation and evaluation of an intersectoral strategy with an eco-health approach to prevent dengue transmission at the local level. | Pre-post (before-after) study | Participatory research for dengue control, focussing on community mobilisation. | Community participation | In the beginning, 85% of the vector sources were tanks located in the patios of the houses, and two years later, the percentage reduced to 29%. It was found that 16% of the 4,878 courtyards in the territory were not cleaned up. Two years after the end of the study, these constitute less than 1%; the number of unprotected low water tanks decreased from 62% to 8% (n = 4,678). |
| 7 | Cuba | Sanchez, 2009 | To document the process, outcome and effectiveness of a community-based intervention for dengue control. | NRCT | Intersectoral coordination intervention focusing on community empowerment. | Community involvement in decision making, execution, evaluation of dengue control activities and behaviour changes. The number of houses inspected, the number of positive containers (with *Ae. aegypti* pupae or larvae) and the Breteau index | Mean scores for participation in the pilot area were 1.6, 3.4 and 4.4 at baseline and two years after initiating intersectoral coordination and intersectoral coordination plus community empowerment interventions. 80% of HHs involved in the community empowerment intervention showed adequate behavioural patterns. Significantly lower Breteau indices were observed in the intervention areas than in the control area. |
| 8 | Cuba | Vanlerberghe, 2009 | To assess the effectiveness of an integrated community based environmental management strategy to control *Ae. aegypti*, the vector of dengue, compared with a routine strategy. | CRT (Cluster randomised trial) | Health education campaign focusing on community engagement. | House index, Breteau index and pupae per person index | At baseline, the *Ae.* infestation levels were comparable between intervention and control clusters. These indices were significantly lower in the intervention clusters at the end of the intervention: rate ratio for house indices 0.49 (95% confidence interval 0.27 to 0.88) and rate ratio for pupae per person 0.27 (0.09 to 0.76). |
| 9 | Cuba | Toledo, 2011 | To evaluate vector infestation levels and incidence and distribution of clinical dengue cases in the  original intervention and control blocks. | CRT | Community-based strategy:  (i) establishment and training of a formal task force, the community working groups. (ii) securing intersectoral coordination between the community working group and the existing local government and health structures. (iii) creation of formal links with the routine vector control programme. | Incidence of dengue and Breteau Index | During the outbreak, the attack rate of dengue fever was 8,5 per 1000 inhabitants in the intervention blocks and 38.1 per 1000 inhabitants in the control blocks, which corresponds to a relative risk of 4.5 (95% CI 3.1–6.5). There was a significantly higher proportion of unaffected in the intervention blocks, and affected blocks had fewer cases than affected control blocks. |
| 10 | Cuba | Castro, 2012 | To test the effectiveness of a community empowerment strategy intertwined with the routine dengue vector control programme in La Lisa, Cuba. | CRT | Community empowerment intervention: organisation and management, entomological risk surveillance, capacity building, and community work for vector control. | Breteau index, KAP and community participation change | The community participation score increased from 1.4 to 3.4. In the intervention and control clusters, good knowledge of breeding sites increased by 52.8% and 27.5%, respectively. Over the intervention period, the Breteau index remained 53% (95% CI 22–92%) lower in intervention clusters than in the control clusters. The empowerment strategy increased community involvement and added effectiveness to routine *Ae. aegypti* control. |
| 11 | Cuba | Sanchez, 2012 | To evaluate the results obtained through intersectoral coordination and community empowerment in one study carried out during six years in Playa Municipality, Cuba. | NRCT | Health education campaign focusing on community empowerment. | Breteau index | The Breteau index in the control area showed the lowest value before the intervention. This was reversed one year after launching intersectoral activities for dengue control in the intervention area. Despite spraying actions in all areas, the differences in the Breteau index between intervention and control areas remain significant until December 2002. Although no differences were observed for the next two years, they became significant again in December 2004, corresponding with implementing the complementary community-based vector control strategy in the intervention area. |
| 12 | Cuba | Zayas, 2012** | To reduce the environmental factors of the community influencing *Ae. aegypti* infestation. | Pre-post (before-after) study | Health education campaign. | Removal of mosquito breeding sites and the positivity rate of the *Ae. aegypti* foci | The implemented strategy eliminated potential mosquito breeding sites. With this intervention, it was possible to reduce the infestation index of *Ae. aegypti* in high-risk blocks, from 0.5-0.02. Of 72 risks detected, 65 (90.3%) were resolved, and 7 (9.7%) remained pending. |
| 13 | Cuba | Montada Dorta, 2013** | To determine the effectiveness of pyrethroid insecticide beta-cypermethrin, formulated as sipertrin 5 SC, for the control of *Ae. aegypti.* | Pre-post (before-after) study | Three interventions with sipertrin 5 SC were performed: (i) residual treatment of resting sites-perifocal, (ii) impregnation of curtains, and (iii) combination of treatment 1 and 2. | Mosquito mortality | The greatest residual effect was obtained with the combined treatment, with an effectiveness of up to five months. In HHs undergoing resting site and perifocal treatment, residuality was four months. The product’s effectiveness was highest on plastic and concrete surfaces, with a 95 to 100% mortality rate for three months. On metal surfaces, mortality was 85%. |
| 14 | Cuba | Toledo, 2015 | To evaluate the incremental effectiveness and cost-effectiveness of insecticide-treated curtain deployment in eastern Cuba, a setting with comprehensive routine *Ae.* control and already low *Ae.* infestation levels. | CRT | Usage of the long-lasting insecticide-treated curtain (PermaNet). | House index, Breteau index, and cost analysis | Over the 18-month observation period after insecticide-treated curtain distribution, the adjusted house index rate ratio, intervention versus control clusters, was 1.15 (95% CI 0.57 to 2.34). |
| 15 | Cuba | Hernández, 2019** | To contrast the communication for arbovirus infection prevention implemented in Cuba with the population’s knowledge, perceptions, and practices concerning these diseases and their primary vector, the *Ae. aegypti* mosquito. | Mixed method study | Mass communication (health education) campaign. | KAP | The mass communication campaign had influenced the KAP of the community. However, information gaps continue to exist, and actions are required beyond just the provision of information. Actors involved in the communication campaign, e.g., doctors, nurses, operators, promotion community leaders, were insufficient. |
| 16 | Cuba | Toledo, 2017 | To evaluate the entomological and epidemiological effectiveness of periodical intra- and peri-domiciliary residual insecticide (deltamethrin) treatment and long-lasting insecticide-treated curtains. | CRT | Intervention group 1: residual insecticide (25% deltamethrin granular formulation to be dissolved in water, 20 g in 8 L) was sprayed every four months. Intervention group 2: usage of long-lasting insecticide-treated curtains. | Incidence of dengue and immature *Ae.* infestation | Despite a significant reduction in *Ae.* indices (Rate Ratio (RR) 0.54 (95%CI 0.32–0.89) in the first month after periodical intra- and peri-domiciliary residual insecticide (deltamethrin) treatment, the effect faded out over time, and the incidence of dengue was not reduced. Overall, there was no protective effect of both interventions in this setting. |
| 17 | Haiti | Lenhart, 2008 | To investigate the efficacy of insecticide-treated bednets in reducing *Ae. aegypti* populations and dengue transmission. | CRT | Usage of long-lasting insecticide-treated bednets. | Breteau index, house index, container, pupae per person index, and anti-dengue IgM seropositivity rates | At one-month post-intervention, all entomological indices declined, with house index and Breteau index in the intervention arm reduced by 6.7 (95% CI -10.6, -2.7; P < 0.01) and 8.4 (95% CI -14.1, -2.6; P < 0.01) respectively. By five months, all indices remained low, and house index, container index and Breteau index were also significantly lower than baseline in the control arm. An IgM serosurvey showed a 15.3% decrease (95% CI 5.0-25.5%, P < 0.01) in the number of IgM-positive individuals from baseline to the final survey. |
| 18 | Puerto Rico | Winch, 2002 | To document how different organisations have implemented the community-based dengue prevention program.  To measure current levels of KAP among program participants.  To assess the extent to which knowledge about dengue is reflected in the adequate control of mosquito larval habitats. | Mixed method study | Community-based prevention programs: (i) two school-based educational programs, (ii) posters and televised public service announcements, and (iii) children’s museum exhibit on *Ae. Aegypti.* | KAP, house index, Breteau index, and container index | Exposure to the programs was associated with increased knowledge about dengue, increased proportion of tires protected from rain, and decreased proportion of water storage containers positive for mosquito larvae. Exposure to the elementary school program was associated with slightly lower indices of residential mosquito infestation. |
| 19 | Puerto Rico | Barrera, 2008 | To test the efficacy of treating all containers believed to produce most *Ae. aegypti* pupae in a short period to control the biting adult population. | NRCT | All containers likely to be aquatic habitats were turned over, and containers too large to turn were treated with one ppm methoprene. | The number of resting adult mosquitoes and pupae density | The pre-intervention and post-intervention densities of resting *Ae. aegypti* adult mosquito was significantly larger in the intervention area, although pupae density in surface containers was low and similar in both towns at four weeks post-intervention. At three weeks post-intervention, the density of resting adults decreased by only 18% of pre-intervention levels but returned to pre-intervention levels five weeks after treatment. Geographical Information Systems identified significant clustering of adult mosquitoes, which led to the discovery of underground aquatic habitats (e.g., septic tanks) that produced large numbers of *Ae. aegypti* and *Culex quinquefasciatus* (Say) in the intervention area. |
| 20 | Puerto Rico | Barrera, 2014 | To test the effectiveness of the novel CDC autocidal gravid ovitrap (AGO trap) to control natural populations of *Ae. aegypti* under field conditions in two isolated urban areas (reference vs intervention areas) in southern Puerto Rico for one year. | NRCT | Ovitrap (CDC-AGO trap). | The density of *Ae. aegypti* mosquito | There were significant reductions in the captures of female *Ae. aegypti* (53–70%) in the intervention area. |
| 21 | Puerto Rico | Barrera, 2014 | To determine if the sustained reduction in the *Ae. aegypti* female populations observed during the first year of the investigation in an urban area was maintained over time. To examine if adding intervention traps to a site initially used as a non-intervention reference area would succeed in lowering the population abundance of female *Ae. aegypti* to levels that have been observed in a separate intervention site. | NRCT | Usage of autocidal gravid ovitraps and source reduction management. | The density of *Ae. aegypti* female mosquitoes | Mosquito density in the former reference area (the new intervention area) decreased 79%, and mosquito density in the new reference areas was 88% greater than in the intervention areas. |
| 22 | Puerto Rico | Lorenzi, 2016 | To assess the incidence of chikungunya virus infection in communities with ongoing *Ae. aegypti* mosquito trap intervention studies. | NRCT | Usage of AGO traps. | Incidence of chikungunya virus infection and mosquito density | Lower incidence of chikungunya virus infection in the intervention compared with the control group occurred in the context of tenfold lower mosquito densities in the intervention areas with AGO traps. |
| 23 | Puerto Rico | Barrera, 2017 | To compare chikungunya virus incidence in *Ae. aegypti* in areas with and without AGO control traps to test the hypothesis that the presence of control traps limited local outbreaks of chikungunya virus infection. | NRCT | Usage of AGO traps. | Female *Ae. a*egypti density, viral RNA of dengue virus and chikungunya virus in mosquitoes | The density of *Ae. aegypti* females were 10.5 times lower in the two areas with AGO control traps. Ten times more chikungunya virus-positive pools were identified in the control areas than in intervention areas. We found a significant linear relationship between the number of positive pools and both densities of *Ae. aegypti* and vector index. Temporal and spatial patterns of positive chikungunya virus pools suggested limited virus circulation in intervention areas. |
| 24 | Puerto Rico | Barrera, 2018 | To test if integrated vector control using a combination of source reduction, larviciding, and Autocidal Gravid Ovitraps (AGO traps) applied to at least 80% of houses in an area with a 150 m radius significantly reduced the density of *Ae. aegypti* around a pre-selected house in its centre. | NRCT | The intervention consisted of eliminating, cleaning, or modifying containers, applying larvicide (Altosid Pro-G), and placing three AGO traps in the backyards of houses. | *Ae. aegypti* density | Overall, pre-treatment mosquito densities in the inner (0–50 m; 15,6 mosquitoes/trap/week), intermediate (50–100 m; 18,1), and outer rings (100–150 m; 15,6) were reduced after treatment to 2.8, 4.1, and 4.3 in the inner, middle, and outer rings, respectively. Density at the target house in the intervention area changed from 27.7 mosquitoes/trap/week before to 2.1 after intervention (92.4% reduction). After treating the original control area (cross-over), density changed from 22.4 to 3.5 (84.3% reduction). |
| 25 | Puerto Rico | Juarbe-Rey, 2018 | To examine the process and outcomes of a risk communication initiative to increase Zika virus awareness and health-enhancing behaviours among low-income housing residents. | Pre-post (before-after) study | Health education campaign focussing on community mobilisation. | KAP | Findings from baseline and follow-up data demonstrated significant positive changes in participants’ recognition of personal and community responsibility for preventing Zika virus infection, increased knowledge of prevention strategies, and enhanced engagement in preventive behaviours for mosquito control. |
| 26 | Puerto Rico | Barrera, 2019 | To investigate the control *Ae. aegypti* and Zika virus transmission in Caguas City, Puerto Rico during the 2016 epidemic using integrated vector management.  To determine if female adult *Ae. aegypti* density in the city could be brought down to a steady 2–3 per trap per week at this geographic scale. | CRT | Integrated vector management: community awareness and education, source reduction, larviciding, and mass-trapping with autocidal gravid ovitraps. | Adult female Ae. aegypti mosquitoes, vector index and maximum likelihood minimum infection rates | Out of 12.081 mosquito pools, there were one dengue virus, seven chikungunya virus, and 49 Zika virus-positive pools from October 2016 to March 2017. Afterwards, we found only one positive pool of dengue virus in July 2017. Mosquito density significantly changed (P< 0.001) from 8.0 ± 0.1 females per trap per week before the intervention to 2.1 ± 0.04 after the percentage of buildings treated with traps was 60% and to 1.4 ± 0.04 when coverage was above 80%. |
| 27 | Puerto Rico | Sharp, 2019 | To estimate the prevalence of chikungunya virus infection in communities with and without AGO traps and evaluate their effect on reducing chikungunya virus transmission. | NRCT | Usage of autocidal gravid ovitraps traps. | Chikungunya virus seropositivity | An estimated 26.1% (with autocidal gravid ovitraps) and 43.8% (without traps) of residents had been infected with the chikungunya virus (adjusted prevalence ratios = 0.50, 95% CI: 0.37–0.91). The monthly number of chikungunya virus-infected mosquitos and symptomatic residents were diminished in communities with traps compared to those without traps. |
| 28 | Puerto Rico | Harris, 2021 | To evaluate if control of *Ae. aegypti* could be achieved by repeated (weekly and biweekly) micro-droplet application of Bti AM65-52 pushed high into the air using vehicle-mounted air-blast spraying equipment, allowing the larvicide to be distributed by the wind across neighbourhoods and into a range of larval sources. | NRCT | Bti was applied at a rate of 500 g/ha using vehicle-mounted aqueous wide-area larvicide spray applications. | The population of adult female *Ae. Aegypti*, deposition of Bti droplets in open spaces, and building coverage | Bti spray was successfully deposited into jars in an array of open and covered locations, as confirmed by larval bioassays. After the fourth weekly spraying, differences in autocidal gravid ovitrap densities were observed between the intervention and control group resulting in 62% (P = 0.0001) and 28% (P < 0.0001) reductions in adult female *Ae. aegypti* numbers. |
| 29 | Trinidad | Chadee, 2005 | To examine the impact of routine vector control operations during the 1998 outbreak of dengue fever and dengue haemorrhagic fever in Trinidad, West Indies. | Record-surveillance | House inspection, treat water-holding containers with temephos. All houses of suspected /dengue cases were treated with temephos (all water-holding containers), and walls of houses intra-domiciliary treated with fenthion insecticide. In addition, 100 houses were spaced-sprayed using malathion insecticide applied by thermal foggers. | Breteau index, and incidence of dengue fever and dengue haemorrhagic fever | The Breteau index was >10 for 75% of the dengue haemorrhagic fever cases indicating a high vector activity. The Breteau indices in all these counties remained in excess of five throughout the year, thus maintaining the risk of dengue transmission. These results suggest that vector control operations failed to achieve the desired target of reducing mosquito densities to below the disease transmission threshold or possibly a Breteau index of five. |
| 30 | Trinidad | Chadee, 2009 | To test the efficacy of a new approach to the control of *Ae. aegypti,* which aims to reduce populations during periods of the anticipated rapid increase in population density, as predicted by dengue early warning systems. | NRCT | Houses were inspected and focally treated with insecticides temephos. | Breteau index, container index, house index, and pupae/person index | Following focal treatment during April (2-3 weeks before the onset of the rainy season), the *Ae. aegypti* population declined significantly (P>0.01) from a Breteau index of 19.0 to a minimum of 6.0 and a pupae/person index of 1.23 to a minimum of 0.35 in May, while in the untreated town of St. Joseph, the Breteau index steadily increased from 23 to 38. The pupae/person index rose from 0.96 to 2.00 in August. |
| 31 | 15 islands of the English-speaking Caribbean | Nathan, 2004 | To describe an initiative on community mobilisation and organisational change implemented through the Caribbean Cooperation in Health/ Government of Italy integrated vector control project. | Pre-post (before-after) study | Health education campaign focusing on community mobilisation. | KAP and entomological indices | The most significant change resulting from this project was how national vector control teams approached and worked together with communities and other stakeholders to resolve issues of mutual concern. There was a shift away from a ‘top-down’ approach to one of dialogue, negotiation and partnership to resolve environmental sanitation and vector control problems. Although KAPs were favourably altered and entomological indices were reduced in most project communities, the magnitude of these changes was modest and unlikely to be significant. |

**Note:**

*Portuguese

** Spanish

CRT: Cluster Randomised Trial

NRCT: Non-randomised control trail

Ha: Hectare

HHs: Households

KAP: Knowledge, Attitude and Practices

Bti: *Bacillus thuringiensis var israeliensis*

PPM: One part per million, equivalent to one gram of active ingredient in 1 million millilitres of water.
