# Supplementary material for: Effectiveness of Aedes-borne infectious disease control in Latin America and the Caribbean region: A scoping review
Source: PLoS One. 2022 Nov 2;17(11):e0277038. doi: 10.1371/journal.pone.0277038 (PMC9629598; doi:10.1371/journal.pone.0277038)
Supplement: S5 Table — (DOCX) [file pone.0277038.s005.docx]

S5 Table Characteristics of studies identified in the Latin America and Caribbean region about *Ae. aegypti* and *Ae. albopictus* prevention and control interventions

|  | **Country** | **Author, year** | **Objective (s)** | **Study design** | **Type of intervention (s)** | **Outcome (s)** | **Main results** |
| --- | --- | --- | --- | --- | --- | --- | --- |
| 1 | Dominican Republic, El Salvador, Guatemala, Haiti, Honduras, Jamaica, Nicaragua, Ecuador, Paraguay and Peru, Eastern and Southern Caribbean (Multi-country intervention study) | USAID report, n.d. | To improve institutional capacity building and infrastructure strengthening in countries where Zika Airs Project (ZAP) was implemented between 2016-2019 | NRCT | The intervention was implemented across the region of Latin America and the Caribbean and consisted of three pillars:  (i) entomological monitoring and surveillance through larvae/pupae surveys and several mosquito collection methods, including Prokopack aspirators, Gravid *Ae.* Traps, and Biogent sentinel traps for adult collection, ovitraps for egg collection, and manual collection for larvae.  (ii) vector control activities included larviciding with Bti and indoor residual spraying.  (iii) environmental management through household visits and cleaning up areas around homes to eliminate places where mosquitoes can breed, and educating householders on common breeding sites. | Removal of mosquito breeding sites, behaviour change of community and entomological indices such as container index | In general, the performed interventions led to a reduction of all forms of *Ae.* mosquitoes. ZAP provided extensive institutional capacity building and infrastructure strengthening to support robust routine entomological monitoring in collaboration with Ministries of Health and local stakeholders. ZAP initiated the development, delivery, and/or refurbishment of insectaries and laboratories and the Mosquito Control Research Unit. ZAP also built local capacity to conduct insecticide susceptibility tests to guide vector control decisions and equipped country governments to carry forward policies, protocols, and decision-making that rely on reliable, accurate, and real-time data, ensuring they are better able to prepare for future outbreaks. |
