# Supplementary material for: Effectiveness of Aedes-borne infectious disease control in Latin America and the Caribbean region: A scoping review
Source: PLoS One. 2022 Nov 2;17(11):e0277038. doi: 10.1371/journal.pone.0277038 (PMC9629598; doi:10.1371/journal.pone.0277038)
Supplement: Attachment — Submitted filename: PONE-D-22-16967+GP.pdf [file pone.0277038.s007.pdf]

PLOS ONE

Effectiveness of Aedes-borne infectious disease control in Latin America and the Caribbean region: a scoping review

--Manuscript Draft--

Manuscript Number: PONE-D-22-16967

Article Type: Research Article

Full Title: Effectiveness of Aedes-borne infectious disease control in Latin America and the Caribbean region: a scoping review

Short Title: Aedes-borne infectious disease control in Latin America and the Caribbean region

Corresponding Author: Vaitiare Mulderij-Jansen, MSc
Universitair Medisch Centrum Groningen Afdeling Medische Microbiologie en Infectiepreventie
Groningen, NETHERLANDS

Keywords: Zika Virus Infection; Vector Borne Diseases; Mosquito Control; Public Health, Environment and Public Health; Dengue

Abstract:

Abstract

Background
Aedes aegypti and Aedes albopictus are primary vectors of emerging or re-emerging arboviruses that threaten public health worldwide. Many efforts have been made to develop interventions to control these Aedes species populations. Still, countries in the Latin America and the Caribbean (LAC) region struggle to create/design/develop sustainable and effective control strategies. This scoping review synthesises evidence concerning the effectiveness of Aedes aegypti and Aedes albopictus prevention and control interventions performed in LAC (2000-2021). The findings can be used to evaluate, compare and develop more effective control strategies.

Methodology
The review is based on the methodology by Joanna Briggs Institute for conducting a scoping review. The MEDLINE (via PubMed and Web of Science), Cochrane Library, Scopus, EMBASE and ScienceDirect databases were used to search for articles. Grey literature was searched from governmental and non-governmental organisation websites. Four reviewers independently screened all titles and abstracts and full-text of the articles using the Rayyan web application, based on pre-defined eligibility criteria.
Results
A total of 122 publications were included in the review. Most studies focused on dengue virus infection and data on Aedes aegypti . Entomological data were mainly used to determine the intervention's effectiveness. An integrated control intervention was the most commonly employed strategy in both regions. Biological control measures, environmental management, and health education campaigns on community participation achieved more sustainable results than an intervention where only a chemical control measure was used. Challenges to implementing interventions were insufficient financial support, resources, workforce, intersectoral collaboration and legislation.

Conclusions
An integrated vector (Aedes) management focused on community participation seems to be the most effective approach to mitigate Aedes -borne infectious diseases. Maintaining the approach's effect remains challenging as it requires multisectoral and multi-disciplinary team engagement and active community participation. Future research needs to address the barriers to program implementation and maintenance as data on this topic is lacking.

Order of Authors:

Vaitiare Mulderij-Jansen, MSc

Prachi Pundir, MSc

Maria E Grillet, MSc, PhD, Prof.

Powered by Editorial Manager® and ProduXion Manager® from Aries Systems Corporation

	Theophilus Lakiang, MSc
	Izzy Gerstenbluth, MD, MSc
	Ashley Duits, MSc, PhD, Prof.
	Adriana Tami, MD, MSc, PhD
	Ajay Bailey, MSc, PhD, Prof.
**Additional Information:**	
**Question**	**Response**
**Financial Disclosure**<br><br>Enter a financial disclosure statement that describes the sources of funding for the work included in this submission. Review the submission guidelines for detailed requirements. View published research articles from *PLOS ONE* for specific examples.<br><br>This statement is required for submission and **will appear in the published article** if the submission is accepted. Please make sure it is accurate.<br><br>**Unfunded studies**<br>Enter: *The author(s) received no specific funding for this work.*<br><br>**Funded studies**<br>Enter a statement with the following details:<br>• Initials of the authors who received each award<br>• Grant numbers awarded to each author<br>• The full name of each funder<br>• URL of each funder website<br>• Did the sponsors or funders play any role in the study design, data collection and analysis, decision to publish, or preparation of the manuscript?<br>• **NO** - Include this sentence at the end of your statement: *The funders had no role in study design, data collection and analysis, decision to publish, or preparation of the manuscript.*<br>• **YES** - Specify the role(s) played.<br><br>* typeset	Funding was provided by the Netherlands Organisation for Scientific Research (NWO) for the project entitled "Public health impact of chronic chikungunya illness and performance/utilisation of the health care system in the face of arboviral (dengue, chikungunya, Zika) epidemics in Curacao" Acronym: ARBOCARIB (NWO grant ALWCA.2016.021). The funders had no role in study design, data collection and analysis, decision to publish or preparation of the article.
**Competing Interests**<br><br>Use the instructions below to enter a competing interest statement for this submission. On behalf of all authors,	The authors have declared that no competing interests exist.

disclose any competing interests that could be perceived to bias this work—acknowledging all financial support and any other relevant financial or non-financial competing interests.<br><br>This statement is required for submission and **will appear in the published article** if the submission is accepted. Please make sure it is accurate and that any funding sources listed in your Funding Information later in the submission form are also declared in your Financial Disclosure statement.<br><br>View published research articles from *PLOS ONE* for specific examples.<br><br>**NO authors have competing interests**<br><br>Enter: *The authors have declared that no competing interests exist.*<br><br>**Authors with competing interests**<br><br>Enter competing interest details beginning with this statement:<br><br>*I have read the journal's policy and the authors of this manuscript have the following competing interests: [insert competing interests here]*<br><br>* typeset	
**Ethics Statement**<br><br>Enter an ethics statement for this submission. This statement is required if the study involved:<br><br>• Human participants<br>• Human specimens or tissue<br>• Vertebrate animals or cephalopods<br>• Vertebrate embryos or tissues<br>• Field research<br><br>Write "N/A" if the submission does not require an ethics statement.	N/A

Powered by Editorial Manager® and ProduXion Manager® from Aries Systems Corporation

General guidance is provided below. Consult the submission guidelines for detailed instructions. **Make sure that all information entered here is included in the Methods section of the manuscript.**<br><br>**Format for specific study types**<br><br>**Human Subject Research (involving human participants and/or tissue)**<br>• Give the name of the institutional review board or ethics committee that approved the study<br>• Include the approval number and/or a statement indicating approval of this research<br>• Indicate the form of consent obtained (written/oral) or the reason that consent was not obtained (e.g. the data were analyzed anonymously)<br><br>**Animal Research (involving vertebrate animals, embryos or tissues)**<br>• Provide the name of the Institutional Animal Care and Use Committee (IACUC) or other relevant ethics board that reviewed the study protocol, and indicate whether they approved this research or granted a formal waiver of ethical approval<br>• Include an approval number if one was obtained<br>• If the study involved *non-human primates*, add *additional details* about animal welfare and steps taken to ameliorate suffering<br>• If anesthesia, euthanasia, or any kind of animal sacrifice is part of the study, include briefly which substances and/or methods were applied<br><br>**Field Research**<br><br>Include the following details if this study involves the collection of plant, animal, or other materials from a natural setting:<br>• Field permit number<br>• Name of the institution or relevant body that granted permission	
**Data Availability**<br><br>Authors are required to make all data	No - some restrictions will apply

Powered by Editorial Manager® and ProduXion Manager® from Aries Systems Corporation

underlying the findings described fully available, without restriction, and from the time of publication. PLOS allows rare exceptions to address legal and ethical concerns. See the PLOS Data Policy and FAQ for detailed information.<br><br>A Data Availability Statement describing where the data can be found is required at submission. Your answers to this question constitute the Data Availability Statement and **will be published in the article**, if accepted.<br><br>**Important:** Stating 'data available on request from the author' is not sufficient. If your data are only available upon request, select 'No' for the first question and explain your exceptional situation in the text box.<br><br>Do the authors confirm that all data underlying the findings described in their manuscript are fully available without restriction?	
**Describe where the data may be found in full sentences. If you are copying our sample text, replace any instances of XXX with the appropriate details.**<br><br>• If the data are **held or will be held in a public repository**, include URLs, accession numbers or DOIs. If this information will only be available after acceptance, indicate this by ticking the box below. For example: *All XXX files are available from the XXX database (accession number(s) XXX, XXX.).*<br>• If the data are all contained **within the manuscript and/or Supporting Information files**, enter the following: *All relevant data are within the manuscript and its Supporting Information files.*<br>• If neither of these applies but you are able to provide **details of access elsewhere**, with or without limitations, please do so. For example:<br><br>*Data cannot be shared publicly because of [XXX]. Data are available from the*	The submitted manuscript is a scoping review. The findings and conclusions are based on published data.

Powered by Editorial Manager® and ProduXion Manager® from Aries Systems Corporation

XXX Institutional Data Access / Ethics Committee (contact via XXX) for researchers who meet the criteria for access to confidential data.<br><br>*The data underlying the results presented in the study are available from (include the name of the third party and contact information or URL).*<br>• This text is appropriate if the data are owned by a third party and authors do not have permission to share the data.<br><br>* typeset	
Additional data availability information:	

Manuscript
Click here to access/download;Manuscript;Scoping review 4-07-2022.docx

Effectiveness of *Aedes*-borne infectious disease control in Latin America and the Caribbean region: a scoping review

Vaitiare Mulderij-Jansen[1,2,3*], Prachi Pundir[4], Maria E. Grillet[5], Theophilus Lakiang[6], Izzy Gerstenbluth[3,7], Ashley Duits[8,9,10], Adriana Tami[1], Ajay Bailey[2]

[1] University of Groningen, University Medical Center Groningen, Department of Medical Microbiology and Infection Prevention, Groningen, The Netherlands

[2] International Development Studies, Department of Human Geography and Spatial Planning, Faculty of Geosciences, Utrecht University, Utrecht, Netherlands

[3] Curaçao Biomedical & Health Research Institute, Department of Epidemiology, Willemstad, Curaçao

[4] George Institute for Global Health, New Delhi, India

[5] Instituto de Zoología y Ecología Tropical, Facultad de Ciencias, Universidad Central de Venezuela, Venezuela

[6] Bridge Medicals Consulting Private Limited, Delhi, India

[7] Epidemiology and Research Unit, Ministry of Health Environment and Nature of Curaçao, Willemstad, Curaçao

[8] Red Cross Blood Bank Foundation, Willemstad, Curaçao

[9] Curaçao Biomedical & Health Research Institute, Department of Immunology, Willemstad, Curaçao

[10] Institute for Medical Education, University Medical Center Groningen, Groningen, The Netherlands

*Corresponding author

E-mail address: v.i.c.jansen@umcg.nl

Abstract

Background

Aedes aegypti and *Aedes albopictus* are primary vectors of emerging or re-emerging arboviruses that threaten public health worldwide. Many efforts have been made to develop interventions to control these *Aedes* species populations. Still, countries in the Latin America and the Caribbean (LAC) region struggle to create/design/develop sustainable and effective control strategies. This scoping review synthesises evidence concerning the effectiveness of *Aedes aegypti* and *Aedes albopictus* prevention and control interventions performed in LAC (2000-2021). The findings can be used to evaluate, compare and develop more effective control strategies.

Methodology

The review is based on the methodology by Joanna Briggs Institute for conducting a scoping review. The MEDLINE (via PubMed and Web of Science), Cochrane Library, Scopus, EMBASE and ScienceDirect databases were used to search for articles. Grey literature was searched from governmental and non-governmental organisation websites. Four reviewers independently screened all titles and abstracts and full-text of the articles using the Rayyan web application, based on pre-defined eligibility criteria.

Results

A total of 122 publications were included in the review. Most studies focused on dengue virus infection and data on *Aedes aegypti*. Entomological data were mainly used to determine the intervention's effectiveness. An integrated control intervention was the most commonly employed strategy in both regions. Biological control measures, environmental management, and health education campaigns on community participation achieved more sustainable results than an intervention where only a chemical control measure was used. Challenges to implementing interventions were insufficient financial support, resources, workforce, intersectoral collaboration and legislation.

Conclusions

An integrated vector (*Aedes)* management focused on community participation seems to be the most effective approach to mitigate *Aedes*-borne infectious diseases. Maintaining the approach's effect remains challenging as it requires multisectoral and multi-disciplinary team engagement and active community participation. Future research needs to address the barriers to program implementation and maintenance as data on this topic is lacking.

Introduction

Worldwide, there are approximately 3,500 registered mosquito species [1]. Only a small portion of these species carry and transmit pathogens to humans [2, 3]. Arboviral (arthropod-borne viral) diseases account for more than 17% of infectious diseases worldwide, affecting millions of people [4]. The global incidence of dengue virus infection has increased 30-fold in the last decades [5]. Dengue virus infection is an important public health issue with increasing morbidity and mortality in Latin America and the Caribbean region (LAC) [6]. Besides the dengue virus, chikungunya and Zika viruses are also of concern. They have rapidly spread throughout the LAC region in the last eight years, resulting in epidemics and high levels of morbidity, creating an added burden on the region's health systems [7].

Mosquitoes of the *Aedes* genus are considered the most important vectors of the mentioned arthropod-borne viruses [8]. In the LAC, *Aedes aegypti* is the primary mosquito vector of dengue, chikungunya, and Zika viruses [9]. In addition, *Aedes albopictus* (the Asian tiger mosquito) has also been shown to be a competent vector of the above-reported viruses [10, 11]. *Aedes albopictus* is of medical importance due to its aggressive daytime human-biting behaviour, ability to adapt to colder climates and live in artificial and natural containers close to humans, resulting in disease transmission in new geographical areas. Since *Aedes albopictus* is also present in the LAC, countries in this region must consider this mosquito species' possible implications for transmitting viruses [12, 13].

In the last decades, efforts have been made to develop vaccines, drugs, and mosquito control interventions to prevent and control diseases transmitted by *Aedes aegypti* and *Aedes albopictus* (*Aedes*-borne infectious diseases [ABIDs]). A licensed dengue vaccine is available; however, it is not widely used due to safety concerns [14]. For chikungunya and Zika virus infections, *Aedes* control is currently the only method available to prevent and control

transmission. *Aedes* control aims to limit the transmission of pathogens by reducing or eliminating human contact with the mosquito.

The World Health Organization (WHO) recommends using an Integrated Vector Management (IVM) program to manage mosquito populations [15]. The characteristic features of the IVM program include (i) selection of methods based on knowledge of local vector biology, disease transmission and morbidity; (ii) utilisation of a range of interventions (e.g., biological, chemical control measures, community mobilisation), often in combination and synergistically; (iii) collaboration within the health sector and with other public and private sectors; (iv) engagement with local communities and stakeholders; (v) a public health regulatory and legislative framework; (vi) rational use of insecticides; and (vii) good management practices. Ideally, the proper implementation of an IVM program could help countries deal with ABIDs. However, many countries, including countries in the LAC region, face challenges such as insufficient resources, collaboration within the health sector and with the public and private sector, workforce, training, and/or issues with immigration or cross-border transmission, which obstruct the development and implementation of the IVM program [16, 17]. On top of the mentioned challenges, other emerging infectious disease pandemics/ epidemics, such as the (COVID-19) SARS-CoV-2 pandemic, continues to impose substantial stress on health care systems, further jeopardising *Aedes* control interventions.

Previous outbreaks of ABIDs have shown the weakness in managing infectious disease spread in the LAC region [17, 18]. Based on the history of *Aedes aegypti* and *Aedes albopictus*, it is undeniable that other ABIDs (e.g., Mayaro, Yellow fever, Venezuelan Equine Encephalitis and West Nile virus infections) represent emerging threats favoured by climate change, globalisation, and travel/ trade [19-21]. The danger of the emerging or re-emerging of diseases transmitted by *Aedes aegypti* and *Aedes albopictus* requires us to learn from our experiences. Also, we need to work towards more sustainable integrated *Aedes* control strategies, taking the current challenges of countries in the LAC region into account.

There are recent comprehensive reviews about interventions to control *Aedes aegypti* [22]. However, to our knowledge, reviews covering more than one *Aedes* species and ABID are lacking in scientific literature. Therefore, our review attempts to synthesise the evidence (scholarly journal articles and grey literature) regarding the effectiveness of *Aedes aegypti* and *Aedes albopictus* prevention and control interventions (against dengue, chikungunya and Zika virus) performed in LAC in the last twenty-one years (2000-2021). Furthermore, the reported challenges, lessons learned and recommendations to deal with the practical challenges in applying *Aedes* control interventions will be documented in the review. Countries in the LAC region can use the evidence generated from this article to (i) evaluate their IVM program holistically, (ii) compare their *Aedes aegypti* and *Aedes albopictus prevention and* control strategies with neighbouring countries, and (iii) apply lessons learned to develop more sustainable *Aedes* control approach to prevent and manage ABIDs in the era of COVID-19 pandemic and beyond.

Materials and Methods

This scoping review is based on the framework proposed by Arksey and O'Malley, which has been further developed by Levac *et al.* and the methods by Joanna Briggs Institute (JBI) [23-25]. The JBI framework recommends organising the review process in at least five stages: (i) identifying the research question, (ii) identifying relevant studies, (iii) study selection, (iv) charting the data, and (v) collating, summarising and reporting the results. The scoping review also adheres to the Preferred Reporting Items for Systematic reviews and Meta-Analyses extension for Scoping Reviews (PRISMA-ScR) checklist (S1 Table) [26]. The protocol of this scoping review has been registered in Open Science Framework (OSF), link: https://osf.io/dkzht/wiki/home/.

The eligibility criteria for the review were based on the 'Population-Intervention-Comparison-Outcome' (PICO) framework and not the PCC (Population-Concept-Context) framework suggested by JBI. The PICO framework was chosen because it leads to comprehensive

search strategies and yields more precise results. The included studies followed the following requirements:

Population: The population included residents of the Caribbean islands and countries of the Latin American region of all age groups irrespective of gender, income, occupation, or other demographic characteristics. Each article was screened to check if the reported study population met our eligibility criteria. Articles that included hospitalised individuals with metabolic or fatal diseases/ terminal illnesses were excluded from the review.

Intervention: The primary concept of this scoping review is *Aedes aegypti* and *Aedes albopictus* prevention and control interventions, including (i) insecticide-treated materials (e.g., curtains, nets or screens), (ii) usage of larvicides in breeding sites, (iii) usage of adulticides (e.g., outdoor fogging and indoor residual spraying), (iv) lethal oviposition trap-based mass interventions, (v) container management/reduction, (vi) health education, (vii) community engagement, (viii) media campaigns, (ix) biological control measures (e.g., usages of other living organisms, such as larvivorous fish and *Wolbachia*), (x) mosquito coils/ repellents, (xi) interventions focussed on behavioural change, advocacy (informed influence activities on policymakers from civil society), and (xii) integrated surveillance, epidemiological or entomological surveillance as part of a control program. All field experiments that were not population-based simulations or semi-field trials were excluded.

Comparison: The comparison of the interventions for ABIDs was no intervention or the usual/ older intervention. The comparison groups or control groups could be a combination of interventions, such as biological control measures and community engagement in one group and the same interventions in two other groups individually. Such comparisons assisted in testing the effectiveness of a specific intervention. The studies that used surveillance data to assess intervention effectiveness and the pre-post study designs with no comparison groups were also included in this review. Cross-sectional studies were included when the collected data were compared with previously collected data (e.g. surveillance data).

Context: This scoping review focused on countries located in the LAC region. The Latin American region consists of the following countries: Belize, Costa Rica, El Salvador, Guatemala, Honduras, Mexico, Nicaragua, and Panama in North and Central America. Argentina, Bolivia, Brazil, Chile, Colombia, Ecuador, French Guiana, Guyana, Paraguay, Peru, Suriname, Uruguay, and Venezuela are the countries in South America [27]. The Caribbean region consists of the following islands: Anegada, Anguilla, Antigua, Aruba, Bahamas, Barbados, Barbuda, Bonaire, Cayman Islands, Cuba, Curaçao, Dominica, Dominican Republic, Grenada, Grenadines, Guadeloupe, Haiti, Hispaniola, Jamaica, Jost Van Dyke, Martinique, Montserrat, Nevis, Puerto Rico, Saba, Saint Croix, Saint Martin, Saint Kitts, Sint Eustatius, Saint Barthélemy, Saint John, Saint Lucia, Saint Martin, Saint Thomas, Saint Vincent, Tortola, Trinidad and Tobago, Turks and Caicos Islands, Virgin Gorda, and Water Island [27].

Outcome: The reviewers included publications that report the effect of the intervention (s) on (i) egg, larva, pupa or adult mosquito density, including entomological indexes such as; the Breteau index [(number of containers infested / total houses inspected) x 100], the container index [(number of containers infested / total containers inspected) x 100], the house index [(number of houses infested / total households) x 100], pupae per person index [number of pupae per household population] and pupae per Hectare index [number of pupae per household area], (ii) ABID incidence, (iii) knowledge, attitude, and practice (KAP), and (iv) environmental adaptations such as the reduction of mosquito breeding sites.

Time-frame, language and other criteria: This scoping review does not focus on intervention cost, but studies containing information on both cost and effect of intervention were considered. The included articles were (i) published between January 1st 2000, and May 28th 2021, and (ii) were written in English, Spanish and Portuguese. An article was excluded if it (i) contained only entomological surveillance, epidemiological or prevalence data without a link to *Aedes aegypti* and *Aedes albopictus* prevention and control strategies; (ii) outcome of

interest not reported, (iii) not available in full-text; and (iv) written in French. Publications from French-speaking islands/countries were included if the publication was written in English, Spanish or Portuguese. The reviewers did not exclude an article based on the year of data collection; all publications starting from the year 2000 were included.

Electronic databases and other sources: The MEDLINE (via PubMed and Web of Science), Cochrane Library, Scopus, EMBASE and ScienceDirect databases were searched for articles. Additionally, Google Scholar and the Google search engine searched for the first 100 relevant results. Grey literature regarding *Aedes aegypti* and *Aedes albopictus* prevention and control interventions were searched on the governmental and non-governmental organisation websites such as the WHO library database, UNICEF, and Latin American and Caribbean Health Sciences Literature.

Study design: This scoping review shows evidence from primary studies or journal articles or organisational reports, or dissertations (e.g., cross-sectional studies- analytical, case-control studies, cohort studies, randomised and non-randomised controlled trials, impact evaluations, qualitative and mixed-methods studies). We decided not to include systematic or scoping or narrative reviews and meta-analyses because we were interested in the singular study findings. All such ’reviews’ reference lists were searched for additional primary studies on the research topic during the full-text screening stage. Conference proceedings, letters to the editor, editorials, and commentaries were excluded.

Search: Two team members (PP and VJ) experienced in searching databases designed the search strategy (S2 Table). Keywords were identified based on three domains; (i) mosquito-borne infectious diseases, (ii) location, and (iii) *Aedes aegypti* and *Aedes albopictus* prevention and control interventions using Medical Subject Headings [MeSH], existing literature reviews, and subject experts’ opinions. The keywords were combined with appropriate boolean operators to search for articles in electronic databases, and similar keywords were used for grey literature. Proximity operators, truncation and wildcards were

used for keywords to increase the sensitivity of the search. The search was initially conducted on PubMed and then tailored to other databases. PRESS checklist was used to review the search strategy's quality [28].

Selection of studies: The compiled search results were de-duplicated using EndNote 20 and exported to rayyan.ai to screen blinded [29]. The reviewers stored the articles and other data files using cloud storage technology. The selection of studies was performed by four reviewers independently (VJ, MG, PP, TL), and disagreements were resolved in discussion with the team. The articles were screened twice (i) in the title and abstract screening stage and (ii) in the full-text screening stage. The selection process is presented in the PRISMA flow chart [30]. Three reviewers (VJ, PP, TL) piloted data extraction on a sample of the included studies (5% of the complete list of retrieved studies) to ensure that the data extraction technique was consistently applied. The data extraction was conducted by three reviewers (VJ, PP, TL) using a charting table. Data were extracted for the following variables: study identifiers, locations, study design, methods, demography information, intervention, comparison, outcome, challenges and recommendations.

A second reviewer (VJ) cross-checked each entry. The reviewers discussed doubts until consensus was reached or consulted a fourth reviewer to resolve the disagreements.

Data analysis: The data were analysed using Microsoft Excel using basic formulae and data for the figure. Frequencies and percentages were calculated for the variables of interest for summarising the data, and a 'characteristics of included studies' table was prepared from the data charting sheet. Advanced analysis was not performed, and the quality assessment of the included studies was not carried out as both are not processes suggested by the JBI guidelines [25].

Results

The results are presented here by the following key themes: (i) characteristics of the included publications, (ii) differences and similarities between regions, (iii) interventions, (iv) challenges and lessons learned, and (v) strategies employed or recommended to improve the effectiveness of *Aedes* prevention and control measures.

Characteristics of the included publications

A total of 11,222 (11,118 articles from databases and 104 articles from other sources, e.g., google or reference list search) articles were identified by the reviewers in the initial search. After eliminating duplicates, the remaining articles were screened by reading their title and abstract. One hundred twenty-eight articles from databases and 101 articles from other sources were qualified and further screened by reading the full-text. Eighteen of the 128 articles from the databases and 89 articles from other sources were excluded based on the exclusion criteria. A total of 122 articles were included in the scoping review. Fig. 1 shows a flow diagram of the selection process.

Fig 1. PRISMA 2020 flow diagram

Of these 122 articles, 90 were from the Latin America region [31-120], and 31 [121-151] were from the Caribbean region. One publication contained information from both regions [152]. The characteristics of the included studies are presented in S3-S5: Tables. Most studies from the Latin America region were from Brazil (n = 30) [34-63], Mexico (n = 21) [89-108, 119] and Colombia (n = 18) [64-81] (Table 1). Most studies from the Caribbean region were from Cuba (n = 16) [121-136], Puerto Rico (n = 11) [138-148], and Trinidad (n = 2) [149, 150] (Table 1). Basic characteristics of the included studies are presented in Table 1, and the number of publications for each year (2000-2021) is illustrated in Fig 2.

Table 1. Characteristics of the included studies

	Caribbean n (%) (n = 31)	Latin America n (%) (n = 90)
**Type of Publication**		
Journal article	30 (96.8)	88 (97.8)
Report	1 (3.2)	0
Thesis/ dissertation	0	2 (2.2)
**Language**		
English	26 (83.9)	66 (73.3)
Spanish	5 (16.1)	16 (17.8)
Portuguese	0	8 (8.9)
**Country (Top 3)**		
Brazil	0	30 (33.3)
Mexico	0	21 (23.3)
Colombia	0	18 (20.0)
Cuba	16 (51.6)	0
Puerto Rico	11 (35.5)	0
Trinidad	2 (6.4)	0
**Setting**		
Urban	10 (32.2)	41 (45.6)
Rural	0	4 (4.4)
Both	3 (9.7)	7 (7.8)
Not mentioned	18 (58.1)	38 (42.2)
**Study designs**		
Randomized controlled trial	0	5 (5.6)
Non-randomized controlled trial	15 (48.4)	29 (32.2)
Cluster randomized trial	7 (22.6)	24 (26.7)
Pre-post (before-after) study	6 (19.4)	17 (18.9)
Mixed method study	2 (6.4)	2 (2.2)
Record surveillance	1 (3.2)	9 (10.0)
Cross-sectional study	0	3 (3.3)
Other*	0	1 (1.1)
**Demographics mentioned**		
Yes	6 (19.4)	33 (36.7)
No	25 (80.6)	57 (63.3)
**Type of disease**		
Dengue	22 (71.0)	75 (83.3)
Zika	1 (3.2)	0
Chikungunya	1 (3.2)	0
Other (combination)	7 (22.6)	15 (16.7)
**Type of mosquito**		
Aedes aegypti	29 (93.5)	74 (82.2)
Aedes aegypti and *Aedes albopictus*	2 (6.5)	13 (14.4)
Aedes	0	2 (2.2)
Not mentioned	0	1 (1.1)
**Type of intervention (s)**		

A. Application of larvicide (biological and chemical)[**]	2 (6.5)	4 (4.4)
B. Adulticiding (spraying of insecticide indoor/outdoor)	0	10 (11.1)
C. Biological control (usage of larvivorous fish and *Wolbachia*)	0	3 (3.3)
D. Environmental management (removal or covering of breeding sites, and usage of insecticide-treated curtains/screens)	2 (6.5)	14 (15.6)
E. Traps (including lethal traps for immature forms and adult mosquitoes)	4 (12.9)	7 (7.8)
F. Genetically modified mosquitoes	0	1 (1.1)
G.Health education and community mobilisation	5 (16.1)	17 (18.9)
H. Integrated approach[***]	16 (51.6)	26 (28.9)
A&B	1 (3.2)	0
A&D	0	3 (3.3)
B&D	1 (3.2)	0
B&G	0	1 (1.1)
D&G	0	4 (4.4)
**Type of outcomes**		
A. Entomological data (e.g., survey of mature/ immature mosquitoes)	9 (29.0)	40 (44.4)
B. Incidence of mosquito-borne infectious diseases	1 (3.2)	2 (2.2)
C. Knowledge-Attitude-Awareness-practices-perceptions	2 (6.5)	4 (4.4)
A&B	4 (12.9)	10 (11.1)
A&C	5 (16.1)	12 (13.3)
Other[****]	10 (32.3)	22 (24.4)

Note: The reviewers included one report documenting interventions performed in countries located in both Latin America and the Caribbean region. This report (USAID, 2019) is written in English, focusing mainly on Zika, but dengue was also mentioned. The following interventions were performed: biological and chemical control measures, environmental management, usage of traps and health education. The outcomes were: entomological data, removal of mosquito breeding sites, and improvement of knowledge, attitude and practices. The study designs were classified as non-randomized control trials.

Demographic information: study participants' age, gender, and income or employment status.

[*] Economic modelling assessment.

[**] Application of larvicide (biological and chemical): *Bacillus thuringiensis var. israelensis*, Diflubenzuron, Novaluron, Spinosad, Temephos and Pyriproxyfen.

[***] Integrated approach refers to a control strategy with more than three components (e.g., health education, environmental management and application of larvicide.

[****]Type of outcome: More than three types of outcome (e.g., self-reported dengue symptoms and anti-dengue IgM seropositivity rates combined with entomological parameters) or a type of outcome that has not been categorised, including removal of mosquito breeding sites, community participation, maintenance of activities through capacity building, Disability-adjusted life years (DALYs) and cost of interventions.

Fig 2. The number of publications per year (2000-2021)

Differences and similarities between the Caribbean and the Latin America region

Most of Latin America and the Caribbean region's publications have focused on the dengue virus infection, urban areas, and *Aedes aegypti* mosquitoes (Table 1). The *Aedes albopictus* mosquito has received more attention in Latin America than in the Caribbean. Chemical control measures were often applied in both areas compared to biological control measures. Mostly entomological data were used to determine the intervention's effectiveness, and it was more often used in Latin America than in the Caribbean region (Table 1). Health education campaigns were the most commonest employed (single) intervention to control ABIDs in both regions.

Interventions

Application of larvicide in mosquito breeding sites

A study conducted in Brazil assessed the effect of a conjugate of *lysinibacillus sphaericus (Lsp)* and *Bacillus thuringiensis var. israelensis (Bti)* on *Aedes* eggs and adult mosquito populations [59]. No significant reduction in the *Aedes aegypti* adult population was proven, but a significant reduction in egg density was observed in the second year compared to the

first year. In Puerto Rico, *Bti* was applied at a rate of 500 grams/ Hectare using vehicle-mounted aqueous wide-area larvicide spray applications [148]. This study found that *Bti* was successfully deposited into jars in both open and covered locations. After the intervention, differences in ovitrap densities were observed between the intervention and control group resulting in 62% (P = 0.0001) and 28% (P < 0.0001) reductions in adult female *Aedes aegypti* mosquitoes [148]. In Colombia, treating street catch basin with 2 grams of pyriproxyfen (approx. 0.05 mg/mL) was associated with a reduction in the incidence of dengue (rate ratio 0.19, 95% CI 0.12–0.30, P < 0.0001) [73]. Two studies conducted in Brazil used pyriproxyfen to control *Aedes* mosquitoes [51, 55]. One study found that the mentioned intervention led to a ten-fold decrease in adult mosquito emergence from sentinel breeding sites [51]. The second study also showed the beneficial effect of pyriproxyfen. After the intervention, *Aedes* juvenile catch decreased by 79%–92%, and juvenile mortality increased from 2%–7% to 80%–90%. Also, the mean adult *Aedes* emergence fell from 1.077 per month (range 653–1,635) at baseline to 50.4 per month during the intervention (range 2–117) [55]. In Peru, mosquito breeding sites were treated with pyriproxyfen, but no significant effects (Breteau index: −1.05 with a 95% CI: −12.64–10.53 and pupae per person index: 0.365 with a CI: −0.030–0.760) were observed [119].

A trial in Mexico assessed the effect of the application of spinosad (1 part per million [ppm], equivalent to one gram of active ingredient in one million millilitres of water), spinosad (5 ppm), 0.4 gram 1% temephos granules, and 50 µl Vectobac AS12 *(Bti)* on immature forms of *Aedes* mosquitoes located in car tires [91]. Both spinosad treatments (1 and 5 ppm) provided 6-8 weeks of effective control *of Aedes aegypti and Aedes albopictus* in the dry and the rainy season. *Bacillus thuringiensis var. israelensis* controlled *Aedes* larvae for one week. The duration of larvicidal activity of 1% temephos granules was intermediate between the spinosad treatments and *Bti.* A study in Trinidad that treated breeding sites with temephos (2-3 weeks before the onset of the rainy season) reported a significant decline of the *Aedes aegypti*

population ($P > 0.01$), from a Breteau index of 19.0 to 6.0 and a pupae per person index of 1.23 to 0.35 [150].

The evidence shows the beneficial effect of larvicide application in mosquito breeding sites before and during the rainy season. The application of *Bti*, pyriproxyfen, spinosad and temephos in mosquito breeding sites led to a decline in the immature and adult *Aedes* mosquito populations. The usage of pyriproxyfen in breeding sites was associated with a decline in the incidence of dengue virus infection.

Spraying of insecticide (indoor/outdoor)

Two studies that used insecticide spraying in Argentina [31, 33] and Peru [113, 114] were identified. After applying insecticide, these studies reported a reduction in *Aedes* mosquito larval indices and/ or adult density. In Brazil, the spraying of insecticide (malathion) using heavy equipment was associated with fewer cases of ABIDs compared to the control group (application of insecticide with portable equipment) [57]. Another study in Brazil found that ultra-low insecticide spraying avoided about 24% of symptomatic dengue cases in the study area throughout the 2015-2016 (December 1, 2015 - June 30, 2016) dengue epidemic [61]. In Costa Rica, the application of ultra-low volume at the front door and in each room provided three weeks of significant control ($P < 0.05$) [82]. A study in Mexico that used metofluthrin emanators to reduce indoor adult *Aedes aegypti* abundance reported substantial reductions in abundance rate ratios for total *Aedes aegypti*, female abundance, and females that contained blood meals (2.5, 2.4, and 2.3-times fewer mosquitoes respectively; $P < 0.001$) [105].

Insecticide spraying led to a decline in *Aedes* mosquito density and incidence of ABIDs. The reported beneficial effect is up to seven months; thus, data on the longterm effect is lacking. The usage of heavy equipment led to more beneficial effects than portable equipment.

Biological control

Two studies assessed the effect of usage of larvivorous fish in mosquito breeding sites in Latin America. In Brazil, usage of the fish *Betta splendens* led to a reduction in the presence of immature forms of *Aedes aegypti* mosquitoes (70.4% [January 2001] to 7.4 % [January 2002] to 0.2% [December 2002]) [38]. In Mexico, larvivorous fish in breeding sites was associated with lower levels of recent dengue virus infection in children aged 3–9 years (OR 0.64; 95% CI 0.45–0.91) [101]. Another study in Brazil released *Wolbachia*-infected eggs as an intervention. The study reported a successful invasion and long-term (the post-release phase, spanning mid-January 2017 to December 2019) establishment of the bacterium across the study site [63]. The study results support the view that *Wolbachia*-infected mosquitoes have low susceptibility to dengue and Zika virus infections, with reduced viral replication and dissemination to humans [63]. The evidence above highlights biological control measures' beneficial and longterm effects on *Aedes* populations.

Environmental management

Scheduled and periodic container washing led to a decline in *Aedes* (larvae) index in Peru [111]. In Venezuela, no significant difference in reducing the Breteau index (0.84 with a 95% CI: −8.94–10.62 and pupae per person index: −0.023 and a 95% CI: −0.749–0.703) was observed after covering drums with insecticide-treated nets [119]. A study in Brazil indicated that a long-term decrease in adult female population density was achieved only when water tanks and metal drums were covered with nylon net [44]. Another study conducted in Brazil that placed concrete in the bottom of storm drains indicated that after the intervention, water accumulated in 5 (9.6%) of the storm drains ($P < 0.001$), none (0.0%) had immature forms of *Aedes* species ($P < 0.001$), and 3 (5.8%) contained adults' mosquitoes ($P = 0.039$) [58]. Covering only the most productive breeding sites of *Aedes Aegypti* with netted lids also produced beneficial results [64]. The positive benefits of the prevention of water retention were also observed in a study conducted in Colombia [80].

Another study conducted in Colombia assessed the effectiveness of using long-lasting insecticide-treated curtains alone or in combination with container covers [75]. Long-lasting insecticide-treated curtains alone reduced the Breteau index from fourteen to six in the intervention group (the Breteau index in the control group was eight and reduced to five). A significant reduction in pupae per person index (P = 0.01) was observed when long-lasting insecticide-treated curtains were combined with the usage of container covers [75]. A similar study was conducted in Guatemala. Significant differences were observed when treated materials and other interventions targeting productive breeding sites (e.g., larviciding with temephos, elimination of breeding sites) were combined. The combined approach led to significant differences in reductions in the total number of pupae (P = 0.04), the house index (P = 0.01), pupae per person and the Breteau indices (P = 0.05) [85]. A study conducted in Mexico showed the long-term (more than two years) benefits of using insecticide-treated screens combined with treating the most productive breeding sites of *Aedes aegypti* [96]. In Mexico and Venezuela, a combined approach (using insecticide-treated curtains and treating water containers with pyriproxyfen chips or cover water containers) was also applied [107]. In both countries, entomological indices after the intervention were significantly lower than baseline. However, no significant difference between the control and the intervention group was observed due to the spillover effect (an indirect effect on a subject/ area not directly treated by the experiment).

In Mexico, insecticide-treated curtains usage was associated with fewer intradomicile dengue virus transmission [94], dengue virus-infected *Aedes aegypti* female mosquitoes [94, 103], and *Aedes aegypti* mosquitoes' abundance [97, 102, 103, 106]. In contrast with the benefits reported in the studies that used insecticide-treated curtains, a study conducted in Peru indicated that despite the widespread use of treated curtains, individuals living in the intervention area were at greater risk of seroconverting to dengue virus (average seroconversion rate of 50.6 per 100 person-years CI: 29.9–71.9), while those in the control

area had an average seroconversion rate of 37.4 per 100 person-years (CI: 15.2–51.7) [115]. A false sense of security may have caused the higher risk of dengue virus exposure observed in the mentioned study.

Two studies conducted in Uruguay distributed plastic bags for collecting unused small containers [116, 117]. One study indicated that the vector densities in the intervention group, on average, increased less than those in the control group (from spring to autumn) after implementing the interventions (collection of small containers and covering of large containers). However, the difference was statistically not significant [116]. In the other study, the average pupae per person index decreased in the intervention group 11 times and in the control group four times ($P < 0.05$). Although the difference was not statistically significant, the container index, house index, and Breteau index decreased in the intervention group more than those in the control clusters [117].

One study in Cuba and Haiti used long-lasting insecticide-treated curtains or bed nets to control *Aedes* mosquitoes [134, 137]. In Cuba, no effect of the insecticide-treated curtains on *Aedes* infestation levels (house index and Breteau index) was observed (study period 18 months) [134]. In contrast, the study in Haiti demonstrated significant differences between the intervention group and the control group. At one month post-intervention (usage of insecticide-treated bed nets), all entomological indices declined (house index in the intervention group declined with 6.7 (95% CI -10.6, -2.7; $P < 0.01$) and Breteau index reduced by 8.4 (95% CI -14.1, -2.6; $P < 0.01$) [137]. At five months, all indices remained low, and some were significantly lower than baseline in the control group [137]. Also, an IgM serosurvey demonstrated a 5.3% decrease (95% CI 5.0-25.5%, $P < 0.01$) in the number of IgM-positive individuals from baseline to the last survey.

In general, environmental management, especially combined approaches (e.g. using insecticide-treated screens and treating the most productive breeding sites), led to beneficial

and even longterm effects (≥ two years). However, it is crucial to be aware that the community perceptions/ participation and negligence of potential mosquito breeding sites can negatively affect the approach mentioned above's effectiveness.

Traps

In Latin America, eight studies that used traps as control measures were identified [37, 41, 42, 49, 53, 66, 71, 72]. Five studies were conducted in Brazil, and three were performed in Colombia. One study conducted in Brazil found that sticky traps (MosquiTRAP) did not reduce the adult *Aedes aegypti* abundance and dengue infections were equally frequent in the intervention and the control group [53]. In contrast with the findings summarised above, other studies that combined a type of insecticide with traps demonstrated more beneficial results. For example, a study in Brazil that used traps with insecticide-treated ovistrip (impregnated with deltamethrin) reported a significant reduction in densities of *Aedes aegypti* for most comparators ($P < 0.01$), as shown by fewer positive containers and pupae/house at the intervention site compared to the control group [37]. Two other studies in Brazil used ovitraps with *Bti* for a massive collection of *Aedes* eggs [41, 42]. Both studies indicated that massive egg collection by using ovitraps with *Bti* can affect the population density of *Aedes* mosquitoes. Two studies in Colombia also used ovitraps with *Bti*, and a significant reduction in entomological indices was observed [66, 71]. The third study conducted in Colombia found that the ovitraps with the highest vector reduction combined deltamethrin/towel/10% hay infusion [72]. Another study performed in Brazil used BG-Sentinel traps for massive trapping of *Aedes* mosquitoes [49]. The findings of the mentioned study indicated that massive trapping with BG-Sentinel traps significantly reduced the abundance of adult female *Aedes aegypti* mosquitoes during the rainy season. However, no effect was observed in the dry season. Also, dengue infections were lower in the area that used the traps compared to the control area; however, this was not statistically significant [49].

Five publications in the Caribbean region (all from Puerto Rico) reported using autocidal gravid ovitraps (AGO traps) as the primary control intervention [140-143, 147]. Two studies reported significant reductions in mosquito density [140, 141]. There were significant reductions in the captures of female *Aedes aegypti* (53–70%) in the study area. The presence of three to four AGO control traps per house (in 81% of the houses) prevented expected outbreaks of *Aedes aegypti* after rains. Mosquito captures in BG-Sentinel, and AGO traps were significantly and positively correlated, indicating that AGO traps are valuable and inexpensive mosquito surveillance devices [140]. One study reported a lower incidence of chikungunya virus infection in the intervention compared to the control group, resulting from tenfold lower mosquito densities in the intervention areas with AGO traps [142]. Two other studies also reported similar results [143, 147].

The evidence on traps suggests that sticky traps are less effective than ovitraps (combined with a type of insecticide) and traps to capture adult mosquitoes. Ovitraps and traps to capture adult mosquitoes led to a significant reduction in entomological indices and a decline in the incidence of ABIDs.

Genetically modified mosquitoes

One study released transgenic male *Aedes aegypti* mosquitoes with the OX513A line to assess the related changes in the distribution of infestation and abundance of *Aedes aegypti* populations six and eighteen months after the intervention in two areas in Brazil [56]. An average suppression of ± 70% of the wild population due to the release of transgenic males was observed in the mentioned study. In one of the areas, the mosquito population remained suppressed for 17 weeks, whereas in the other area, the suppression lasted 32 weeks [56]. The reported results highlight the benefits of using genetically modified mosquitoes in *Aedes* mosquito control.

Health education and community mobilisation/participation

In Latin America, schools were a popular setting to provide health education concerning diseases transmitted by the *Aedes* mosquitoes discussed in this scoping review [35, 69, 76, 78, 79, 87, 88, 92, 95, 99]. The health education interventions implemented at schools increased awareness of *Aede*s biological characteristics [35, 95] and dengue prevention and control practices [69, 87]. Also, infestation rates of immature forms of *Aedes* mosquitoes in schools were reduced [76, 78, 88]. A health education program implemented in Mexico also influenced the parents' behaviour [92]. According to the mentioned study, the entomological indices decreased significantly ($P < 0.05$) in houses in the intervention area, apparently because parents acted on the comments of the children and eliminated or monitored mosquito breeding sites [92]. A study that evaluated a health education campaign's sustainability (two years post-intervention) to prevent dengue in schools reported that the intervention's effects were not sustained. The intervention still affected the KAP of the schoolchildren; however, the results were not statistically significant [79].

Health education campaigns implemented among the community members also showed beneficial results in the Latin American region; (i) improvement of knowledge and reduction of mosquito breeding sites [36, 47, 74] and (ii) reduction of entomological indices [90]. A study that used a learning platform on mobile devices to improve KAP reported significant changes in attitudes and behaviour ($P = 0.032$) concerning actions to prevent arboviral diseases [60]. Another study that disseminated information via mobile phones suggested that repeated exposure to health information encourages householder's uptake of preventive measures against dengue [112]. One study that assessed the impact of mass-media communication campaigns did not show beneficial results [70]. Another study reported that mass communication campaigns influenced the population's KAP [135]. However, information gaps continue to exist, and actions beyond just providing information are required for better results. A study that combined mass communication campaigns with a school and museum-based

educational program reported that exposure to the intervention was associated with increased knowledge about dengue. [138]. Also, the intervention was associated with an increased proportion of tires protected from rain and a decreased proportion of water storage containers positive for mosquito larvae [138].

A health education campaign based on an eco-health approach, focusing on community mobilisation, house inspection, and covering water containers with insecticide-treated aluminium covers showed an average decrease between 0.12 (-0.25 - 0.01) and 0.26 (-0.42 - -0.10) cases of dengue daily (1.82 cases per week or 7.8 cases per month or 95 cases per year) [81]. The benefits of health education campaigns focussing on community mobilisation and participation have been demonstrated in other study settings in the Latin American region [52, 65, 67, 77, 100, 108, 110]. Most studies on health education and community mobilisation/ participation in the Caribbean region were conducted in Cuba [121-124, 126-128, 130, 131]. Health education and community mobilisation/ participation led to a decline in entomological indices [121-124, 127, 128, 130], breeding sites [126], and behavioural change [127]. One study attributed the success of the intervention to community involvement in the vector control intervention [131]. The beneficial impact of health education focussing on community mobilisation was also observed in Puerto Rico [145].

The evidence concerning health education campaigns highlights the beneficial effect of health education on KAP of school children/ community members and entomological indices. However, the evidence also suggests that only education provision is not enough to control *Aedes* mosquitoes in the long run. Health education campaigns must include community participation/ mobilisation efforts to be successful.

Integrated interventions

A study that evaluated the impact of the Brazilian national dengue plan (information campaigns, epidemiological surveillance and vector reduction interventions, e.g., usage of larvicide temephos and spraying of insecticide) indicated that the goals concerning the reduction of dengue incidence (50% reduction in dengue cases) and larval infestation (less than 1%) were not achieved in most municipalities [43]. Another study that evaluated Brazil's national dengue control plan attributed the program's lack of success to insufficient household coverage [45]. More positive results were achieved when the national program was extended with the following interventions: ovitraps loaded with *Bti*, source elimination campaigns and indoor collections of adult mosquitoes using aspirators, targeting places considered highly important for virus transmission [48]. After two years of sustained control, a 90% decrease in egg density was registered at one study site. Data from another study site showed a sharp decline in the mosquito population [48]. The incorporation of the community mobilisation concept into the Brazilian national plan was linked with the reduction of the total number of dengue cases between the years 2009 and 2010 [54]. Combining the family health program with the national dengue control program can also provide beneficial results, as observed in a Brazilian study [39]. Significant changes in KAP were achieved, and the house index was reduced (6.9% before and after 4.4%, with a significant difference, $P = 0.040$) [39].

A study in Mexico that combined epidemiological surveillance, environmental management through social mobilisation, and chemical control also demonstrated beneficial results [98]. Statistical significant differences ($p < 0.001$) in the relative abundance of *Aedes aegypti* larvae before and after applying larvicide temephos and removing water containers were reported [98]. For example, in La Paz, the average house index was reduced from 16 - 83% to 0 - 5% after the interventions for three years. Beneficial results were also observed in the positive container index and the Breteau index in La Paz and other areas (Cabo San Lucas and San Jose del Cabo), where the interventions were implemented [98]. The beneficial effects of

combining interventions were also documented in another study conducted in Mexico [93]. Dengue incidence decreased from 81.4% in 2010 to 79.1% in 2011 [93].

Other studies also demonstrated the benefits of combined interventions [32, 40, 50, 62, 83, 84]. In Brazil, one study that combined the application of insecticide temephos, environmental management and health education campaigns did not observe positive results [34]. The lack of effect is possibly caused by negligence in eliminating breeding sites observed in the group that also applied temephos and the dilution of the insecticide in non-removable containers. A study in Nicaragua also did not demonstrate a positive effect of temephos combined with other interventions [109]. In the mentioned study, temephos exposure was not associated with a reduction in any entomological indices, and in some cases, temephos exposure was even associated with higher entomological outcomes [109].

In Cuba, an intervention combining the distribution of new ground-level water tanks and intensive use of an insecticide was conducted [125]. The container index decreased significantly from 0.7% to 0.1% one month after the intervention. Six months later, the mentioned index increased to 2.7% due to the uncovered new water container [125]. In Trinidad, an intervention combining the application of temephos in water containers, fenthion at walls of houses of suspected dengue cases and fogging using malathion failed to achieve the desired target of reducing mosquito densities to below the disease transmission threshold or possibly a Breteau index of five [149].

Another study conducted in Cuba implemented a community-based intervention focussing on (i) training a local task force, (ii) organising community working groups, (iii) enhancing collaboration between the government and the community, (iv) and creating a formal link with the routine vector control programme [129]. This intervention led to a lower attack rate of dengue fever (8.5 per 1000 inhabitants) in the intervention group compared to the control group (38.1 per 1000 inhabitants). A study in Puerto Rico reported a reduction in mosquito

density after implementing an intervention that consisted of environmental management, usage of larvicide (Altosid Pro-G), and placement of three AGO traps in the backyards of houses [144]. Density changed from 27.7 mosquitoes/trap/week before to 2.1 after intervention (92.4% reduction), whereas after treating the original control area (cross-over), density changed from 22.4 to 3.5 (84.3% reduction) [144]. The mentioned results were confirmed by another publication [146].

The evidence presented above highlights the importance of combining interventions to mitigate ABIDs. An integrated approach can reduce entomological indices and the incidence of ABIDs. However, an integrated approach can also fail to achieve the desired effect if the community is not involved, mosquito breeding sites are not properly removed or treated, and insecticide is ineffective.

Challenges and lessons learned

Another objective of this scoping review was to describe the challenges and lessons learned reported in the publications. The challenges and lessons learned are presented here by the following key themes: (i) knowledge concerning *Aedes* control and ecological adaptability, (ii) resources and capacity, and (ii) infrastructure and context of the community.

Knowledge concerning *Aedes* control and ecological adaptability

The first step towards *Aedes* control is understanding the mosquitoes' basic biological and ecological characteristics. In most included studies, lack of community involvement, motivation, and community and health workers' understanding of *Aedes’s* control immediate and long-term impact have been reported as significant barriers to successful program implementation [36, 67, 125]. Deciding on the methods for controlling calls for a deeper understanding of the operational aspects of mosquito control (the local conditions) and the ecological adaptability of the *Aedes* mosquitoes. For example, one study observed the evolution of mosquito resistance due to intense insecticide application [50]. Nowadays, *Aedes*

aegypti can also use cryptic and non-clear aquatic habitats as oviposition sites. This change in the ecology of the mosquito can affect the results of the traditional surveillance and control methods. Furthermore, it is essential to understand community perceptions, control behaviour, and preferred control interventions [53]. For example, using a chemical substance such as temephos was linked with an increased risk of dengue infection, resulting from a false sense of security engendered by knowledge of pesticides in water storage [108].

Resources and capacity

Insufficient resources, specifically human resources, to perform mosquito control is also a potential contributing factor that hinders mosquito control intervention's success [45]. For a sustainable intervention, the key is to have the political will to provide adequate and uninterrupted supplies [79]. A study in Guatemala reported financial constraints and scarcity of vehicles, fuel, and personnel to obstruct the sustainability of interventions [86]. Another study indicated that providing adequate basic utility services (e.g., garbage collection) to the communities could have a major bearing on the sustainability of the community-based mosquito control intervention, facilitating the successful implementation of the *Aedes* control program [123]. Targeting long-term behaviour change among the community members is imperative for sustainability. Actively involving household members in basic mosquito control measures is a good start to avoid dependency on the program implementer and ensure program sustainability [116]. Mosquito control is a shared responsibility and requires coordination and support from all relevant sectors [46, 86]. Programs need to be evaluated, and networks among the stakeholders need to be maintained.

Infrastructure and context of the community

Important factors that might influence the effectiveness of *Aedes* mosquito control are the housing and surrounding conditions, methods of waste disposal, and socioeconomic status of the community [57]. Hence, detailed assessments of all public services, such as waste management, sewerage systems, etc., should be made to inform entomological surveys of

Aedes mosquitoes [139]. The success of *Aedes* control intervention is also dependent on the socio and geopolitical condition of the community. In areas where community organisations are fragile or non-existent or in a conflicted community with a history of violence and unrest, social participation is slim to none [52, 108].

Strategies employed or recommended to improve the effectiveness of *Aedes* control interventions

The last objective of this scoping review is to list the employed or recommended strategies to improve the effectiveness of *Aedes* control. The recommendations are listed below:

(i) Community participation enhances the success of *Aedes* control interventions [77, 84, 110, 117, 121, 122, 145]. Schools are an important setting that needs to be considered when designing community participation strategies since beneficial results have been booked with health education campaigns employed at schools [35, 48, 78, 99].

(ii) The use of a multi-sectoral approach or inter-sectorial integration [39, 88, 89, 126, 127, 138], integrated *Aedes* control approaches [96, 110, 146], and prioritizing ABIDs (local government providing financial and administrative support) [120, 121] were stated as common reasons for the success of an intervention.

(iii) The effectiveness of the intervention improved when qualified staff planned and implemented an intervention [45, 67], and the community accepted the intervention technique and believed in its effectiveness [106].

(iv) Interventions were effective when health education messages were repeated throughout the intervention [112].

(v) The health workforce should be trained and capable of identifying the most productive breeding sites [73]. The workforce should keep standard operating procedures for entomological and epidemiological surveillance, and data should be consistent and follow standardised measurement tools for inspection activities

[67, 127]. Furthermore, the workforce should assess the short term and long-term impact of the *Aedes* control program because these assessments facilitate program implementation in the long run and sustainability. To assess immediate outcomes, periodic monitoring and evaluation are recommended to inform what works and needs to be amended [67]. Having a list of program indicators could be beneficial for program implementers to quantify and draw inferences on the outcomes against the input, which can be helpful for the allocation of resources.

(vi) It is also important to consider conducting a cost-benefit analysis as the intervention progresses, considering the direct cost of implementing the intervention and the potential consequences [90].

(vii) Lastly, the workforce should consider factors (e.g., meteorological conditions such as wind speed, direction, or temperature) contributing to differences in larval mortality related to application data [148]. Another factor that can invariably affect implementation if not counter for is the seasonal variation of pupal productivity [85]. Therefore, a baseline pupal survey should be repeated every season to take seasonal variations of *Aedes* populations into account when designing an intervention.

Discussion

This scoping review synthesises evidence concerning the effectiveness of *Aedes aegypti* and *Aedes albopictus* prevention and control interventions performed in LAC in the last twenty-one years (2000-2021). To our knowledge, this review provides the most updated overview of evidence regarding the effectiveness of *Aedes* prevention and control interventions in the region mentioned above. Most of the data were from studies in Brazil, followed by Mexico and Colombia. Cuba has most of the publications in the Caribbean region, followed by Puerto Rico and Trinidad. The synthesised data showed that combined interventions were more effective than a single approach. Another review supported our findings by reporting that it is unlikely

that any single intervention will be fully effective against *Aedes* mosquitoes and encourages the development and usage of an IVM program to control the mentioned mosquito species effectively [153].

Although our scoping review has a broader scope than a previously published systematic review and meta-analysis on the effectiveness of interventions for the control of *Aedes aegypti* in the LAC region [154], the same conclusions concerning the beneficial effect of an IVM program are drawn. An IVM program has been pointed out as the most effective approach to reducing *Aedes aegypti* immature forms and adult mosquitoes [154, 155]. The WHO and the PAHO insist that the IVM approach is the solution to mitigate ABID transmissions [15]. However, many factors such as insufficient funding, resources, workforce, and political priorities (e.g., prioritising SARS-CoV-2 over ABIDs) obstruct the implementation and maintenance of the IVM program nowadays. On one hand, it is easy to recommend developing and implementing an IVM program. On the other hand, maintaining a cycle of periodic implementation of the program is challenging—this leaves the door open for future epidemics.

The most important question that remains is, how can we sustain the effectiveness of the IVM program? One way to counter this is through routine monitoring and evaluation, building the capacity of the community, building a network of relevant stakeholders (local and international), and taking the local challenges of the health system and the community into account when designing the IVM program. However, all these would require multisectoral and multi-disciplinary team engagement and active community participation. An IVM without community participation is not sustainable [154, 156].

The effectiveness of the interventions included in this review was reduced by the following factors, the spillover effect [75, 107, 137], small sample size [116], providing educational strategies to deploy control measures in the control group [67], parallel government campaigns [75, 84, 85, 87, 128, 136], insecticide resistance [149] or media freedom to change or stop the

distribution of information [70]. Also, most interventions were followed for a short period, which led to limited evidence on the sustainability of the employed interventions. Future research should focus on *Aedes Albopictus* and its active role in transmitting ABIDs. Also, researchers might consider studying the impact of an IVM approach on various ABIDs (e.g., dengue and Zika combined) since this evidence is also missing in the scientific literature. Furthermore, more research and dialogue on the barriers of an IVM program are needed to work toward more sustainable programs. This scoping review also aimed to synthesise evidence on how other countries in the LAC region dealt with their challenges, but this information was not available in all the included publications. Therefore, we recommend future research to address this topic to provide more practical advice to countries struggling with their *Aedes* control approaches. Lastly, we encourage countries with more advanced techniques and qualified workforce to support countries with less developed health systems.

Limitations and strengths

Since the quality of the included studies was not assessed in this scoping review, the included studies' results need to be interpreted with caution. Quality assessment is essential for systematic reviews and meta-analysis, but it is not required for scoping reviews [25]. The main strength of this scoping review is the extensive literature, grey literature and reference list search that was conducted to find relevant publications. Another strength is the methodology used; each phase in data search, screening and extraction was piloted and cross-checked to reduce the chance of bias.

Conclusion

Prolonged community participation is key to sustaining the effectiveness of *Aedes* control interventions. The integrated *Aedes* control approach was more effective than a single strategy. Although it is known that an integrated approach in combination with community participation can be effective, many barriers still obstruct the development and implementation of such an approach, jeopardising the health system's preparedness and performance. Future

research and recommendations need to focus more on IVM program implementation and maintenance barriers since this information is lacking in scientific literature.

Acknowledgements

We want to thank both the editors and reviewers for their insightful comments on the article.

Funding

Funding was provided by the Dutch Research Council (NWO,https://www.nwo.nl/onderzoek-en-resultaten/onderzoeksprojecten) for the project entitled "Public health impact of chronic chikungunya illness and performance/utilisation of the health care system in the face of arboviral (dengue, chikungunya, Zika) epidemics in Curacao" Acronym: ARBOCARIB (NWO grant ALWCA.2016.021). The funders had no role in study design, data collection and analysis, decision to publish, or preparation of the manuscript.

References

1. Harbach R, Howard TM. Index of currently recognized mosquito species (Diptera: Culicidae). European Mosquito Bulletin. 2007;23:1-66.

2. WHO. Vector-borne disease [Internet]. 2021 [cited 2021 28 December]. Available from: https://www.who.int/heli/risks/vectors/vector/en/.

3. ECDC. Mosquito factsheets [Internet]. 2021 [cited 2021 28 December]. Available from: https://www.ecdc.europa.eu/en/disease-vectors/facts/mosquito-factsheets.

4. Jones KE, Patel NG, Levy MA, Storeygard A, Balk D, Gittleman JL, et al. Global trends in emerging infectious diseases. Nature. 2008;451(7181):990-3. doi: 10.1038/nature06536. PubMed PMID: 18288193; PubMed Central PMCID: PMCPMC5960580.

5. Guzman MG, Gubler DJ, Izquierdo A, Martinez E, Halstead SB. Dengue infection. Nat Rev Dis Primers. 2016;2:16055. Epub 20160818. doi: 10.1038/nrdp.2016.55. PubMed PMID: 27534439.

6. Tapia-Conyer R, Betancourt-Cravioto M, Mendez-Galvan J. Dengue: an escalating public health problem in Latin America. Paediatr Int Child Health. 2012;32 Suppl 1:14-7. doi: 10.1179/2046904712Z.00000000046. PubMed PMID: 22668444; PubMed Central PMCID: PMCPMC3381443.

7. CARPHA. Overview [Internet]. 2021 [cited 2021 28 December]. Available from: https://www.carpha.org/What-We-Do/VBD/Overview.

8. CDC. Mosquito-Borne Diseases [Internet]. 2016 [cited 2021 28 December]. Available from: https://www.cdc.gov/niosh/topics/outdoor/mosquito-borne/default.html.

9. Powell JR. Mosquito-Borne Human Viral Diseases: Why *Aedes aegypti*? Am J Trop Med Hyg. 2018;98(6):1563-5. Epub 20180315. doi: 10.4269/ajtmh.17-0866. PubMed PMID: 29557341; PubMed Central PMCID: PMCPMC6086192.

10. McKenzie BA, Wilson AE, Zohdy S. *Aedes albopictus* is a competent vector of Zika virus: A meta-analysis. PLoS One. 2019;14(5):e0216794. Epub 20190521. doi: 10.1371/journal.pone.0216794. PubMed PMID: 31112569; PubMed Central PMCID: PMCPMC6528984.

11. Reiter P, Fontenille D, Paupy C. *Aedes albopictus* as an epidemic vector of chikungunya virus: another emerging problem? Lancet Infect Dis. 2006;6(8):463-4. doi: 10.1016/S1473-3099(06)70531-X. PubMed PMID: 16870524.

12. PAHO. PAHO Advisory: *Aedes albopictus* in the Caribbean [Internet]. 1993 [cited 2021 28 December]. Available from: https://iris.paho.org/handle/10665.2/32198.

13. Ferreira-de-Lima VH, Câmara DCP, Honório NA, Lima-Camara TN. The Asian tiger mosquito in Brazil: Observations on biology and ecological interactions since its first detection in 1986. Acta Tropica. 2020;205:105386. doi: https://doi.org/10.1016/j.actatropica.2020.105386.

14. Sridhar S, Luedtke A, Langevin E, Zhu M, Bonaparte M, Machabert T, et al. Effect of Dengue Serostatus on Dengue Vaccine Safety and Efficacy. N Engl J Med. 2018;379(4):327-40. Epub 20180613. doi: 10.1056/NEJMoa1800820. PubMed PMID: 29897841.

15. WHO. Global Strategic Framework for Integrated Vector Management [Internet]. 2004 [cited 2021 28 December]. Available from: http://apps.who.int/iris/bitstream/handle/10665/68624/WHO_CDS_CPE_PVC_2004_10.pdf?sequence=1.

16. Dos SRC, van de Burgwal LHM, Regeer BJ. Overcoming challenges for designing and implementing the One Health approach: A systematic review of the literature. One Health. 2019;7:100085. Epub 20190318. doi: 10.1016/j.onehlt.2019.100085. PubMed PMID: 31016220; PubMed Central PMCID: PMCPMC6475629.

17. Mulderij-Jansen V, Gerstenbluth I, Duits A, Tami A, Bailey A. Evaluating and strengthening the health system of Curaçao to improve its performance for future outbreaks of vector-borne diseases. Parasit Vectors. 2021;14(1):500. Epub 20210926. doi: 10.1186/s13071-021-05011-x. PubMed PMID: 34565464; PubMed Central PMCID: PMCPMC8474927.

18. Lowe R, Barcellos C, Brasil P, Cruz OG, Honorio NA, Kuper H, et al. The Zika Virus Epidemic in Brazil: From Discovery to Future Implications. Int J Environ Res Public Health.

2018;15(1). Epub 20180109. doi: 10.3390/ijerph15010096. PubMed PMID: 29315224; PubMed Central PMCID: PMCPMC5800195.

19. Levi LI, Vignuzzi M. Arthritogenic Alphaviruses: A Worldwide Emerging Threat? Microorganisms. 2019;7(5). Epub 20190514. doi: 10.3390/microorganisms7050133. PubMed PMID: 31091828; PubMed Central PMCID: PMCPMC6560413.

20. ECDC. Aedes aegypti - Factsheet for experts [Internet]. 2021 [cited 2021 28 December]. Available from: https://www.ecdc.europa.eu/en/disease-vectors/facts/mosquito-factsheets/aedes-aegypti.

21. ECDC. Aedes albopictus - Factsheet for experts [Internet]. 2021 [cited 2021 28 December]. Available from: https://www.ecdc.europa.eu/en/disease-vectors/facts/mosquito-factsheets/aedes-albopictus.

22. Achee NL, Gould F, Perkins TA, Reiner RC, Jr., Morrison AC, Ritchie SA, et al. A critical assessment of vector control for dengue prevention. PLoS Negl Trop Dis. 2015;9(5):e0003655. Epub 20150507. doi: 10.1371/journal.pntd.0003655. PubMed PMID: 25951103; PubMed Central PMCID: PMCPMC4423954.

23. Arksey H, O'Malley L. Scoping studies: towards a methodological framework. International Journal of Social Research Methodology. 2005;8(1):19-32. doi: 10.1080/1364557032000119616.

24. Levac D, Colquhoun H, O'Brien KK. Scoping studies: advancing the methodology. Implement Sci. 2010;5:69. Epub 20100920. doi: 10.1186/1748-5908-5-69. PubMed PMID: 20854677; PubMed Central PMCID: PMCPMC2954944.

25. Peters M, Godfrey C, McInerney P, Soares CB, Khalil H, D. P. The Joanna Briggs Institute Reviewers' Manual 2015: Methodology for JBI Scoping Reviews [Internet]. 2015 [cited 2021 28 December]. Available from: https://nursing.lsuhsc.edu/JBI/docs/ReviewersManuals/Scoping-.pdf.

26. Tricco AC, Lillie E, Zarin W, O'Brien KK, Colquhoun H, Levac D, et al. PRISMA Extension for Scoping Reviews (PRISMA-ScR): Checklist and Explanation. Ann Intern Med. 2018;169(7):467-73. Epub 20180904. doi: 10.7326/M18-0850. PubMed PMID: 30178033.

27. Bank W. Latin America & Caribbean [Internet]. 2021 [cited 2021 28 December]. Available from: https://data.worldbank.org/region/latin-america-and-caribbean.

28. McGowan J, Sampson M, Salzwedel DM, Cogo E, Foerster V, Lefebvre C. PRESS Peer Review of Electronic Search Strategies: 2015 Guideline Statement. J Clin Epidemiol. 2016;75:40-6. Epub 20160319. doi: 10.1016/j.jclinepi.2016.01.021. PubMed PMID: 27005575.

29. Ouzzani M, Hammady H, Fedorowicz Z, Elmagarmid A. Rayyan-a web and mobile app for systematic reviews. Syst Rev. 2016;5(1):210. Epub 20161205. doi: 10.1186/s13643-016-0384-4. PubMed PMID: 27919275; PubMed Central PMCID: PMCPMC5139140.

30. Page MJ, McKenzie JE, Bossuyt PM, Boutron I, Hoffmann TC, Mulrow CD, et al. The PRISMA 2020 statement: an updated guideline for reporting systematic reviews. BMJ. 2021;372:n71. Epub 20210329. doi: 10.1136/bmj.n71. PubMed PMID: 33782057; PubMed Central PMCID: PMCPMC8005924.

31. Masuh H, De Licastro SA, Lopez PA, Vega C, Zerba E. Field evaluation of a smoke-generating formulation containing beta-cypermethrin against the dengue vector in Argentina. J Am Mosq Control Assoc. 2003;19(1):53-7. PubMed PMID: 12674535.

32. Gurtler RE, Garelli FM, Coto HD. Effects of a five-year citywide intervention program to control *Aedes aegypti* and prevent dengue outbreaks in northern Argentina. PLoS Negl Trop Dis. 2009;3(4):e427. Epub 20090428. doi: 10.1371/journal.pntd.0000427. PubMed PMID: 19399168; PubMed Central PMCID: PMCPMC2669131.

33. Harburguer L, Beltran G, Goldberg L, Goldberg L, Zerba E, Licastro S, et al. A new strategy for *Aedes aegypti* (Diptera: Culicidae) control with community participation using a new fumigant formulation. J Med Entomol. 2011;48(3):577-83. doi: 10.1603/me10074. PubMed PMID: 21661319.

34. Camargo Donalisio MR, Leite OF, Mayo RC, Pinheiro Alves MJC, de Souza A, Rangel O, et al. Use of Temephos for Control of Field Population of *Aedes aegypti* in Americana Sao Paulo, Brazil. 2002.

35. Madeira NG, Macharelli CA, Pedras JF, Delfino MC. Education in primary school as a strategy to control dengue. Rev Soc Bras Med Trop. 2002;35(3):221-6. doi: 10.1590/s0037-86822002000300004. PubMed PMID: 12045814.

36. Chiaravalloti Neto F, Fiorin AM, Conversani DT, Cesarino MB, Barbosa AA, Dibo MR, et al. Dengue vector control and community participation in Catanduva, Sao Paulo State, Brazil. Cad Saude Publica. 2003;19(6):1739-49. Epub 20040303. doi: 10.1590/s0102-311x2003000600018. PubMed PMID: 14999340.

37. Perich MJ, Kardec A, Braga IA, Portal IF, Burge R, Zeichner BC, et al. Field evaluation of a lethal ovitrap against dengue vectors in Brazil. Med Vet Entomol. 2003;17(2):205-10. doi: 10.1046/j.1365-2915.2003.00427.x. PubMed PMID: 12823838.

38. Pamplona Lde G, Lima JW, Cunha JC, Santana EW. Evaluation of the impact on *Aedes aegypti* infestation in cement tanks of the municipal district of Caninde, Ceara, Brazil after using the Betta splendens fish as an alternative biological control. Rev Soc Bras Med Trop. 2004;37(5):400-4. Epub 20040903. doi: 10.1590/s0037-86822004000500006. PubMed PMID: 15361957.

39. Chiaravalloti Neto F, Barbosa AA, Cesarino MB, Favaro EA, Mondini A, Ferraz AA, et al. Dengue control in an urban area of Brazil: impact of the Family Health Program on traditional control. Cad Saude Publica. 2006;22(5):987-97. Epub 20060428. doi: 10.1590/s0102-311x2006000500011. PubMed PMID: 16680351.

40. Favier C, Degallier N, Vilarinhos Pde T, de Carvalho Mdo S, Yoshizawa MA, Knox MB. Effects of climate and different management strategies on *Aedes aegypti* breeding sites: a longitudinal survey in Brasilia (DF, Brazil). Trop Med Int Health. 2006;11(7):1104-18. doi: 10.1111/j.1365-3156.2006.01653.x. PubMed PMID: 16827711.

41. Regis L, Monteiro AM, Melo-Santos MA, Silveira JC, Jr., Furtado AF, Acioli RV, et al. Developing new approaches for detecting and preventing *Aedes aegypti* population outbreaks: basis for surveillance, alert and control system. Mem Inst Oswaldo Cruz. 2008;103(1):50-9. doi: 10.1590/s0074-02762008000100008. PubMed PMID: 18368236.

42. Santos MAV. *Aedes aegypti* (Diptera: Culicidae): estudos populacionais e estratégias integradas para controle vetorial em municípios da Região metropolitana do Recife, no período de 2001 a 2007. 2008.

43. Pessanha JE, Caiaffa WT, Cesar CC, Proietti FA. Evaluation of the Brazilian National Dengue Control Plan. Cad Saude Publica. 2009;25(7):1637-41. doi: 10.1590/s0102-311x2009000700024. PubMed PMID: 19578587.

44. Maciel-de-Freitas R, Lourenco-de-Oliveira R. Does targeting key-containers effectively reduce *Aedes aegypti* population density? Trop Med Int Health. 2011;16(8):965-73. Epub 20110523. doi: 10.1111/j.1365-3156.2011.02797.x. PubMed PMID: 21605290.

45. Costa NM. Avaliação das ações de combate ao vetor, previstas no plano nacional de controle a dengue, no município de Caruaru, período de 2008 a 2009 2011.

46. Luz PM, Vanni T, Medlock J, Paltiel AD, Galvani AP. Dengue vector control strategies in an urban setting: an economic modelling assessment. Lancet. 2011;377(9778):1673-80. Epub 20110503. doi: 10.1016/S0140-6736(11)60246-8. PubMed PMID: 21546076; PubMed Central PMCID: PMCPMC3409589.

47. da Silva P, Martins AM, Schall VT. Cooperation between health personnel and schools for the identification and control of dengue. Rev Bras Promoc Saude. 2013;26(3): 404-11. doi: http://dx.doi.org/10.5020/2948.

48. Regis LN, Acioli RV, Silveira JC, Jr., Melo-Santos MA, Souza WV, Ribeiro CM, et al. Sustained reduction of the dengue vector population resulting from an integrated control strategy applied in two Brazilian cities. PLoS One. 2013;8(7):e67682. Epub 20130703. doi: 10.1371/journal.pone.0067682. PubMed PMID: 23844059; PubMed Central PMCID: PMCPMC3701045.

49. Degener CM, Eiras AE, Azara TM, Roque RA, Rosner S, Codeco CT, et al. Evaluation of the effectiveness of mass trapping with BG-sentinel traps for dengue vector control: a cluster randomized controlled trial in Manaus, Brazil. J Med Entomol. 2014;51(2):408-20. doi: 10.1603/me13107. PubMed PMID: 24724291.

50. Maciel-de-Freitas R, Valle D. Challenges encountered using standard vector control measures for dengue in Boa Vista, Brazil. Bull World Health Organ. 2014;92(9):685-9. Epub 2014/07/24. doi: 10.2471/BLT.13.119081. PubMed PMID: 25378760.

51. Abad-Franch F, Zamora-Perea E, Ferraz G, Padilla-Torres SD, Luz SL. Mosquito-disseminated pyriproxyfen yields high breeding-site coverage and boosts juvenile mosquito mortality at the neighborhood scale. PLoS Negl Trop Dis. 2015;9(4):e0003702. Epub 20150407. doi: 10.1371/journal.pntd.0003702. PubMed PMID: 25849040; PubMed Central PMCID: PMCPMC4388722.

52. Caprara A, Lima JW, Peixoto AC, Motta CM, Nobre JM, Sommerfeld J, et al. Entomological impact and social participation in dengue control: a cluster randomized trial in Fortaleza, Brazil. Trans R Soc Trop Med Hyg. 2015;109(2):99-105. doi: 10.1093/trstmh/tru187. PubMed PMID: 25604760; PubMed Central PMCID: PMCPMC4299523.

53. Degener CM, de Azara TM, Roque RA, Rosner S, Rocha ES, Kroon EG, et al. Mass trapping with MosquiTRAPs does not reduce *Aedes aegypti* abundance. Mem Inst Oswaldo Cruz. 2015;110(4):517-27. Epub 20150428. doi: 10.1590/0074-02760140374. PubMed PMID: 25946154; PubMed Central PMCID: PMCPMC4501416.

54. Alecrim JS, Cotta A, de Castro JM. Relationship between the actions for the dengue fever prevention and the impact caused on the cases
reported between 2009 and 2010. J HEALTH Sci. 2016;18. doi: https://doi.org/10.17921/2447-8938.2016v18n4p286-90.

55. Abad-Franch F, Zamora-Perea E, Luz SL. Mosquito-Disseminated Insecticide for Citywide Vector Control and Its Potential to Block Arbovirus Epidemics: Entomological Observations and Modeling Results from Amazonian Brazil. PLoS Med. 2017;14(1):e1002213. Epub 20170117. doi: 10.1371/journal.pmed.1002213. PubMed PMID: 28095414; PubMed Central PMCID: PMCPMC5240929.

56. Garziera L, Pedrosa MC, de Souza FA, Gómez M, Moreira MB, Virginio JF, et al. Effect of interruption of over-flooding releases of transgenic mosquitoes over wild population of

Aedes aegypti: two case studies in Brazil. Entomologia Experimentalis et Applicata. 2017;164(3):327-39. doi: https://doi.org/10.1111/eea.12618.

57. Piovezan R, Acorinthe JPO, Visockas A, de Azevedo TS, Von Zuben CJ. Effect of heavy-equipment aided environmental nebulization on *Aedes aegypti* vectors of Dengue, Zika and Chikungunya in Sao Paulo, Brazil. Bull Entomol Res. 2017;107(4):478-86. Epub 20161215. doi: 10.1017/S0007485316001115. PubMed PMID: 27974058.

58. Souza RL, Mugabe VA, Paploski IAD, Rodrigues MS, Moreira P, Nascimento LCJ, et al. Effect of an intervention in storm drains to prevent *Aedes aegypti* reproduction in Salvador, Brazil. Parasit Vectors. 2017;10(1):328. Epub 20170711. doi: 10.1186/s13071-017-2266-6. PubMed PMID: 28697811; PubMed Central PMCID: PMCPMC5505146.

59. Santos EM, Regis LN, Silva-Filha MH, Barbosa RM, Melo-Santos MAV, Gomes TC, et al. The effectiveness of a combined bacterial larvicide for mosquito control in an endemic urban area in Brazil. Biological Control. 2018;121. doi: 10.1016/j.biocontrol.2018.03.006.

60. Abel Mangueira FF, Smania-Marques R, Dutra Fernandes I, Alves Albino V, Olinda R, Acácia Santos-Silva T, et al. The prevention of arboviral diseases using mobile devices: a preliminary study of the attitudes and behaviour change produced by educational interventions. Trop Med Int Health. 2019;24(12):1411-26. Epub 20191110. doi: 10.1111/tmi.13316. PubMed PMID: 31626375.

61. Marini G, Guzzetta G, Marques Toledo CA, Teixeira M, Rosa R, Merler S. Effectiveness of Ultra-Low Volume insecticide spraying to prevent dengue in a non-endemic metropolitan area of Brazil. PLoS Comput Biol. 2019;15(3):e1006831. Epub 20190308. doi: 10.1371/journal.pcbi.1006831. PubMed PMID: 30849074; PubMed Central PMCID: PMCPMC6426269.

62. Gonzalez MA, Dilger E, Ronderos MM, Spinelli GR, Courtenay O, Hamilton JGC. Significant reduction in abundance of peridomestic mosquitoes (Culicidae) and Culicoides midges (Ceratopogonidae) after chemical intervention in western Sao Paulo, Brazil. Parasit Vectors. 2020;13(1):549. Epub 20201107. doi: 10.1186/s13071-020-04427-1. PubMed PMID: 33160407; PubMed Central PMCID: PMCPMC7648319.

63. Gesto JSM, Ribeiro GS, Rocha MN, Dias FBS, Peixoto J, Carvalho FD, et al. Reduced competence to arboviruses following the sustainable invasion of Wolbachia into native *Aedes aegypti* from Southeastern Brazil. Scientific Reports. 2021;11(1):10039. doi: 10.1038/s41598-021-89409-8.

64. Romero-Vivas CM, Wheeler JG, Falconar AK. An inexpensive intervention for the control of larval *Aedes aegypti* assessed by an improved method of surveillance and analysis. J Am Mosq Control Assoc. 2002;18(1):40-6. PubMed PMID: 11998929.

65. Luna JE, Chain I, Hernandez J, Clark GG, Bueno A, Escalante R, et al. Social Mobilization Using Strategies of Education and Communication to Prevent Dengue Fever in Bucaramanga, Colombia. Dengue Bulletin. 2004.

66. Ocampo CB, Gonzalez C, Morales CA, Perez M, Wesson D, Apperson CS. Evaluation of community-based strategies for *Aedes aegypti* control inside houses. Biomedica. 2009;29(2):282-97. PubMed PMID: 20128353.

67. Cáceres-Manrique FdM, Angulo-Silva ML, Vesga-Gómez C. Eficacia de la movilización y la participación social para la apropiación o "empoderamiento" (sic.) (empowerment) de las medidas de control del dengue, Comuna Norte, Bucaramanga, 2008-2009. Biomédica. 2010;30(4):539-50. doi: 10.7705/biomedica.v30i4.292.

68. Pacheco-Coral Adel P, Quiñones-Pinzón ML, Serrato-Pomar IM, Rivas-Muñoz FA. Evaluating an Information, Education and Communication (IEC) strategy which was adopted for *Aedes aegypti* control in La Dorada, Colombia. Rev Salud Publica (Bogota). 2010;12(3):380-90. doi: 10.1590/s0124-00642010000300004. PubMed PMID: 21311826.

69. Vesga-Gomez C, Caceres-Manrique Fde M. The efficacy of play-based education in preventing dengue in primary-school children. Rev Salud Publica (Bogota). 2010;12(4):558-69. PubMed PMID: 21340121.

70. Carabali M, Ocampo CB, Toledo ME, Osorio L. Mass communication of dengue surveillance data: effect of an intervention in Guadalajara de Buga, Colombia. Biomedica. 2013;33 Suppl 1:130-41. PubMed PMID: 24652257.

71. Alarcon EP, Segura AM, Rua-Uribe G, Parra-Henao G. Ovitraps evaluation for surveillance and control of *Aedes aegypti* in two urban settlements of Uraba, Antioquia. Biomedica. 2014;34(3):409-24. doi: 10.1590/S0120-41572014000300011. PubMed PMID: 25504128.

72. Quimbayo M, Rúa-Uribe G, Parra-Henao G, Torres C. Evaluation of lethal ovitraps as a strategy for *Aedes aegypti* control. Biomedica. 2014;34(3):473-82. doi: 10.1590/s0120-41572014000300016. PubMed PMID: 25504133.

73. Ocampo CB, Mina NJ, Carabali M, Alexander N, Osorio L. Reduction in dengue cases observed during mass control of *Aedes* (Stegomyia) in street catch basins in an endemic urban area in Colombia. Acta Trop. 2014;132:15-22. Epub 20140102. doi: 10.1016/j.actatropica.2013.12.019. PubMed PMID: 24388794; PubMed Central PMCID: PMCPMC4654410.

74. Escudero-Tamara E, Villareal-Amaris G. Educational intervention for the control of dengue in family environments in a community in Colombia. Rev Peru Med Exp Salud Publica. 2015;32(1):19-25. PubMed PMID: 26102101.

75. Quintero J, Garcia-Betancourt T, Cortes S, Garcia D, Alcala L, Gonzalez-Uribe C, et al. Effectiveness and feasibility of long-lasting insecticide-treated curtains and water container covers for dengue vector control in Colombia: a cluster randomised trial. Trans R Soc Trop Med Hyg. 2015;109(2):116-25. doi: 10.1093/trstmh/tru208. PubMed PMID: 25604762; PubMed Central PMCID: PMCPMC4299530.

76. Vargas SL, Cabezas LV, Jaramillo JF, Ibáñez É, Olano VA. Results of a set of integrated interventions to reduce diarrhea and dengue entomological risk in rural schools in the municipality of Apulo, Cundinamarca, Colombia. Rev salud bosque. 2015;5:7-24.

77. Criado Morales ML, Camacho Martinez YM, Mejia Solano YP, Martinez Caceres H, Cabrera LJ, Chacon DJ, et al. Processes of community participation in the prevention of dengue Barrio Ciudadela del Fonce. Cult cuid enferm 2016;13.

78. Overgaard HJ, Alexander N, Matiz MI, Jaramillo JF, Olano VA, Vargas S, et al. A Cluster-Randomized Controlled Trial to Reduce Diarrheal Disease and Dengue Entomological

Risk Factors in Rural Primary Schools in Colombia. PLoS Negl Trop Dis. 2016;10(11):e0005106. Epub 20161107. doi: 10.1371/journal.pntd.0005106. PubMed PMID: 27820821; PubMed Central PMCID: PMCPMC5098800.

79. Jaramillo JF, Vargas S, Sarmiento-Senior D, Giraldo P. Sustainability of interventions to prevent dengue and diarrhea in rural schools in two municipalities in Colombia: a two-year post-project evaluation. Cad Saude Publica. 2018;34(10):e00189017. Epub 20181022. doi: 10.1590/0102-311X00189017. PubMed PMID: 30365746.

80. Ceron-Hernandez VA, Peña MR, Alexander N. Randomized Trial of a Modification of Rainwater Street Catch Basins to Physically Control the *Aedes aegypti* Dengue Mosquito Vector. Ingenieria y Universidad. 2020;24. doi: 10.11144/Javeriana.iued24.rtmr.

81. Quintero J, Ronderos Pulido N, Logan J, Ant T, Bruce J, Carrasquilla G. Effectiveness of an intervention for *Aedes aegypti* control scaled-up under an inter-sectoral approach in a Colombian city hyper-endemic for dengue virus. PLoS One. 2020;15(4):e0230486. Epub 20200401. doi: 10.1371/journal.pone.0230486. PubMed PMID: 32236142; PubMed Central PMCID: PMCPMC7112230.

82. Perich MJ, Rocha NO, Castro AL, Alfaro AW, Platt KB, Solano T, et al. Evaluation of the efficacy of lambda-cyhalothrin applied by three spray application methods for emergency control of *Aedes aegypti* in Costa Rica. J Am Mosq Control Assoc. 2003;19(1):58-62. PubMed PMID: 12674536.

83. Rodríguez R, Marquetti MdC, Ríos M. Larval indexes of *Aedes aegypti* before and after control intervention in Limón, Costa Rica. Revista Cubana de Medicina Tropical. 2009;61.

84. Mitchell-Foster K, Ayala EB, Breilh J, Spiegel J, Wilches AA, Leon TO, et al. Integrating participatory community mobilization processes to improve dengue prevention: an eco-bio-social scaling up of local success in Machala, Ecuador. Trans R Soc Trop Med Hyg. 2015;109(6):419. Epub 20150418. doi: 10.1093/trstmh/trv028. PubMed PMID: 25890933; PubMed Central PMCID: PMCPMC7297300.

85. Rizzo N, Gramajo R, Escobar MC, Arana B, Kroeger A, Manrique-Saide P, et al. Dengue vector management using insecticide treated materials and targeted interventions on

productive breeding-sites in Guatemala. BMC Public Health. 2012;12:931. Epub 20121030. doi: 10.1186/1471-2458-12-931. PubMed PMID: 23110515; PubMed Central PMCID: PMCPMC3533994.

86. Ulibarri G, Betanzos A, Betanzos M, Rojas JJ. Preliminary results on the control of *Aedes* spp. in a remote Guatemalan community vulnerable to dengue, chikungunya and Zika virus: community participation and use of low-cost ecological ovillantas for mosquito control. F1000Res. 2016;5:598. Epub 20160407. doi: 10.12688/f1000research.8461.3. PubMed PMID: 28105304; PubMed Central PMCID: PMCPMC5225411.

87. Avila Montes GA, Martinez M, Sherman C, Fernandez Cerna E. Evaluation of an educational module on dengue and *Aedes aegypti* for schoolchildren in Honduras. Rev Panam Salud Publica. 2004;16(2):84-94. doi: 10.1590/s1020-49892004000800003. PubMed PMID: 15357933.

88. Avila Montes GA, Araujo R, Leontsini E, Orellana Herrera G, Fernandez Cerna E. A school program for dengue control in Honduras: from knowledge to action. Rev Panam Salud Publica. 2012;31(6):518-22. doi: 10.1590/s1020-49892012000600011. PubMed PMID: 22858820.

89. Espinoza-Gomez F, Hernandez-Suarez CM, Coll-Cardenas R. Educational campaign versus malathion spraying for the control of *Aedes aegypti* in Colima, Mexico. J Epidemiol Community Health. 2002;56(2):148-52. doi: 10.1136/jech.56.2.148. PubMed PMID: 11812816; PubMed Central PMCID: PMCPMC1732074.

90. Galvan J, Gutierrez L. Dengue Prevention in Merida, Yucatan, Mexico: Use of Formative Research to Refine an Education/Communication Intervention Targeting Household Management of Key *Aedes aegypti*-producing Containers. Dengue Bull. 2004;28.

91. Marina CF, Bond JG, Munoz J, Valle J, Chirino N, Williams T. Spinosad: a biorational mosquito larvicide for use in car tires in southern Mexico. Parasit Vectors. 2012;5:95. Epub 20120519. doi: 10.1186/1756-3305-5-95. PubMed PMID: 22608138; PubMed Central PMCID: PMCPMC3407511.

92. Martinez-Ibarra JA, Nogueda-Torres B, Meda-Lara RM, Montanez-Valdez OD, Rocha-Chavez G. Combining two teaching techniques for young children on *Aedes aegypti* control: effects on entomological indices in western Mexico. J Vector Ecol. 2012;37(1):241-4. doi: 10.1111/j.1948-7134.2012.00222.x. PubMed PMID: 22548559.

93. Anguiano-Moreno M, Aguayo-López MÁ, Álvarez-Lucas CH, Torres- Ornelas P, Lara-Esqueda A, Delgado-Enciso I. Segundo año de la Estrategia Estatal de Combate al Dengue en Colima. Med Int Mex. 2013;29:265-75.

94. Lorono-Pino MA, Garcia-Rejon JE, Machain-Williams C, Gomez-Carro S, Nunez-Ayala G, Najera-Vazquez Mdel R, et al. Towards a Casa Segura: a consumer product study of the effect of insecticide-treated curtains on *Aedes aegypti* and dengue virus infections in the home. Am J Trop Med Hyg. 2013;89(2):385-97. Epub 20130603. doi: 10.4269/ajtmh.12-0772. PubMed PMID: 23732254; PubMed Central PMCID: PMCPMC3741267.

95. Torres JL, Ordonez JG, Vazquez-Martinez MG. Dengue-related knowledge, attitudes, and practices in primary schools in Tapachula, Chiapas, Mexico. Rev Panam Salud Publica. 2014;35(3):214-8. PubMed PMID: 24793869.

96. Che-Mendoza A, Guillermo-May G, Herrera-Bojorquez J, Barrera-Perez M, Dzul-Manzanilla F, Gutierrez-Castro C, et al. Long-lasting insecticide-treated house screens and targeted treatment of productive breeding-sites for dengue vector control in Acapulco, Mexico. Trans R Soc Trop Med Hyg. 2015;109(2):106-15. doi: 10.1093/trstmh/tru189. PubMed PMID: 25604761; PubMed Central PMCID: PMCPMC4299524.

97. Manrique-Saide P, Che-Mendoza A, Barrera-Perez M, Guillermo-May G, Herrera-Bojorquez J, Dzul-Manzanilla F, et al. Use of insecticide-treated house screens to reduce infestations of dengue virus vectors, Mexico. Emerg Infect Dis. 2015;21(2):308-11. doi: 10.3201/eid2102.140533. PubMed PMID: 25625483; PubMed Central PMCID: PMCPMC4313634.

98. Tovar Zamora I, Ramos-Rodríguez A, Mendez-Galvan J, Servín-Villegas R. Entomological Indicators to Assess Larval *Aedes aegypti* (Linnaeus, 1762) Control in Baja

California Sur, México. Southwestern Entomologist. 2015;40:81-96. doi: 10.3958/059.040.0107.

99. Hernandez-Suarez CM, Mendoza-Cano O. Empirical evidence of the effect of school gathering on the dynamics of dengue epidemics. Glob Health Action. 2016;9:28026. Epub 20160106. doi: 10.3402/gha.v9.28026. PubMed PMID: 26743450; PubMed Central PMCID: PMCPMC4705256.

100. Jimenez-Alejo A, Morales-Perez A, Nava-Aguilera E, Flores-Moreno M, Apreza-Aguilar S, Carranza-Alcaraz W, et al. Pupal productivity in rainy and dry seasons: findings from the impact survey of a randomised controlled trial of dengue prevention in Guerrero, Mexico. BMC Public Health. 2017;17(Suppl 1):428. Epub 20170530. doi: 10.1186/s12889-017-4294-8. PubMed PMID: 28699555; PubMed Central PMCID: PMCPMC5506597.

101. Morales-Pérez A, Nava-Aguilera E, Legorreta-Soberanis J, Cortés-Guzmán AJ, Balanzar-Martínez A, Harris E, et al. "Where we put little fish in the water there are no mosquitoes:" a cross-sectional study on biological control of the *Aedes aegypti* vector in 90 coastal-region communities of Guerrero, Mexico. BMC Public Health. 2017;17(Suppl 1):433. Epub 20170530. doi: 10.1186/s12889-017-4302-z. PubMed PMID: 28699557; PubMed Central PMCID: PMCPMC5506569.

102. Che-Mendoza A, Medina-Barreiro A, Koyoc-Cardena E, Uc-Puc V, Contreras-Perera Y, Herrera-Bojorquez J, et al. House screening with insecticide-treated netting provides sustained reductions in domestic populations of *Aedes aegypti* in Merida, Mexico. PLoS Negl Trop Dis. 2018;12(3):e0006283. Epub 20180315. doi: 10.1371/journal.pntd.0006283. PubMed PMID: 29543805; PubMed Central PMCID: PMCPMC5870999.

103. Loroño-Pino MA, Uitz-Mena A, Carrillo-Solís CM, Zapata-Gil RJ, Camas-Tec DM, Talavera-Aguilar LG, et al. The Use of Insecticide-Treated Curtains for Control of *Aedes aegypti* and Dengue Virus Transmission in "Fraccionamiento" Style Houses in México. Journal of Tropical Medicine. 2018;2018:4054501. doi: 10.1155/2018/4054501.

104. Newton-Sanchez OA, de la Cruz Ruiz M, Torres-Rojo Y, Ochoa-Diaz-Lopez H, Delgado-Enciso I, Hernandez-Suarez CM, et al. Effect of an ecosystem-centered community

participation programme on the incidence of dengue. A field randomized, controlled trial. Int J Public Health. 2020;65(3):249-55. Epub 20200317. doi: 10.1007/s00038-020-01345-y. PubMed PMID: 32185417.

105. Devine GJ, Vazquez-Prokopec GM, Bibiano-Marin W, Pavia-Ruz N, Che-Mendoza A, Medina-Barreiro A, et al. The entomological impact of passive metofluthrin emanators against indoor *Aedes aegypti*: A randomized field trial. PLoS Negl Trop Dis. 2021;15(1):e0009036. Epub 20210126. doi: 10.1371/journal.pntd.0009036. PubMed PMID: 33497375; PubMed Central PMCID: PMCPMC7864418.

106. Manrique-Saide P, Herrera-Bojorquez J, Medina-Barreiro A, Trujillo-Pena E, Villegas-Chim J, Valadez-Gonzalez N, et al. Insecticide-treated house screening protects against Zika-infected *Aedes aegypti* in Merida, Mexico. PLoS Negl Trop Dis. 2021;15(1):e0009005. Epub 20210119. doi: 10.1371/journal.pntd.0009005. PubMed PMID: 33465098; PubMed Central PMCID: PMCPMC7853519.

107. Kroeger A, Lenhart A, Ochoa M, Villegas E, Levy M, Alexander N, et al. Effective control of dengue vectors with curtains and water container covers treated with insecticide in Mexico and Venezuela: cluster randomised trials. BMJ. 2006;332(7552):1247-52. doi: 10.1136/bmj.332.7552.1247. PubMed PMID: 16735334; PubMed Central PMCID: PMCPMC1471956.

108. Andersson N, Nava-Aguilera E, Arostegui J, Morales-Perez A, Suazo-Laguna H, Legorreta-Soberanis J, et al. Evidence based community mobilization for dengue prevention in Nicaragua and Mexico (Camino Verde, the Green Way): cluster randomized controlled trial. BMJ. 2015;351:h3267. Epub 20150708. doi: 10.1136/bmj.h3267. PubMed PMID: 26156323; PubMed Central PMCID: PMCPMC4495677.

109. Arostegui J, Coloma J, Hernandez-Alvarez C, Suazo-Laguna H, Balmaseda A, Harris E, et al. Beyond efficacy in water containers: Temephos and household entomological indices in six studies between 2005 and 2013 in Managua, Nicaragua. BMC Public Health. 2017;17(Suppl 1):434. Epub 20170530. doi: 10.1186/s12889-017-4296-6. PubMed PMID: 28699558; PubMed Central PMCID: PMCPMC5506593.

110. Parra C, Cernuzzi L, Rojas R, Denis D, Rivas S, Paciello J, et al. Synergies Between Technology, Participation and Citizen Science in a Community-Based Dengue Prevention Program. American Behavioral Scientist. 2020;64. doi: 10.1177/0002764220952113.

111. Machaca J, Llontop F, Pasapera F, Castañeda C. Mechanical removal of eggs of *Aedes aegypti* eradication of urban Dengue. Location Sechura - Piura, April-December 2001. Rev peru epidemiol 2002;10.

112. Dammert AC, Galdo JC, Galdo V. Preventing dengue through mobile phones: evidence from a field experiment in Peru. J Health Econ. 2014;35:147-61. Epub 20140305. doi: 10.1016/j.jhealeco.2014.02.002. PubMed PMID: 24681813.

113. Paredes-Esquivel C, Lenhart A, del Rio R, Leza MM, Estrugo M, Chalco E, et al. The impact of indoor residual spraying of deltamethrin on dengue vector populations in the Peruvian Amazon. Acta Trop. 2016;154:139-44. Epub 20151110. doi: 10.1016/j.actatropica.2015.10.020. PubMed PMID: 26571068.

114. Gunning CE, Okamoto KW, Astete H, Vasquez GM, Erhardt E, Del Aguila C, et al. Efficacy of *Aedes aegypti* control by indoor Ultra Low Volume (ULV) insecticide spraying in Iquitos, Peru. PLoS Negl Trop Dis. 2018;12(4):e0006378. Epub 20180406. doi: 10.1371/journal.pntd.0006378. PubMed PMID: 29624581; PubMed Central PMCID: PMCPMC5906025.

115. Lenhart A, Morrison AC, Paz-Soldan VA, Forshey BM, Cordova-Lopez JJ, Astete H, et al. The impact of insecticide treated curtains on dengue virus transmission: A cluster randomized trial in Iquitos, Peru. PLoS Negl Trop Dis. 2020;14(4):e0008097. Epub 20200410. doi: 10.1371/journal.pntd.0008097. PubMed PMID: 32275653; PubMed Central PMCID: PMCPMC7176142.

116. Basso C, Garcia da Rosa E, Romero S, Gonzalez C, Lairihoy R, Roche I, et al. Improved dengue fever prevention through innovative intervention methods in the city of Salto, Uruguay. Trans R Soc Trop Med Hyg. 2015;109(2):134-42. doi: 10.1093/trstmh/tru183. PubMed PMID: 25604764; PubMed Central PMCID: PMCPMC4299522.

117. Basso C, García da Rosa E, Lairihoy R, Caffera RM, Roche I, González C, et al. Scaling Up of an Innovative Intervention to Reduce Risk of Dengue, Chikungunya, and Zika Transmission in Uruguay in the Framework of an Intersectoral Approach with and without Community Participation. Am J Trop Med Hyg. 2017;97(5):1428-36. Epub 20170818. doi: 10.4269/ajtmh.17-0061. PubMed PMID: 28820690; PubMed Central PMCID: PMCPMC5817745.

118. Vivas E, Guevara De Sequeda M. A game as an educational strategy for the control of *Aedes aegypti* in Venezuelan schoolchildren. Rev Panam Salud Publica. 2003;14(6):394-401. doi: 10.1590/s1020-49892003001100004. PubMed PMID: 14769156.

119. Tun-Lin W, Lenhart A, Nam VS, Rebollar-Tellez E, Morrison AC, Barbazan P, et al. Reducing costs and operational constraints of dengue vector control by targeting productive breeding places: a multi-country non-inferiority cluster randomized trial. Trop Med Int Health. 2009;14(9):1143-53. Epub 20090714. doi: 10.1111/j.1365-3156.2009.02341.x. PubMed PMID: 19624476.

120. Vanlerberghe V, Villegas E, Oviedo M, Baly A, Lenhart A, McCall PJ, et al. Evaluation of the effectiveness of insecticide treated materials for household level dengue vector control. PLoS Negl Trop Dis. 2011;5(3):e994. Epub 20110329. doi: 10.1371/journal.pntd.0000994. PubMed PMID: 21468313; PubMed Central PMCID: PMCPMC3066153.

121. Sanchez L, Perez D, Perez T, Sosa T, Cruz G, Kouri G, et al. Intersectoral coordination in *Aedes aegypti* control. A pilot project in Havana City, Cuba. Trop Med Int Health. 2005;10(1):82-91. doi: 10.1111/j.1365-3156.2004.01347.x. PubMed PMID: 15655017.

122. Toledo ME, Vanlerberghe V, Baly A, Ceballos E, Valdes L, Searret M, et al. Towards active community participation in dengue vector control: results from action research in Santiago de Cuba, Cuba. Trans R Soc Trop Med Hyg. 2007;101(1):56-63. Epub 20060705. doi: 10.1016/j.trstmh.2006.03.006. PubMed PMID: 16824565.

123. Toledo Romani ME, Vanlerberghe V, Perez D, Lefevre P, Ceballos E, Bandera D, et al. Achieving sustainability of community-based dengue control in Santiago de Cuba. Soc Sci

Med. 2007;64(4):976-88. Epub 20061129. doi: 10.1016/j.socscimed.2006.10.033. PubMed PMID: 17140718.

124. Sanchez L, Perez D, Alfonso L, Castro M, Sanchez LM, Van der Stuyft P, et al. A community education strategy to promote participation in dengue prevention in Cuba. Rev Panam Salud Publica. 2008;24(1):61-9. doi: 10.1590/s1020-49892008000700008. PubMed PMID: 18764996.

125. Toledo ME, Baly A, Vanlerberghe V, Rodriguez M, Benitez JR, Duvergel J, et al. The unbearable lightness of technocratic efforts at dengue control. Trop Med Int Health. 2008;13(5):728-36. Epub 20080313. doi: 10.1111/j.1365-3156.2008.02046.x. PubMed PMID: 18346029.

126. Diaz C, Torres Y, Cruz AM, Alvarez AM, Piquero ME, Valero A, et al. An inter-sector participatory strategy in Cuba using an ecosystem approach to prevent dengue transmission at the local level. Cad Saude Publica. 2009;25 Suppl 1:S59-70. doi: 10.1590/s0102-311x2009001300006. PubMed PMID: 19287867.

127. Sanchez L, Perez D, Cruz G, Castro M, Kouri G, Shkedy Z, et al. Intersectoral coordination, community empowerment and dengue prevention: six years of controlled interventions in Playa Municipality, Havana, Cuba. Trop Med Int Health. 2009;14(11):1356-64. doi: 10.1111/j.1365-3156.2009.02379.x. PubMed PMID: 19840350.

128. Vanlerberghe V, Toledo ME, Rodriguez M, Gomez D, Baly A, Benitez JR, et al. Community involvement in dengue vector control: cluster randomised trial. BMJ. 2009;338:b1959. Epub 20090609. doi: 10.1136/bmj.b1959. PubMed PMID: 19509031; PubMed Central PMCID: PMCPMC2694260.

129. Toledo ME, Rodriguez A, Valdes L, Carrion R, Cabrera G, Banderas D, et al. Evidence on impact of community-based environmental management on dengue transmission in Santiago de Cuba. Trop Med Int Health. 2011;16(6):744-7. Epub 20110321. doi: 10.1111/j.1365-3156.2011.02762.x. PubMed PMID: 21418448.

130. Castro M, Sanchez L, Perez D, Carbonell N, Lefevre P, Vanlerberghe V, et al. A community empowerment strategy embedded in a routine dengue vector control programme:

a cluster randomised controlled trial. Trans R Soc Trop Med Hyg. 2012;106(5):315-21. Epub 20120331. doi: 10.1016/j.trstmh.2012.01.013. PubMed PMID: 22465423.

131. Sanchez L, Maringwa J, Shkedy Z, Castro M, Carbonell N, Van der Stuyft P. Testing the effectiveness of community-based dengue vector control interventions using semiparametric mixed models. Vector Borne Zoonotic Dis. 2012;12(7):609-15. Epub 20120112. doi: 10.1089/vbz.2011.0690. PubMed PMID: 22239179.

132. Zayas VM, Cruz LJ, Torres SA, Salinas DP. Intersectoral prevention of dengue in a health area of Santiago de Cuba. MediSan. 2012:175-81.

133. Montada Dorta D, Bisset Lazcano JA, Lezcano Rojas D, Castex Rodríguez M, Leyva Silva M, San Blas O, et al. Efectividad del Sipertrin 5sc en el control de *Aedes aegypti* en Santa Clara, Villa Clara. Revista Cubana de Medicina Tropical. 2013;65:350-60.

134. Toledo ME, Vanlerberghe V, Lambert I, Montada D, Baly A, Van der Stuyft P. No effect of insecticide treated curtain deployment on aedes infestation in a cluster randomized trial in a setting of low dengue transmission in Guantanamo, Cuba. PLoS One. 2015;10(3):e0119373. Epub 20150320. doi: 10.1371/journal.pone.0119373. PubMed PMID: 25794192; PubMed Central PMCID: PMCPMC4368727.

135. Hernández Y, Pérez Chacón D, Portal R, Polo V, Castro Peraza M. Abordaje de la comunicación para la prevención de arbovirosis en Cuba versus conocimientos, percepciones y prácticas de la población. Revista Cubana de Medicina Tropical. 2019;71.

136. Toledo ME, Vanlerberghe V, Rosales JP, Mirabal M, Cabrera P, Fonseca V, et al. The additional benefit of residual spraying and insecticide-treated curtains for dengue control over current best practice in Cuba: Evaluation of disease incidence in a cluster randomized trial in a low burden setting with intensive routine control. PLoS Negl Trop Dis. 2017;11(11):e0006031. Epub 20171108. doi: 10.1371/journal.pntd.0006031. PubMed PMID: 29117180; PubMed Central PMCID: PMCPMC5695847.

137. Lenhart A, Orelus N, Maskill R, Alexander N, Streit T, McCall PJ. Insecticide-treated bednets to control dengue vectors: preliminary evidence from a controlled trial in Haiti. Trop

Med Int Health. 2008;13(1):56-67. doi: 10.1111/j.1365-3156.2007.01966.x. PubMed PMID: 18291003.

138. Winch PJ, Leontsini E, Rigau-Perez JG, Ruiz-Perez M, Clark GG, Gubler DJ. Community-based dengue prevention programs in Puerto Rico: impact on knowledge, behavior, and residential mosquito infestation. Am J Trop Med Hyg. 2002;67(4):363-70. doi: 10.4269/ajtmh.2002.67.363. PubMed PMID: 12452490.

139. Barrera R, Amador M, Diaz A, Smith J, Munoz-Jordan JL, Rosario Y. Unusual productivity of *Aedes aegypti* in septic tanks and its implications for dengue control. Med Vet Entomol. 2008;22(1):62-9. doi: 10.1111/j.1365-2915.2008.00720.x. PubMed PMID: 18380655.

140. Barrera R, Amador M, Acevedo V, Caban B, Felix G, Mackay AJ. Use of the CDC autocidal gravid ovitrap to control and prevent outbreaks of *Aedes aegypti* (Diptera: Culicidae). J Med Entomol. 2014;51(1):145-54. doi: 10.1603/me13096. PubMed PMID: 24605464.

141. Barrera R, Amador M, Acevedo V, Hemme RR, Félix G. Sustained, area-wide control of *Aedes aegypti* using CDC autocidal gravid ovitraps. The American journal of tropical medicine and hygiene. 2014;91(6):1269-76. Epub 2014/09/15. doi: 10.4269/ajtmh.14-0426. PubMed PMID: 25223937.

142. Lorenzi OD, Major C, Acevedo V, Perez-Padilla J, Rivera A, Biggerstaff BJ, et al. Reduced Incidence of Chikungunya Virus Infection in Communities with Ongoing *Aedes Aegypti* Mosquito Trap Intervention Studies — Salinas and Guayama, Puerto Rico, November 2015–February 2016. 2016.

143. Barrera R, Acevedo V, Felix GE, Hemme RR, Vazquez J, Munoz JL, et al. Impact of Autocidal Gravid Ovitraps on Chikungunya Virus Incidence in *Aedes aegypt*i (Diptera: Culicidae) in Areas With and Without Traps. Journal of medical entomology. 2017;54(2):387-95. doi: 10.1093/jme/tjw187. PubMed PMID: 28031347.

144. Barrera R, Amador M, Munoz J, Acevedo V. Integrated vector control of *Aedes aegypti* mosquitoes around target houses. Parasit Vectors. 2018;11(1):88. Epub 20180208. doi:

10.1186/s13071-017-2596-4. PubMed PMID: 29422087; PubMed Central PMCID: PMCPMC5804079.

145. Juarbe-Rey D, Pérez AO, Santoni R, Ramírez MR, Vera M. Using Risk Communication Strategies for Zika Virus Prevention and Control Driven by Community-Based Participatory Research. Int J Environ Res Public Health. 2018;15(11). Epub 20181109. doi: 10.3390/ijerph15112505. PubMed PMID: 30423904; PubMed Central PMCID: PMCPMC6266788.

146. Barrera R, Harris A, Hemme RR, Felix G, Nazario N, Munoz-Jordan JL, et al. Citywide Control of *Aedes aegypti* (Diptera: Culicidae) during the 2016 Zika Epidemic by Integrating Community Awareness, Education, Source Reduction, Larvicides, and Mass Mosquito Trapping. J Med Entomol. 2019;56(4):1033-46. doi: 10.1093/jme/tjz009. PubMed PMID: 30753539; PubMed Central PMCID: PMCPMC6597296.

147. Sharp TM, Lorenzi O, Torres-Velasquez B, Acevedo V, Perez-Padilla J, Rivera A, et al. Autocidal gravid ovitraps protect humans from chikungunya virus infection by reducing *Aedes aegypti* mosquito populations. PLoS Negl Trop Dis. 2019;13(7):e0007538. Epub 20190725. doi: 10.1371/journal.pntd.0007538. PubMed PMID: 31344040; PubMed Central PMCID: PMCPMC6657827.

148. Harris AF, Sanchez Prats J, Nazario Maldonado N, Piovanetti Fiol C, García Pérez M, Ramírez-Vera P, et al. An evaluation of Bacillus thuringiensis israelensis (AM65-52) treatment for the control of *Aedes aegypti* using vehicle-mounted WALS® application in a densely populated urban area of Puerto Rico. Pest Manag Sci. 2021;77(4):1981-9. Epub 20210123. doi: 10.1002/ps.6227. PubMed PMID: 33314578; PubMed Central PMCID: PMCPMC8048509.

149. Chadee DD, Williams FL, Kitron UD. Impact of vector control on a dengue fever outbreak in Trinidad, West Indies, in 1998. Trop Med Int Health. 2005;10(8):748-54. doi: 10.1111/j.1365-3156.2005.01449.x. PubMed PMID: 16045461.

150. Chadee DD. Impact of pre-seasonal focal treatment on population densities of the mosquito *Aedes aegypti* in Trinidad, West Indies: a preliminary study. Acta Trop.

2009;109(3):236-40. Epub 20081209. doi: 10.1016/j.actatropica.2008.12.001. PubMed PMID: 19114025.

151. Nathan MB, Lloyd L, Wiltshire A. Community Participation in Environmental Management for Dengue Vector Control: Experiences from the English-speaking Caribbean. Dengue Bull. 2004.

152. USAID. The Zika AIRS Project (ZAP). Final Report. Rockville, MD: 2019.

153. McGregor BL, Connelly CR. A Review of the Control of *Aedes aegypti* (Diptera: Culicidae) in the Continental United States. J Med Entomol. 2021;58(1):10-25. doi: 10.1093/jme/tjaa157. PubMed PMID: 32829398; PubMed Central PMCID: PMCPMC7855389.

154. Bardach AE, Garcia-Perdomo HA, Alcaraz A, Tapia Lopez E, Gandara RAR, Ruvinsky S, et al. Interventions for the control of *Aedes aegypti* in Latin America and the Caribbean: systematic review and meta-analysis. Trop Med Int Health. 2019;24(5):530-52. Epub 20190311. doi: 10.1111/tmi.13217. PubMed PMID: 30771267.

155. Lima EP, Goulart MO, Rolim Neto ML. Meta-analysis of studies on chemical, physical and biological agents in the control of *Aedes aegypti*. BMC Public Health. 2015;15:858. Epub 20150904. doi: 10.1186/s12889-015-2199-y. PubMed PMID: 26341708; PubMed Central PMCID: PMCPMC4559884.

156. Marcos-Marcos J, Olry de Labry-Lima A, Toro-Cardenas S, Lacasana M, Degroote S, Ridde V, et al. Impact, economic evaluation, and sustainability of integrated vector management in urban settings to prevent vector-borne diseases: a scoping review. Infect Dis Poverty. 2018;7(1):83. Epub 20180903. doi: 10.1186/s40249-018-0464-x. PubMed PMID: 30173675; PubMed Central PMCID: PMCPMC6120095.

Supporting information

S1: Table Preferred Reporting Items for Systematic reviews and Meta-Analyses extension for Scoping Reviews (PRISMA-ScR) Checklist

S2: Table Listed essential concepts and keywords.

S3: Table Characteristics of studies identified in the Latin America region about *Ae. aegypti* and *Ae. albopictus* prevention and control interventions

S4: Table Characteristics of studies identified in the Caribbean region about *Ae. aegypti* and *Ae. albopictus* prevention and control intervention

S5: Table Characteristics of studies identified in the Latin America and Caribbean region about *Ae. aegypti* and *Ae. albopictus* prevention and control interventions

Figure 1

Click here to access/download;Figure;Fig 1 Prisma flow diagram.tif

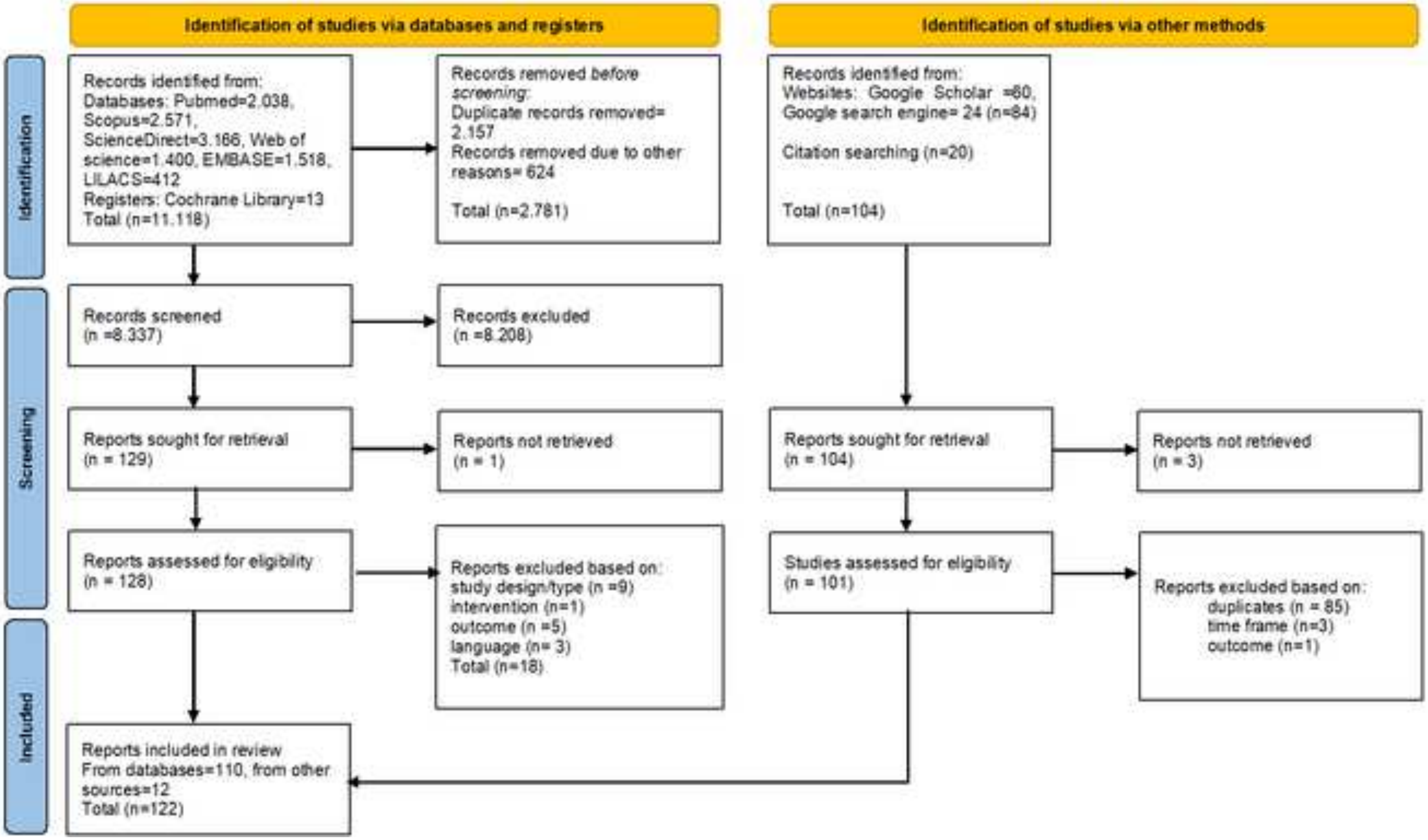

Figure 2

Click here to access/download;Figure;Fig 2 The number of publications per year (2000-2021).tif

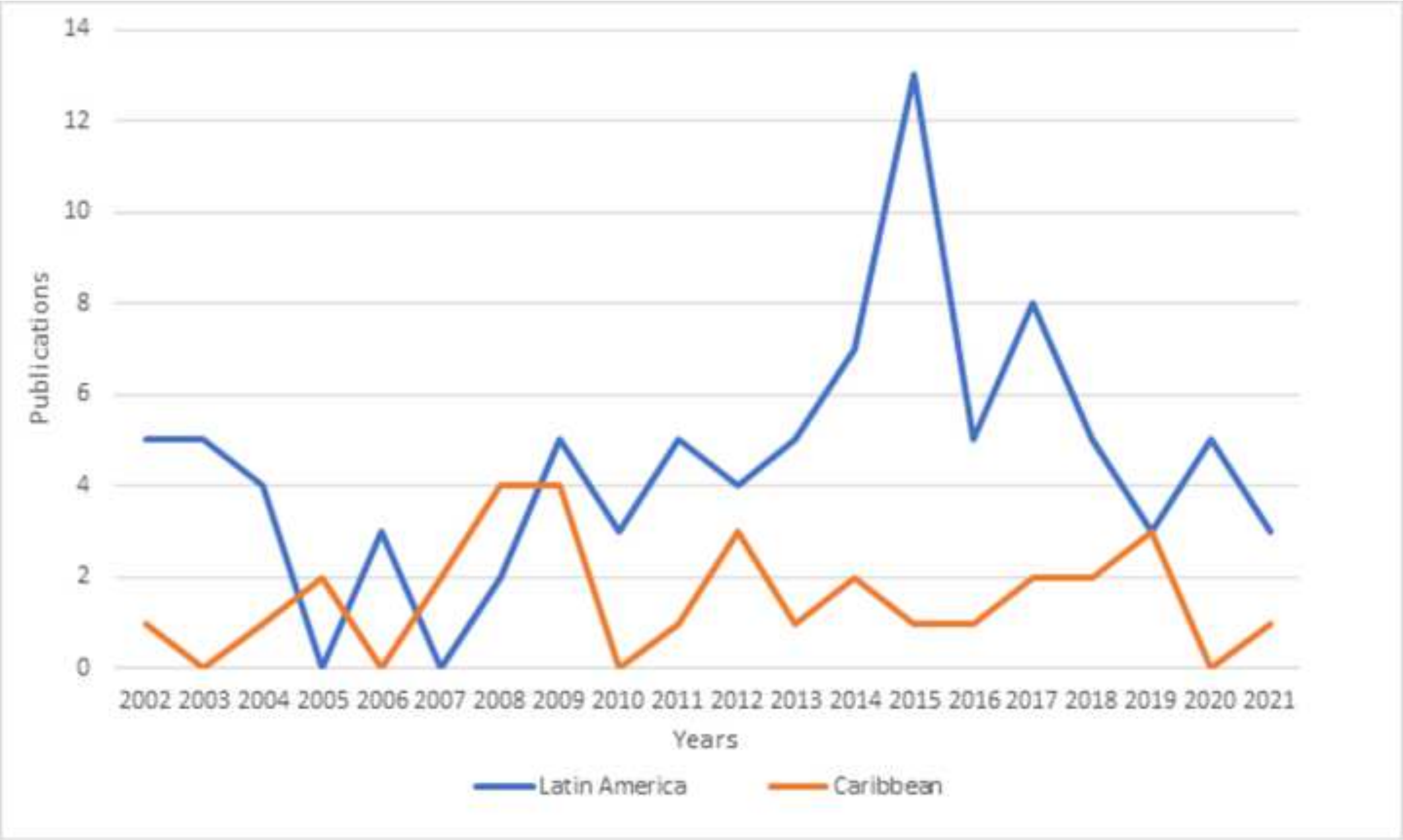

Click here to access/download
Supporting Information
S2 Table 2.docx

Click here to access/download
Supporting Information
S3 Table 3.docx

Click here to access/download
Supporting Information
S4 Table 4.docx

Click here to access/download
Supporting Information
S5 Table 5.docx

Click here to access/download
Supporting Information
S1 Table PRISMA-ScR-Checklist.docx
