# Supplementary material for: Effectiveness of Aedes-borne infectious disease control in Latin America and the Caribbean region: A scoping review
Source: PLoS One. 2022 Nov 2;17(11):e0277038. doi: 10.1371/journal.pone.0277038 (PMC9629598; doi:10.1371/journal.pone.0277038)
Supplement: Attachment — Submitted filename: Review PONE.docx [file pone.0277038.s006.docx]

Review PONE-D-22-16967

This is a very usefull and wellcome review work. It surveys different Aedes control strategies, including approaches that are currently gaining ground, related to the growing importance of encouraging popular participation, extrapolating the merely biomedical interventions, more classically used.

However, I suggest, since the Introduction, to give more emphasis to the fact that the quality of the articles was not verified. Some conclusions of the original authors are reproduced here, but sometimes there is doubt as to whether good parameters and controls were used. Some examples have been added below.

The Introduction is adequate and updated, with justified arguments, but it is worth mentioning structural determinants, which go beyond the health sector and which limit the scope of surveillance and entomological control.

In the Results section, in the item 'Interventions', when referring to references 51 and 55, the text says that the use of pyriproxyfen '... *led to a ten-fold decrease in adult mosquito emergence*...'. It is necessary to make it clear here that the difference is the strategy, not the insecticide: this is the effect from the 'disseminating stations' (‘*Mosquito-Disseminated Insecticide for Citywide Vector Contro*l').

Still in the item ‘Interventions’, there are issues that are certainly not addressed in the analysis in general review studies of this type. When mentioning, for example, reference 91, which deals with the application of different larvicides, the stability and persistence of Bti formulations in the field, and the resistance of the vector population to insecticides, notably temephos, would be relevant parameters for the analysis of the results.

Regarding the last paragraph of the item 'Interventions', concluding about the material presented, I reinforce the importance of mentioning the disseminating stations, in this specific case, so that it does not seem that pyriproxyfen is more efficient than the other larvicides presented. The aspect at issue in articles 51 and 55 is not simply the product, but the way of disseminating it in the treated area.

Regarding the item 'Health education and community mobilization/participation' - considering that this control component has increasing relevance, a comment on the formalization, or not, of information on *Aedes aegypti* in schools in different countries would be worth mentioning. Perhaps in grey literature... Did the authors find something relevant, formal, in this sense?

About Reference 70, the text says that *One study that assessed the impact of mass-media communication campaigns did not show beneficial results [70]*.' It seems a somewhat superficial conclusion, taking into account that, as mentioned in the Manuscript, the quality of articles has not been verified. In this specific case, what was the potential for publicizing the campaigns? Was it verified, for example, if the disclosure actually reached the affected people?

Still referring to the item ‘Health education and community mobilization/participation’, a comment: the number of articles from Cuba (121-124, 126-128, 130, 131) calls attention. The importance of popular participation in dengue prevention by means of Aedes control in this country is, in fact, already consolidated...

In fact, the importance of Cuba in relation to prevention actions, in general, is reiterated in the item 'Resources and capacity', in reference 123 (*Another study indicated that providing adequate basic utility services (e.g., garbage collection) to the communities could have a major bearing on the sustainability of the community-based mosquito control intervention, facilitating the successful implementation of the Aedes control program [123]*.'). These papers signal not only the stimulus to popular participation, but also the counterpart of public management – an aspect that would deserve to be highlighted.

In some cases, throughout the manuscript, anecdotal reports are generalized, as if they were related to an entire country; it would be usefull to verify throughout the text. For example, in the item 'Integrated interventions', about reference 45: *Another study that evaluated Brazil’s national dengue control plan attributed the program’s lack of success to insufficient household coverage [45]*.', it would be important to mention here that the article deals with the work in one municipality only.

Regarding reference 54: *The incorporation of the community mobilisation concept into the Brazilian national plan was linked with the reduction of the total number of dengue cases between the years 2009 and 2010 [54]*.' - there is a difference between the title in Portuguese and English in the original article. The article in Portuguese refers to only one municipality, Ipatinga. In table S3, it corresponds to article 24. The table is ok, it alludes to Ipatinga. But the main text is confusing, there is room for the interpretation that the result obtained is national in scope.

Still in the item 'Integrated interventions', when there is mention of a study in Mexico (reference 93), the statement *Dengue incidence decreased from 81.4% in 2010 to 79.1% in 2011 [93].*', seems to be a vague conclusion: one point is that differences are too small; moreover, the fact that dengue does not occur homogeneously between years is not being taken into account. For example, the text in table S3 (it is article 63) says that: ‘*The interventions reduced the peak of cases that had been recorded in the rainy season resulting from the transmission of dengue*’ - that is, it seems that the text of the present Manuscript assumes that (a) there is a significant reduction in the number of cases in the period and that (b) this reduction is a function of the intervention. However, it is known that there are many other factors that may be contributing to this scenario, which could be at least cited, so as not to appear that this conclusion is being shared here.

The first mention of the possibility of insecticide inefficiency is in the final, concluding paragraph of the item ‘Integrated interventions’. It is worth dealing with this aspect, in a generic way, since the introduction, explaining that this variable exists, but it was not addressed in the bibliographic survey, neither taken into account in the analyses.

In the Discussion, in relation to the statement *However, many factors such as insufficient funding, resources, workforce, and political priorities (e.g., prioritising SARS-CoV-2 over ABIDs) obstruct the implementation and maintenance of the IVM program nowadays*.' – about 'SARS-CoV-2 over ABIDs': what do you mean 'political priority'? That was a contingency. It is not a question here of opposing one disease to another, but of ensuring a more comprehensive view of health. Finally, it is important to take into account that the focus is not the mosquito.

The Discussion refers to article 149 to exemplify resistance to insecticides as a factor in reducing the effectiveness of the interventions. This article corresponds to reference 29 of the list in table S4. However, there is no mention of resistance in the table, nor in the abstract of the article. In practice, the only occasion this article, 149, deals with resistance is in its Discussion section, when it cites two other references, both from 1998.

In the Conclusions, the manuscript states that *The integrated Aedes control approach was more effective than a single strategy.*'. Considering that there was no evaluation of data quality of the articles, as explained in the paragraph before this one in the main text (‘limitations and strengths’), it is not clear how this conclusion was reached. It is quite true that this conclusion is, at least intuitively, desired. But the parameters evaluated here do not allow such conclusions. On the other hand, it seems relevant (and could be emphasized) that this work contributes to systematize, classify, or quantify the different methodologies approached; it would be possible, for example, to present, even graphically, the relative contribution of different countries, or regions, to each type of approach. Another option is to make it clear that the higher effectiveness of an integrated approach over single strategies was the major conclusion of the analyzed articles - and not of the authors of this manuscript.

In the Acknowledgments, the 'thank you in advance' calls attention: *We want to thank both the editors and reviewers for their insightful comments on the article.*'

Minor comment: I did not systematically check the bibliographic references, in relation to questions of form. However, I noticed that reference 34 (*Camargo Donalisio MR, Leite OF, Mayo RC, Pinheiro Alves MJC, de Souza A, Rangel O, et al. Use of Temephos for Control of Field Population of Aedes aegypti in Americana Sao Paulo, Brazil. 2002.*') is incomplete. It remains to add: ‘Dengue Bulletin. 2002 Dec; 26: 173-177'.
